# Anionic sigmatropic-electrocyclic-Chugaev cascades: accessing 12-aryl-5-(methylthiocarbonylthio)tetracenes and a related anthra[2,3-*b*]thiophene

**DOI:** 10.3762/bjoc.11.31

**Published:** 2015-02-20

**Authors:** Laurence Burroughs, John Ritchie, Mkhethwa Ngwenya, Dilfaraz Khan, William Lewis, Simon Woodward

**Affiliations:** 1 School of Chemistry, The University of Nottingham, University Park, Nottingham NG7 2RD, United Kingdom

**Keywords:** allenes, anionic Chugaev rearrangement, anionic sigmatropic rearrangement, tetracene properties, X-ray structures

## Abstract

1,4-Diols resulting from the double addition of ArCCLi (Ar = Ph, substituted phenyl, 2-thienyl) to *ortho*-C_6_H_4_(CHO)_2_ undergo cascades to tetracenes on simple admixture of LiHDMS, CS_2_ and MeI. Acene formation proceeds by [3,3]-sigmatropic rearrangement of xanthate anions followed by 6π electrocyclisations. The reactions are terminated by E2 or anionic Chugaev-type eliminations. Structural packing motifs and electronic properties are reported for the tetracenes.

## Introduction

**Scheme 1:**
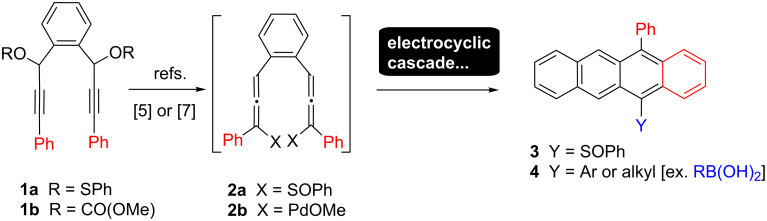
Use of bis-allene intermediates **2** for rapid access to substituted tetracenes [14,16].

In recent years polyacenes, especially tetra- and pentacenes, have been in the vanguard of new field effect and other organic electronic based devices [1–2]. Although the simple parent acenes have useful device characteristics in their own right, it is often desirable to be able to tune this performance by use of suitable substituted variants [3–4]. Unfortunately, attaining such derivatives rapidly through simple chemistry is often problematic [5–6]. Cross-coupling approaches (formally an excellent approach for acene library preparation) [7–13] are often hindered by the insolubility, or poor availability, of the parent haloacenes. Conversely, stepwise synthesis of a family of acene derivatives from various acyclic precursors is normally very step intensive. The prevalence of these issues in the synthesis of substituted tetracenes caused Lin [14], building on the anthracycline natural product work of Saá [15], to introduce a 1,2-bis-allene cascade approach for rapid access to tetracene sulfoxides in 2007 (Scheme 1).

**Scheme 2:**
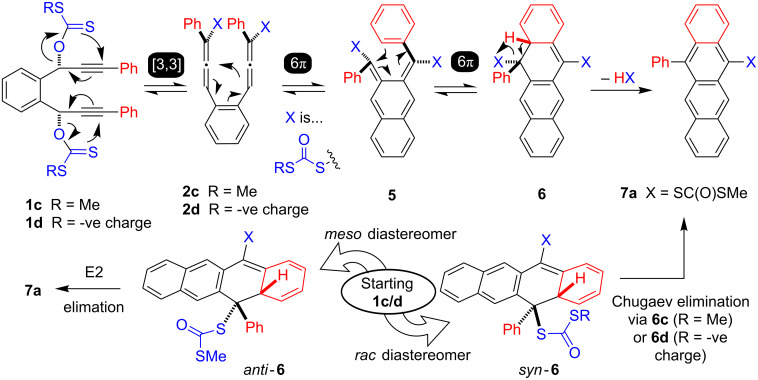
Proposed access to aryl substituted 5-thiolatotetracene derivatives.

In 2012 Liu used a Pd-based strategy to provide 12-substituted 5-aryltetracenes [after final trapping with RB(OH)_2_)] [16]. Both of these reactions rely on the formation of bis-allenes **2**, attained by Lin through 2,3-sigmatropic rearrangement of **1a** [14] or by S_N_2’ carbonate displacement in **1b** by Pd^0^(PPh_3_)_2_ in Liu’s case [16]. To circumvent reversibility of these pericyclic annulation strategies Lin relied on PhSOH elimination while Liu relied on ubiquitous palladium β-hydride steps leading to tetracenes **3** and **4**. We are interested in very efficient routes to tetracene derivatives containing one or more thiolate (SH) groups for the use in highly electrically conducting organics. In this regard we were attracted by a single result in the early literature [17] showing that traces of allenes related to **2** (X = SCOSMe) were accessible via nominal [3,3]-sigmatropic rearrangements of xanthates. As the thiocarbamate products derived from these are predicted to be easily hydrolised to thiolates this potentially offers a simple route to a protected SH analogue of **3**. Lin’s chemistry [14] cannot be used as no simple method to modify SOPh to SH is available. We proposed that use of starting material xanthate **1c** should provide suitably protected 5-thiotetracene derivatives directly (Scheme 2).

The required [3,3]-sigmatropic rearrangements and subsequent 6π elecrocyclisations of **1c** have precise stereochemical requirements (Scheme 2). Only the *meso* diastereomer of **1c** is predicted by Woodward–Hoffman analyses [18] to deliver *anti*-**6** that is required for facile E2 elimination leading to the desired tetracene **7** under thermal conditions. However, the initially required **1c** are typically attained as ca. 1:1 *rac*/*meso* mixtures and this might be expected to limit the potential yield of **7** to only 50% under simple heating (in the absence of other factors).

Houk has demonstrated that both electronic donor or acceptor and steric effects favour placing the larger/most electronically biased substituent ‘outwards’ in disrotatory 6π processes [19]. This might also depopulate **5** limiting the final yield of **7**. However, the following factors suggested to us the viability of Scheme 2: (i) traces of allenes have been observed when preparing xanthates from propargylic alcohols [17]; (ii) the relative van der Waals volumes of SOPh, Ph, CS_2_^−^ and C(=S)SMe (104.2, 76.9, 63.4, and 82.0 Å^3^, respectively [20]) and related electronic properties [σ(SOMe) +0.52, σ(Ph) +0.06, σ(SCOMe) +0.39 [21]] and the work of Lin [14] suggest that significant populations of intermediate **5**, with ‘inward Ph’ should be accessible; (iii) even if a *rac*-diol is used in the cascade, the possibility of aromatisation of **6** through Chugaev [22] *syn* elimination. Finally the system of Scheme 2 provides a unique opportunity to probe if these rearrangements do indeed proceed from the neutral xanthates **1c** or via the previously unprecedented **1d**–**2d**–**6d** anionic cascades.

## Results and Discussion

**Scheme 3:**
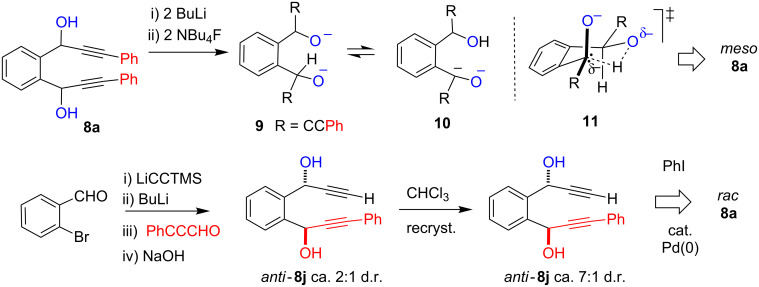
Equilibration to *meso* species.

Investigation of the chemistry of Scheme 2 commenced with the preparation of the required diols **8** through simple acetylide addition to *o*-phthalaldehyde (60–91% yield, see Supporting Information File 1). All of the additions proceeded in high yield, but under all conditions tried, no strong bias to either the *rac* or *meso* diastereomer could be realised. The *meso* enriched diastereomer of **8a** could be realised by treating *rac*/*meso* mixtures of bis-lithium alkoxide of **8a** with freshly prepared anhydrous NBu_4_F (2 equivalents) [23] (Scheme 3). Acid quench of the resultant purple dianion leads to ca. 5:1 *meso*:*rac* **8a**. We assign this transformation to an equilibrium between dialkoxide **9** and the benzylic anion **10**. Intramolecular proton delivery via cyclic transition state **11** is proposed to favour the *meso* dialkoxide prior to protonic quench. Samples of *rac* enriched **8a** were prepared from Sonogashira coupling of *anti* enriched **8j**. The latter could be prepared directly from *o*-bromobenzaldehyde as shown (Scheme 3) with ca. 1:7 *syn*:*anti* enrichment by recrystallisation from CHCl_3_. The enantiotopic ArCH signals of *rac*-**8a** are split into separate signals upon treatment with Eu(facam)_3_ confirming it to be the C_2_ chiral diastereomer while no equivalent splitting in ^1^H NMR samples of **8a** prepared from purple **11** (in line with it being the *meso* diastereomer). These assignments are in line with the finding of Saá [15]. The chemical shifts of the methine C*H*OH proton in *rac*-**8a** (δ_H_ 6.20) and *meso*-**8a** (δ_H_ 6.33) reflect an equivalent trend in diols **8b–f** where two distinct sets of equivalent signals are seen δ_H_ 6.14–6.20 and δ_H_ 6.23–6.35. On this basis we assign the higher chemical shift signal to the *meso* diastereomer.

Cascade optimisation (Table 1) was carried out using **8a** in THF unless otherwise stated. Typically diol **8a** (ca. 1:1 *rac*:*meso*) was deprotonated at an initial low temperature (*T*^1^), then treated sequentially with CS_2_ and MeI before finally being brought to a second higher temperature (*T*^2^) to facilitate aromatisation leading to **7a** (see Supporting Information File 1 for full optimisation details). Simply allowing −78 °C solutions of the dialkoxide to warm slowly to ambient temperature in the presence of excess CS_2_/MeI provided small amounts of tetracene **7a** (Table 1, run 1). Formation of the xanthate is favoured at −30 °C but this is slowed at −50 °C (Table 1, runs 2 and 3). The IR ν_max_ [cm^−1^] (*rac*-**1c**: 1035; *meso*-**1c**: 1036) of run 2 are consistent with the isolation of xanthate [17].

**Table 1:** Optimisation of yield of tetracene **7a**.^a^

Run	Base	*rac*:*meso*^b^	*T*^1^ [°C]	*T*^2^ [°C]	**1c** [%]^c^	**7a** [%]^c^

1	NaH	1:1.1	−78	22	23	9
2	NaH	1:1.1	−30	40	95	–
3	NaH	1:1.1	−50	40	45	30
4	NaH	1:1.1	0	60	30	43
5	LiHDMS	1:1.1	−50	40	39	38
**6**	**LiHDMS**	**1:1.1**	**0**	**60**	**30**^b^	**60**
7	KHDMS	1:1.1	0	60	9	50
8	LiHDMS	1:4	0	60	5^b^	38
**9**	**LiHDMS**	**8:1**	**0**	**60**	**5**^b^	**89**

^a^Using **8a** (0.45 mmol) in THF (5.0 mL), with base (2.0 equiv), CS_2_ (3.0 equiv), MeI (8.0 equiv), see Supporting Information File 1 for details. ^b^Determined by NMR spectroscopy. ^c^Isolated yields, except where noted.

All attempts to convert the neutral xanthate **1c** (either *rac* or *meso* from Table 1, run 2) to tetracene **7a** under thermal or photochemical conditions failed. Either **1c** was recovered, or it slowly decomposed under forcing conditions (>200 °C; or 180–365 nm Hg lamp). Exceptionally, traces of **7a** were detected in reactions eletro-catalysed by Bauld’s catalyst (tris(4-bromophenyl)ammoniumyl hexachloroantimonate) [24] at 25 °C but these showed very poor chemoselectivity. Conversely, rapid one-pot heating of a mixture of all the reaction components maximises the yield of **7a** (Table 1, runs 3–6). These results very strongly suggest unprecedented anionic [3,3]-sigmatropic rearrangement starting from **1d**; another addition to the body of evidence for the importance of charge upon sigmatropic rearrangements [25–26]. In the subsequent cascade the second 6π electrocyclisation appears rate limiting. The yield of **7a** in run 6 (60%) indicates conversion via *syn*-**6d** (unprecedented anionic Chugaev elimination) is possible to some extent. If only E2 termination of the cascade was possible (i.e., via *anti*-**6**) a maximum yield of 52% **7a** should be realised from the 1:1.1 *rac*:*meso* sample of **8a** used. This idea is strongly supported by runs 8 and 9 and the observation that replacing MeI with other alkylating agents (EtBr, BnBr) resulted in only traces of tetracenes. Of the bases screened (see Supporting Information File 1), LiHDMS was superior, only its potassium analogue gave comparable performance (Table 1, run 7).

**Scheme 4:**
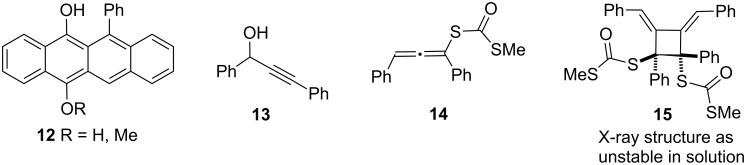
Competing reaction pathways.

In all reactions of Table 2 there is some unrecovered material. One common byproduct is an intensely red compound detected at high *R*_f_ (0.82, 4:1 pentane/CH_2_Cl_2_) in TLC analyses conducted under argon. The very high air sensitivity of this compound prevents its characterisation but it is tentatively ascribed to a mixture (**12**, Scheme 4) of hydroquinone and its monomethylether on the basis of partial ^1^H NMR spectrum and ESI mass spectra.

**Table 2:** Preparation of derivatives.^a^

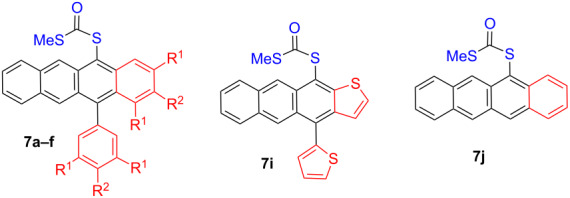				

Compound	Precursor diol (**8**) *rac*:*meso* ratio	R^1^	R^2^	Yield (**7**) [%]

**7a**	1.6:1.0	H	H	85
**7b**	1.9:1.0	OMe	OMe	47
**7c**	4.8:1.0	CF_3_	H	56
**7d**	**1.8:1.0**	**OMe**	**H**	**>99**
**7e**	1.6:1.0	F	H	29
**7f**	1.0:1.1	H	CF_3_	44
**7g**	1.0:1.2	H	OMe	38
**7h**	1.0:1.6	H	*t-*Bu	22
**7i**	1.0:1.0	–	–	38
**7j**	1.0:2.0^b^	–	–	50

^a^From diol precursor (0.45 mmol) in THF (5.0 mL), with LiHDMS (2.0 equiv), CS_2_ (3.0 equiv), MeI (8.0 equiv), isolated yields. ^b^Equivalent *anti*:*syn* ratio for **8j**.

The poorly performing runs of Table 1 also show a broad isolable red band (*R*_f_ ca. 0.18, 4:1 pentane/CH_2_Cl_2_) whose ^1^H and ^13^C NMR and MS data were intractable. For example, the ^1^H NMR spectrum shows only a broad envelope of signals at δ_H_ 7.95–7.20; a number of very similar isomeric species seem to be present. To cast light on these issues model alcohol **13** was treated with NaH/CS_2_/MeI at −78 °C and the mixture allowed to come to ambient temperature. This resulted in the smooth formation of allene **14**. In particular, the presence of allenic and C=O signals in the ^13^C NMR spectrum at 212.1 and 188.5 ppm and the absence of any alkyne C≡C resonances in the region δ_C_ 80–90 are indicative of this transformation. All attempts to isolate **14** resulted only in the rapid formation of red oils whose mass spectra show molecular ions at (**14**)*_n_* (*n* = 1–3). Attempted direct crystallisation provided only trace amounts of cyclobutene **15** which is otherwise unstable in solution (see Supporting Information File 1). Its extensive decomposition prevented other characterisation. Based on this model system, it is proposed that intermolecular [2 + 2] reactions of bis-allenes, similar to results in other reported allenic rearrangements to rubrene [27–28], **2c,d** result in the formation of numereous stereoisomeric oligomers resulting in the broad uninformative NMR spectra in the cascade byproducts. The structure of **15** is unremarkable except for the presence of a highly elongated C–C bond (1.63 Å) brought on through the steric congestion of the adjacent quaternary centers. A similar situation has been reported [29].

The use of the optimal conditions provided a series of acene derivatives (Table 2). All reactions resulted in chromatographically stable red microcrystalline solids. As anionic Chugaev elimination appeared the preferred aromatisation route from the studies of Table 1 (compare runs 6, 8 and 9), preparations of **7a–c** strongly benefit from higher *rac*:*meso* ratios that increase the population of the equivalent *syn*-**6** intermediates (Scheme 2). Steric congestion in the anion Chugaev transition state appears to favour this as all these compounds are isolated in good to excellent yields. Conversely **7e–h** are isolated in lower yields due to a combination of higher *meso* content in **8e–h** (leading less efficient E2 elimination) and lower steric promotion in the anion Chugaev elimination. Steric, rather than electronic, factors seem to affect the reaction most as evidenced by the quantitative yield of **7d** compared to **7b** (47%), **7c** (56%), **7f** (44%) and **7g** (38%). The decreasing yields suggest that *meta* substitution promotes the 6π cyclisation while *para* electronic affects are minor and unhelpful according to the observed trend. Increasing the reaction temperature, in attempts to facilitate E2 elimination, was generally not useful as this led only to increased amounts of inert xanthates through sulfur alkylation. However, in the case of **7h** this approach did allow us to reach 50 ± 4% yields (range for 6 runs).

**Scheme 5:**
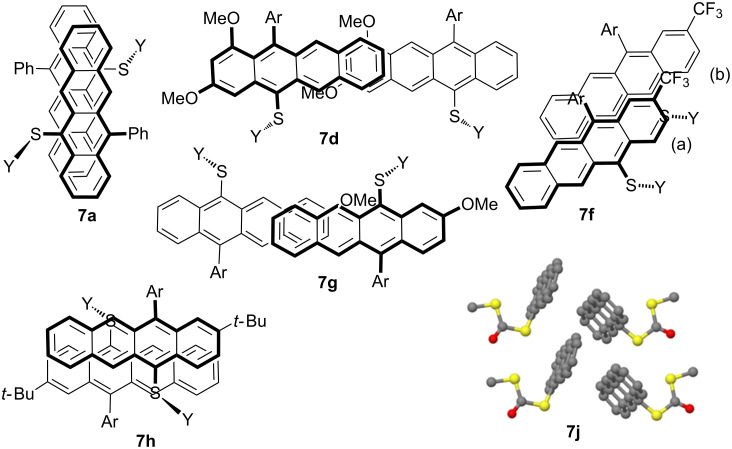
Stacking motifs in **7a**, **d**, **f**–**h** and **j**. Y = COSMe.

Compounds **7a**, **d**, **f–h** and **j** were subjected to single-crystal X-ray crystallography. This confirmed the molecular connectivity but more importantly allowed insight into their crystal packing features (Scheme 5 and Supporting Information File 1) across the family of structures. Pairs of **7a** associate with slip-stack pairing (C_π_···C_π_ 3.51–3.72 Å). Each of these (**7a**)_2_ ‘dimers’ is linked to the next through π contacts to the xanthate methyl (C_π_···MeS 3.38 Å). The ‘gaps’ in the columns are filled by an additional motif (C_π_···C_π_ 3.32–3.59 Å) almost perpendicular to the stacking. In **7d** a lattice of (**7d**)*_n_* chains propagates through C(11)_π_···MeS (3.39 Å) contacts. Adjacent chains overlap to produce the partial brickwork stack motif showing C_π_···C_π_ 3.51–3.60 Å between the most electron rich and deficient aryl rings. Offset stacking ribbons are found in **7f** (i.e., graphic ‘a’ is above ‘b’, etc.). The closest contacts are C···C_edge_ at 3.82–3.96 Å and C_π_···F–CF_2_ 3.2 Å. Perpendicular ribbons propagate through the crystal linked by inter-digitated xanthates or CF_3_ groups. Structure **7f** is the only one of the di/trisubstituted family not to show local *C*_2_ symmetry in intermolecular paring of the tetracenes. The structures of **7g**,**h** (Scheme 5) are closely related to those of **7d** and **7a**, respectively. Finally, the least substituted tetracene **7j** forms ribbons of herringbone structures.

Estimates of the HOMO–LUMO data for **7** were taken from UV and CV measurements (see Table 3 and Supporting Information File 1), as well as by DFT calculations. Tetracenes **7d** and **7f** show the widest range in HOMO–LUMO perturbation while *E*_g_ opt. is ca. 0.4 eV lower that *E*_g_ calcd. across the series. We could not attain the reduction potentials of **7** but from the onset of oxidation data we could estimate the HOMO energy levels in **7** and these followed the same trend as *E*_HOMO_ calcd. Preliminary testing of vacuum deposited thin polycrystalline films (ca. 800 nm) of **7a** and **7j** showed dielectric behaviour (σ <10^−10^ S cm^−1^) indicating that additional derivitisation and radical cation salt formation is required for the attainment of high electrical conductivity, as in the case of tetrathiotetracene [30].

**Table 3:** Electro-optic properties of **7a–j**.

Compound	E_½_ (ox.) [V]^a^	HOMO/ LUMO calcd. [eV]^b^	ν_max_ (vis) [nm]^c^	*E*_g_ opt. [eV]^d^	*E*_g_ calcd. [eV]^b^

**7a**	+0.52	−5.16/−2.51	287	2.27	2.65
**7b**	+0.22	−5.21/−2.57	297	2.23	2.63
**7c**	+1.07	−5.23/−2.93	297	2.18	2.60
**7d**	+0.29	−4.89/−2.36	296	2.09	2.55
**7e**	+0.65	−5.58/−2.96	286	2.27	2.62
**7f**	+0.76	−5.61/−2.98	290	2.24	2.63
**7g**	+0.43	−5.20/−2.69	290	2.28	2.51
**7h**	+0.55	−5.33/−2.69	289	2.26	2.65
**7i**	+0.60	−5.27/−2.32	283	2.49	2.95
**7j**	+0.62	−5.10/−2.40	283	2.36	2.70

^a^By cyclic voltammetry, referenced against Fc/Fc^+^. ^b^DFT: Calculated with the B3LYP-6-31G(d,p) basis set using Gaussian 09 Rev.D.01. ^c^In CH_2_Cl_2_. ^d^Determined from the onset (Tauc) of the lowest energy visible absorption band.

## Conclusion

Typical [3,3]-sigmatropic rearrangements of xanthates are normally considered to proceed via neutral species (such as **1c**). The tetracenes **7** herein are not formed this way, instead the evidence here strongly suggests that the required [3,3]-6π–6π electrocyclic cascades takes place via anionic xanthate species before final capping with methyl iodide. Final aromatisation through E2 or the anionic equivalent of the Chugaev reaction are also both viable. As neutral Chugaev reactions normally require very high temperatures this alternative approach is attractive as only moderate temperatures are required (60–80 °C). This procedure allows rapid access to mono sulfur-containing acenes, and is applicable to small scale library synthesis. Only low cost reagents are required and otherwise difficult to synthesise hindered 1,3,4,12-tetrasubstituted species can be made straightforwardly.

## Supporting Information

File 1 Experimental procedures, characterisation data, X-ray structures, data for the DFT calculations, and NMR spectra.
