# Supplementary material for: Anionic sigmatropic-electrocyclic-Chugaev cascades: accessing 12-aryl-5-(methylthiocarbonylthio)tetracenes and a related anthra[2,3-b]thiophene
Source: Beilstein J Org Chem. 2015 Feb 20;11:273–9. doi: 10.3762/bjoc.11.31 (PMC4362017; doi:10.3762/bjoc.11.31)
Supplement: File 1 — Experimental procedures, characterisation data, X-ray structures, data for the DFT calculations, and NMR spectra. [file Beilstein_J_Org_Chem-11-273-s001.pdf]

Supporting Information

for

Anionic sigmatropic-electrocyclic-Chugaev cascades: accessing 12-aryl-5-(methylthiocarbonylthio)tetracenes and a related anthra[2,3-*b*]thiophene

Laurence Burroughs, John Ritchie, Mkhethwa Ngwenya, Dilfaraz Khan, William Lewis and Simon Woodward*

Address: School of Chemistry, The University of Nottingham, University Park, Nottingham NG7 2RD, United Kingdom

Email: Simon Woodward - simon.woodward@nottingham.ac.uk

* Corresponding author

Experimental procedures, characterisation data, X-ray structures, data for the DFT calculations, and NMR spectra

Contents

1. General information S1

2. Preparation of diol starting materials **8** S2

2.1 Representative preparation of higher cost acetylenes S2

3,5-Dimethoxyphenylacetylene S2

1-Ethynyl-3,4,5-timethoxybenzene S2

2.2 General procedure for preparation of the 1,4-diols **8** S3

1,1'-(1,2-Phenylene)bis(3-phenylprop-2-yn-1-ol) (**8a**) S3

Stereochemical enrichment of 1,1'-(1,2-phenylene)bis(3-phenylprop-2-yn-1-ol) (**8a**) S3

1,1'-(1,2-Phenylene)bis(3-(3,4,5-trimethoxyphenyl)prop-2-yn-1-ol) (**8b**) S4

1,1'-(1,2-Phenylene)bis(3-(3,5-bis(trifluoromethyl)phenyl)prop-2-yn-1-ol) (**8c**) S5

1,1'-(1,2-Phenylene)bis(3-(3,5-dimethoxyphenyl)prop-2-yn-1-ol) (**8d**) S5

1,1'-(1,2-phenylene)bis(3-(3,5-difluorophenyl)prop-2-yn-1-ol) (**8e**) S6

1,1'-(1,2-Phenylene)bis(3-(4-(trifluoromethyl)phenyl)prop-2-yn-1-ol) (**8f**) S6

1,1'-(1,2-Phenylene)bis(3-(4-methoxyphenyl)prop-2-yn-1-ol) (**8g**) S7

1,1'-(1,2-Phenylene)bis(3-(4-tert-butylphenyl)prop-2-yn-1-ol) (**8h**) S7

1,1'-(1,2-Phenylene)bis(3-(thiophen-2-yl)prop-2-yn-1-ol) (**8i**) S7

1-(2-(1-Hydroxyprop-2-yn-1-yl)phenyl)-3-phenylprop-2-yn-1-ol (**8j**) S8

3. Preparation of tetracenes and anthra[2,3-*b*]thiophene **7a–j** S9

3.1 Optimisation of one-pot tetracene preparation........S9
3.2 General procedure for formation of tetracenes **7** and antrathiophene (**7h**)........S9
5-(Methylthiocarbonylthio)-12-phenyltetracene (**7a**)........S10
5-(Methylthiocarbonylthio)-1,2,3-trimethoxy-12-(1,2,3-trimethoxyphenyl)tetracene (**7b**)........S10
1,3-Bis(trifluoromethyl)-12-((3,5-bis(trifluoromethyl)phenyl)-5-(methylthiocarbonylthio)-tetracene (**7c**)........S10
1,3-Dimethoxy-12-(3,5-dimethoxyphenyl)-5-(methylthiocarbonylthio)tetracene (**7d**)........S11
1,3-Difluoro-12-(3,5-difluorophenyl)-5-(methylthiocarbonylthio)tetracene (**7e**)........S11
5-(Methylthiocarbonylthio)-2-(trifluoromethyl)-12-(4-(trifluoromethyl)phenyl)tetracene (**7f**)........S12
2-Methoxy-12-(4-methoxyphenyl)-5-(methylthiocarbonylthio)tetracene (**7g**)........S12
2-*tert*-Butyl-12-(4-*tert*-butylphenyl)-5-(methylthiocarbonylthio)tetracene (**7h**)........S13
11-(Methylthiocarbonylthio)-4-(thiophen-2-yl)anthra[2,3-*b*]thiophene (**7i**)........S13
5-(Methylthiocarbonylthio)tetracene (**7j**)........S13
3.3 By-products and control reactions........S14
1,3-Diphenyl-5-(methylthiocarbonylthio)propa-1,2-diene (**14**) and (3,4-di((*Z*)-benzylidene)-(1,2-(methylthiocarbonylthio)cyclobutane-1,2-diyl)dibenzene (**15**)........S14
4. Characterisation of electro-optic properties of acenes **7**........S16
4.1 Cyclic voltammetry studies........S16
4.2 Optical studies........S17
4.3 DFT calculations........S17
5. Crystallographic studies of acenes **7** and cyclobutane **15**........S18
5-(Methylthiocarbonylthio)-12-phenyltetracene (**7a**)........S18
1,3-Dimethoxy-12-(3’,5’-dimethoxyphenyl)-5-(methylthiocarbonylthio)tetracene (**7d**)........S18
4-(Methylthiocarbonylthio)-2-(trifluoromethyl)-12-(4-(trifluoromethyl)phenyl)tetracene (**7f**)........S19
2-Methoxy-12-(4-methoxyphenyl)-5-(methylthiocarbonylthio)tetracene (**7g**)........S20
2-*tert*-Butyl-12-(4-*tert*-butylphenyl)-5-(methylthiocarbonylthio)tetracene (**7h**)........S20
5-(Methylthiocarbonylthio)tetracene (**7j**)........S21
Comparisons between the structures **7**........S22
(3,4-di((Z)-benzylidene)-(1,2-(methylthiocarbonylthio)cyclobutane-1,2-diyl)dibenzene (**15**)........S23
6. Spectroscopic data (all compounds)........S24
7. References and notes........S54

1. General information

All reactions were carried out under argon atmosphere using a flame-dried Schlenk apparatus. Dichloromethane was used distilled from calcium hydride; diethyl ether, dimethoxyethane and tetrahydrofuran were used distilled from sodium/benzophenone ketyl. Alkynes were purchased from Sigma-Aldrich, Alfa-Aesar and Maybridge and were distilled under reduced pressure before use. High cost alkynes were also prepared by Corey–Fuchs procedures; the preparation given for 3,5-dimethoxyphenylacetylene is representative. Phenylpropargylaldehyde was prepared by a literature procedure.[1] Phthaldialdehyde was purchased from Alfa-Aesar; hexamethyl disilazane, carbon disulfide and iodomethane were purchased from Sigma-Aldrich and were dried (A4 sieves) as necessary. Organolithium reagents were Gilman double titrated before use. All temperatures referred to those of the external oil baths used. Thin layer chromatography was performed on foil-backed plates coated with Merck Silica gel 60 F_{254}. The plates were developed using ultraviolet light and basic aqueous potassium permanganate. Preparative thin layer chromatography was performed using Analtech 1000 µm UV254 pre-coated plates. Liquid chromatography was performed using forced flow (flash column) with the solvent systems indicated. The stationary phase used was silica gel 60 (220–240 mesh) supplied by Fluorochem. Infrared spectra were recorded on a Bruker Tensor 27 FTIR spectrometer using NaCl plates (films were formed by evaporation of chloroform) or a Perkin–Elmer 1600 FT-IR in solution cells. Solution UV–vis spectra were recorded on a Bruker Lambda 25 instrument. Nuclear magnetic resonance spectra were recorded on Bruker DPX-400 (400.2 MHz), AV400 (400.1 MHz), AV(III)400 (400.1 MHz) or AV(III)500 (500.1 MHz) spectrometers at ambient temperature. Chemical shifts are quoted in parts per million (ppm) and were referenced as follows: chloroform-*d*, 7.26 ppm, methanol-d_4, 4.87 ppm for ^{1}H NMR data; and for ^{13}C NMR data: chloroform-*d*, 77.16 ppm, methanol-d_4, 49.00.[2] Coupling constants (*J*) are quoted in Hertz and coupling correlations were based on standard COSY, DEPT, HMQC, HMBC experiments. Mass spectrometry was performed using a VG Micromass AutoSpec spectrometer (EI) or Bruker MicroTOF (ESI) as noted. Melting points were determined with a Stuart Scientific SMP3 melting point apparatus.

2. Preparation of diol starting materials 8

2.1 Representative preparation of higher cost acetylenes

3,5-Dimethoxyphenylacetylene

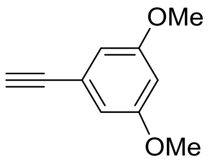

(i) Preparation of dibromoalkene. Freshly sublimed CBr_4 (6.39 g, 19.3 mmol, 2 equiv) was dissolved in dry CH_2Cl_2 (20 mL) and cooled 0 °C. Triphenylphosphine (10.1 g, 38.5 mmol, 4 equiv) in dry CH_2Cl_2 (20 mL) was added and the mixture left stirring for 30 min. Solid 3,5-methoxybenzaldehyde (1.61 g, 9.69 mmol, 1 equiv) dissolved in CH_2Cl_2 (20 mL) was added dropwise and the reaction monitored by TLC indicated completion (product R_f 0.74, 7:3 pentane:Et_2O). The resultant mixture was quenched with a 1:1 mixture of CH_2Cl_2:H_2O (75 mL), extracted with CH_2Cl_2 (3 × 30 mL), dried ($MgSO_4$), filtered and reduced in vacuo, giving a solid yellow crude (16.4 g). Purification by flash column chromatography (1:9 CH_2Cl_2:pentane) give a colourless powder (2.99 g, 97%), R_f 0.74 (7:3 pentane:ether), m.p. 74–75 °C. ^{1}H NMR (400.1 MHz, $CDCl_3$): δ 7.45 (s, 1H, CC*H*), 6.72 (d, *J* = 2.1 Hz, 2H, Ar*H*), 6.49 (t, *J* = 2.1 Hz, 1H, Ar*H*), 3.83 (s, 6H, OC*H*$_3$). ^{13}C NMR (100.6 MHz, $CDCl_3$): δ 160.7 (C), 137.0 (C), 136.9 (CH), 106.5 (CH), 100.9 (CH), 90.0 (C), 55.4 (CH_3). MS ESI calcd. for $C_{10}H_{10}O_2Br_2$ *m/z* expected 320.9120 (M+H), *m/z* found 320.9110 (M+H).
(ii) Corey-Fuchs procedure. Solid 1-(2,2-dibromovinyl)-3,5-dimethoxybenzene (2.99 g, 9.28 mmol, 1 equiv) was dissolved in dry THF (50 mL) under argon with stirring at −50 °C (dry ice/acetonitrile bath). A solution of *n*BuLi (24.0 mL, 1.6 M in hexanes, 38.4 mmol, 4.1 equiv) was added dropwise after which TLC indicated completion of the reaction. The mixture was quenched with saturated ammonium chloride (40 mL), extracted with CH_2Cl_2 (3 × 20 mL), dried ($NaSO_4$), filtered and evaporated, giving an orange brown oil (1.61 g). Purification by flash column chromatography (pure pentane, followed by 19:1 pentane:Et_2O) gave a pure colourless crystalline solid (1.22 g, 81%), R_f = 0.24 (pentane), m.p. 46–47 °C with literature properties.[3] ^{1}H NMR (400.1 MHz, $CDCl_3$): δ 6.72 (d, *J* = 2.3 Hz, 2H, Ar*H*), 6.50 (t, *J* = 2.3 Hz, 1H, Ar*H*), 3.76 (s, 6H, OC*H*$_3$), 3.15 (s, 1H, CC*H*). ^{13}C NMR (100.6 MHz, $CDCl_3$): δ 160.6 (C), 123.4 (C), 110.0 (CH), 102.3 (CH), 83.7 (C), 76.9 (CH), 55.4 (CH_3).

1-Ethynyl-3,4,5-trimethoxybenzene

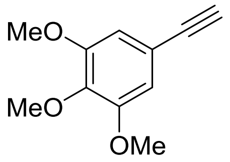

i) Sublimed CBr_4 (6.43 g) was added to a flame dried schlenk flask of dry dichloromethane (67 mL) and stirred at 0 °C. Triphenylphosphine (10.47 g) was then added and stirred for 30 minutes. 3,4,5-trimethoxybenzaldehyde was then added in one portion and stirred. When TLC indicated completion, the reaction was quenched with 1:1 dichloromethane: water, extracted with dichloromethane, dried with $MgSO_4$, filtered and concentrated under reduced pressure giving a crude yellow solid (19.6 g). The crude was purified by flash column chromatography (7:3 pentane:diethyl ether) giving a colourless crystalline solid (3.30g, 94%). R_f 0.31 (7:3 pentane:diethyl ether), m.p. 60–62 °C, ^{1}H NMR (400.2 mHz): δ 7.36 (s, 1H, C=C*H*), 6.76 (s, 2H, Ar*H*), 3.83 (s, 3H, OC*H*$_3$), 3.80 (s, 6H, OC*H*$_3$); ^{13}C NMR (100.6 MHz, $CDCl_3$) δ 152.7 (C), 138.1 (C), 136.4 (CH), 130.1 (C), 105.5 (C), 88.2 (CH), 60.4 (CH_3), 55.8 (CH_3), IR ($CHCl_3$) ν_{max}/cm^{-1} 3011, 2965, 2940, 2916, 2839, MS (+ESI) calcd. for $C_{11}H_{12}O_3Br_2$ *m/z* 350.9226 (M+H), found *m/z* 350.9239 (M+H).
ii) *n*-Butyllithium (22.5 mL, 1.6 M in hexanes, 36 mmol) was added dropwise to a stirred solution of 5-(2,2-dibromovinyl)-1,2,3-trimethoxybenzene (3.06 g, 8.69 mmol) in dry THF (45 mL) under

argon at −48 °C. On indication of completion by TLC, the mixture was quenched with saturated ammonium chloride, extracted with dichloromethane (3 × 20 mL), the organic washings dried with $MgSO_4$, filtered and concnetrated under reduced pressure giving a crude brown oil (1.88 g, 113%). The crude oil was then filtered through a pad of silica with 4:1 pentane ether, and reduced under pressure giving a colourless solid (1.44 g, 86%). R_f 0.29 (4:1 pentane:diethyl ether), m.p. 74–75 °C, ^{1}H NMR (400.2 MHz, $CDCl_3$) δ 6.67 (s, 2H, Ar*H*), 3.79 (s, 3H, OCH_3), 3.78 (s, 6H, OCH_3), 3.00 (s, 1H, C≡C*H*), ^{13}C NMR (100.6 MHz, $CDCl_3$) δ 153.2 (s), 139.4 (s), 117.1 (s), 109.5 (s), 83.8 (s), 76.3 (s), 61.0 (s), 56.2 (s), IR ($CHCl_3$) ν_{max}/cm^{-1} 3690, 3607, 3305, 3011, 2967, 2942, 2839, 2361, 2341, 2112, 1601, 1580, 1504, 1465, 1451, 1433, 1413, 1335, 1241, 1132, 1000, 957, 924, 839. MS (+ESI) calcd. for $C_{11}H_{12}O_3$ *m/z* 192.0786 (M), found 192.0780 (M)

2.2 General procedure for preparation of the 1,4-diols ***8***

A solution of *n*-BuLi (7.6 mL of 1.6 M hexane solution, 12.2 mmol, 2.1 equiv) was added to a stirred solution of arylacetylene (12.2 mmol, 2.1 equiv) in dry THF (25 mL) under argon in Schlenk tube at −40 °C (dry ice/acetonitrile bath). Once the lithium acetylide had formed (typically 20 min), phthaldialdehyde (6.1 mmol,1 equiv) was then added as a solid and the mixture allowed to come to room temperature (typically 3 h) until TLC showed formation of the diol **8**. The reaction mixture was quenched with saturated ammonium chloride (25 mL) and extracted with ether (3 × 20 mL). The organic washings were combined, dried ($MgSO_4$), filtered, and evaporated to the crude product. Purification was achieved by flash column chromatography (3:2 pentane:diethyl ether). Generally, the *rac*/*meso* diasteriomers (1:2 to 1.9:1 mixtures) proved essentially inseparable.

1,1'-(1,2-Phenylene)bis(3-phenylprop-2-yn-1-ol) ***(8a)***

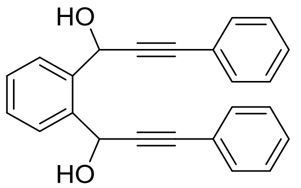

From phenylacetylene (1.3 mL, 1.2 g, 11.8 mmol), *n*BuLi (7.3 mL, 1.55 M in hexanes, 11.3 mmol) and phthaldialdehyde (0.75 g, 5.60 mmol) to yield **8a** as a pale yellow oil 1.89 g, 99% (*rac*:*meso* 1.2:1.0) with literature properties.[4] R_f 0.15 (3:2 pentane:diethyl ether). ^{1}H NMR (400.1 MHz, $CDCl_3$): δ(*rac*) 7.96-7.93 (m, 2H, Ar*H*), 7.52-7.29 (m, 12H, Ar*H*), 6.20 (d, *J* = 4.7 Hz, 2H, ArC*H*OH), 3.50 (d, *J* = 4.7 Hz, 2H, O*H*); δ(*meso*): 7.78-7.76 (m, 2H, Ar*H*), 7.52-7.29 (m, 12H, Ar*H*), 6.33 (d, *J* = 6.0 Hz, 2H, ArC*H*OH), 3.50 (d, *J* = 6.0 Hz, 2H, O*H*). ^{13}C NMR (100.6 MHz, $CDCl_3$): δ(*rac*) 138.0 (C), 131.9 (CH), 129.3 (CH), 128.8 (CH), 128.5 (CH), 128.2 (CH), 122.4 (C), 87.9 (C), 87.8 (C), 62.5 (CH); δ(*meso*) 138.4 (C), 131.9 (CH), 129.4 (CH), 128.8 (CH), 128.4 (CH), 128.2 (CH), 122.5 (C), 88.2 (C), 87.5 (C), 64.0 (CH). IR (film): ν_{max}/cm^{-1} 3691, 3587, 3008, 2928, 2856, 2361, 2230, 1601, 1491, 1455, 1444, 1375, 1242, 1070, 1031, 1015. MS (+ESI) calcd. for $C_{24}H_{18}NaO_2$ *m/z* 361.1199 (M+Na), found *m/z* 361.1191 (M+Na). The *rac*/*meso* stereochemical assignments were confirmed using the procedure of Saá;[4] which are in agreement both with the literature and across the family of **8** we have prepared.

Stereochemical enrichment of 1,1'-(1,2-phenylene)bis(3-phenylprop-2-yn-1-ol) ***(8a)***

A sample of **8a** (*rac*:*meso* 5.0:1.0) could be prepared by addition of dry tetrabutylammonium fluoride[5] (3.21 g, 12.3 mmol, 2.2 equiv) to the reaction mixture immediately after the addition of phthaldialdehyde. The reaction mixture was then subsequently warmed to room temperature over 1 h. It was quenched and worked up in the normal way to provide *meso* enriched **8a**.

No direct synthetic procedure to prepare *rac* enriched **8a** could be found. Samples of **8h** with *anti*:*syn* ~7.0:1.0 could be attained by its recrystallisation from chloroform. Conversion of the

diastereomerically enriched *anti*-**8h** sample to **8a** via Sonogashira coupling with iodobenzene (1.0 equiv) could be attained under standard conditions to provide *rac* enriched **8a**. In all cases, the diasteromers were identified with the addition of sufficient mol equivalents of Eu(facam)$_3$ to show spliting of the *rac* benzyl peak. With the exception of the structurally different **8j**, all the upfield benzyl signals correlated to the *rac* diastereomer, and the remaining peaks were assigned to each diastereomer with ^{1}H:^{1}H COSY, HMBC and HMQC NMR techniques. Representative ^{1}H NMR spectra of Eu(facam)$_3$ shifted **8a** are shown below:

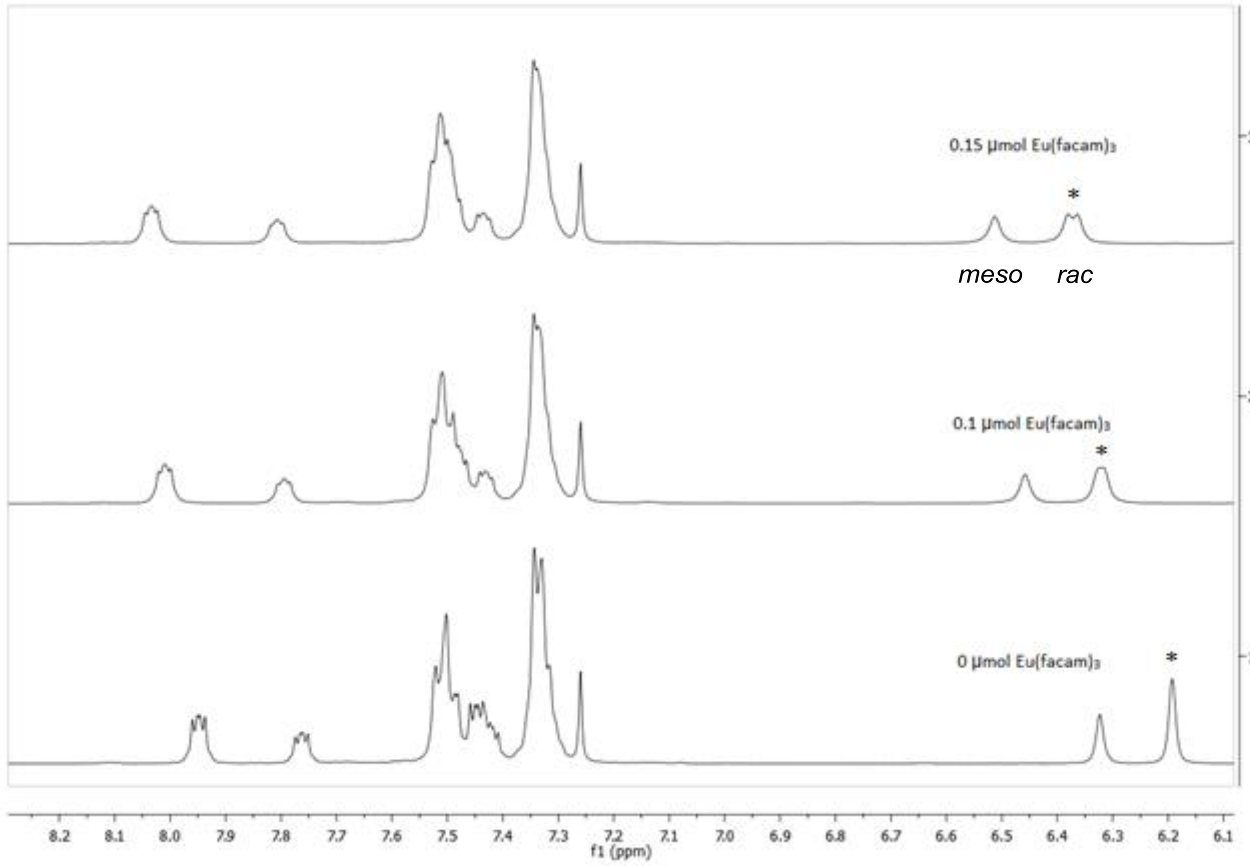

*1,1'-(1,2-phenylene)bis(3-(3,4,5-trimethoxyphenyl)prop-2-yn-1-ol) (**8b**)*

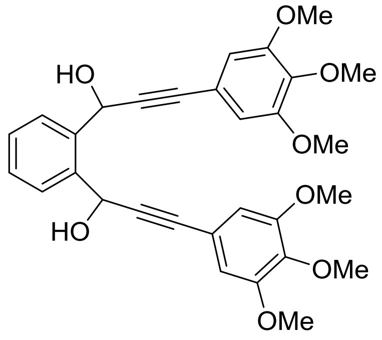

From 3,4,5-tris(methoxy)phenylacetylene (1.12 g, 5.83 mmol), *n*BuLi (3.64 mL, 1.60 M in hexanes, 4.2 mmol) and phthaldialdehyde (372 mg, 2.78 mmol) to yield novel **8b** as a colourless solid 1.13 g, 74% (*rac*:*meso* 1.9:1.0). R_f 0.14 (7:3 pentane:diethyl ether), m.p. 64-65 °C, ^{1}H NMR (400.2 MHz, CDCl$_3$) *rac* δ 7.88 (dd, *J* = 5.7, 3.5 Hz, 2H, Ar*H*), 7.39 (dd, *J* = 5.7, 3.4 Hz, 2H, Ar*H*), 6.69 (s, 4H, Ar*H*), 6.14 (s, 2H, ArC*H*OH), 3.97 (s, 2H, O*H*), 3.81 (s, 3H, OC*H*$_3$), 3.78 (s, 6H, OC*H*$_3$), *meso* δ 7.69 (dd, *J* = 5.7, 3.4 Hz, 2H, Ar*H*), 7.36 (dd, *J* = 5.7, 3.4 Hz, 2H, Ar*H*), 6.66 (s, 4H, Ar*H*), 6.27 (s, 2H, ArC*H*OH), 4.43 (s, 2H, O*H*), 3.80 (s, 3H, OC*H*$_3$), 3.75 (s, 6H, OC*H*$_3$); ^{13}C NMR (100.6 MHz, CDCl$_3$) *rac* δ 153.0 (C), 139.1 (C), 138.0 (C), 129.1 (CH), 128.1 (CH), 117.4 (C), 109.0 (CH), 87.4 (C), 87.0 (C), 62.3 (CH), 60.9 (CH3), 56.2 (CH3), ^{13}C NMR (100.6 MHz, CDCl$_3$) δ 153.0 (C), 139.0 (C), 138.5 (CH), 129.2 (CH), 129.1 (CH), 117.4 (C), 109.0

(CH), 87.4 (C), 87.2 (C), 63.8 (CH), 60.9 (CH_3), 56.1 (CH_3), IR (film): ν_{max}/cm^{-1} 3583, 3011, 2967, 2941, 2841, 2232, 1580, 1464, 1454, 1433, 1413,1242, 1187, 1166, 1132, 1059, 1032, 998, 953, 892, 837, MS (+ESI) calcd. for $C_{30}H_{30}O_8$ *m/z* 541.1833 (M+Na), found *m/z* 541.1845 (M+Na).

1,1'-(1,2-Phenylene)bis(3-(3,5-bis(trifluoromethyl)phenyl)prop-2-yn-1-ol) ***(8c)***

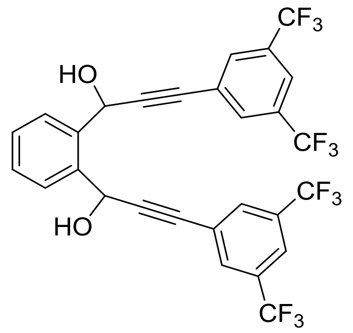

From 3,5-bis(trifluoromethyl)phenylacetylene (743 µL, 1.00 g, 4.20 mmol), *n*BuLi (2.7 mL, 1.56 M in hexanes, 4.2 mmol) and phthaldialdehyde (268 mg, 2.00 mmol) to yield novel **8c** as a colourless solid 1.03 g, 84% (*rac*:*meso* 1.9:1.0). R_f 0.14 (7:3 pentane:diethyl ether), m.p. 164-165 °C. ^{1}H NMR (400.2 MHz, MeOD): δ(*rac*) 7.97-7.83 (m, 6H, Ar*H*), 7.77 (dd, *J* = 5.8, 3.5 Hz, 2H, Ar*H*), 7.43 (dd, *J* = 5.8, 3.4 Hz, 2H, Ar*H*), 6.20 (s, 2H, ArC*H*OH); δ(*meso*) 7.97-7.83 (m, 6H, Ar*H*), 7.73 (dd, *J* = 5.8, 3.4 Hz, 2H, Ar*H*), 7.43 (dd, *J* = 5.8, 3.4 Hz, 2H, Ar*H*), 6.23 (s, 2H, ArC*H*OH); OH signals not detected due to exchange. ^{13}C NMR (100.6 MHz, MeOD): δ(*rac*) 139.5 (C), 133.1 (C, d, J_{CF} = 33.7 Hz), 132.7 (CH, q, J_{CF} = 3.0 Hz), 129.8 (CH), 128.5 (C), 126.7 (C), 124.3 (C, q, J_{CF} = 272.1 Hz), 122.8 (m, CH), 94.7 (C), 83.5 (C), 62.2 (CH); δ(*meso*) 139.8 (C), 133.1 (C, d, J_{CF} = 33.7 Hz), 131.2 (CH, q, J_{CF} = 3.1 Hz), 130.0 (CH), 129.5 (CH), 126.7 (C), 124.2 (q, J_{CF} =272.2 Hz), 122.8 (s), 94.8 (s), 84.0 (s), 63.0 (s), ^{19}F NMR (376.6 MHz, CD_3OD): δ(*rac*/*meso*) -64.69 (s), -64.72 (s). IR (film): ν_{max}/cm^{-1} 3693, 3583, 3438, 3085, 3008, 2924, 2232, 1814, 1614, 1603, 1488, 1463, 1382, 1280, 1184, 1145, 1108, 1032, 989, 951, 900, 849. MS (-ESI): calcd. for $C_{28}H_{26}O_2F_{12}$ *m/z* 609.0729 (M-H), found *m/z* 609.0714 (M-H).

Diastereomeric ratios of up to 4.8:1.0 (*rac*:*meso*) could be achieved via the addition of $CHCl_3$, as the *rac* diastereomer of **8c** is nearly insoluble in this solvent.

1,1'-(1,2-Phenylene)bis(3-(3,5-dimethoxyphenyl)prop-2-yn-1-ol) ***(8d)***

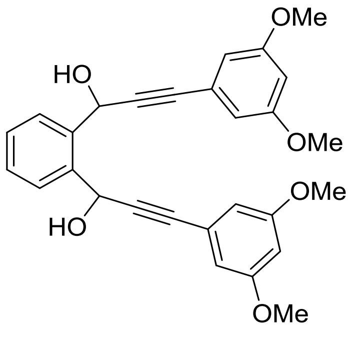

From 3,5-bis(methoxy)phenylacetylene (1.00 g, 6.17 mmol), *n*BuLi (3.9 mL, 1.68 M in hexanes, 6.55 mmol) and phthaldialdehyde (375 mg,. 2.79 mmol) to yield **8d** as an off-white solid 0.88 g, 69% (*rac*:*meso* 1.8:1.0). R_f 0.12 (7:3 pentane:diethyl ether), m.p. 73-74 °C. ^{1}H NMR (400.1 MHz, $CDCl_3$): δ(*rac*) 7.91 (dd, *J* = 5.7, 3.4 Hz, 2H, Ar*H*), 7.41 (dd, *J* = 5.7, 3.4 Hz, 2H, Ar*H*), 6.64 (d, *J* = 2.3 Hz, 4H, Ar*H*), 6.44 (t, *J* = 2.3 Hz, 2H, Ar*H*), 6.14 (s, 2H, ArC*H*OH), 3.74 (s, 12H, Me*H*); δ(*meso*) 7.71 (dd, *J* = 5.7, 3.4 Hz, 2H, Ar*H*), 7.38 (dd, *J* = 5.7, 3.4 Hz, 2H, Ar*H*), 6.62 (d, *J* = 2.3 Hz, 4H, Ar*H*), 6.43 (d, *J* = 2.3 Hz, 2H, Ar*H*), 6.29 (s, 2H, ArC*H*OH), 3.72 (s, 12H, OCH_3); OH signals not detected due to exchange. ^{13}C NMR (100.6 MHz, $CDCl_3$): δ(*rac*) 160.5 (C), 138.0 (C), 129.2 (CH), 128.1 (CH), 123.7 (C), 109.6 (CH), 102.1 (CH), 87.6 (C), 87.5 (C), 62.4 (CH), 55.5 (CH_3); δ(*meso*) 160.5 (C), 138.4 (C), 129.3 (CH), 128.1 (C), 123.7 (C), 109.6 (CH), 102.1 (CH), 87.9 (C), 87.2 (C), 63.9 (CH), 55.4 (CH_3), IR (thin film): ν_{max}/cm^{-1} 3693, 3603, 3011, 2963, 2940, 2842, 2360, 2342, 2229, 1708, 1598, 1518, 1458, 1422, 1350, 1301, 1193, 1157, 1121, 1065, 1032, 990, 952, 923, 852, 837, MS (+ESI): calcd. for $C_{28}H_{26}O_6$ *m/z* expected 481.1622 (M+Na), found *m/z* 481.1612 (M+Na).

*1,1'-(1,2-Phenylene)bis(3-(3,5-difluorophenyl)prop-2-yn-1-ol) (**8e**)*

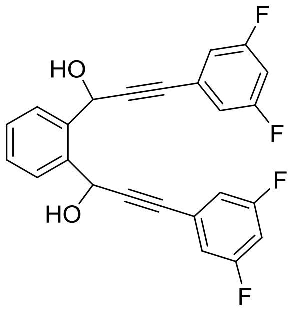

From 1-ethynyl-3,5-difluorobenzene (599 µL, 5.04mmol), *n*-BuLi (3.2 mL, 1.68 M in hexanes, 4.7 mmol) and phthaldialdehyde (322mg, 2.40mmol) to yield novel **8e** as a 774 mg, 79% (*rac*:*meso* 1.6:1.0) R_f 0.29 (6:4 pentane: ether), m.p 110-112 °C, ^{1}H NMR (400.2 MHz, $CDCl_3$) *δ* (*rac*) 7.85 (dd, *J* = 5.7, 3.4 Hz, 2H, Ar*H*), 7.44 (dd, *J* = 5.8, 3.4 Hz, 2H, Ar*H*), 7.02 – 6.91 (m, 4H, Ar*H*), 6.85 – 6.74 (m, 2H, Ar*H*), 6.10 (s, 2H, ArC*H*OH), 3.77 (s, 2H, O*H*); δ (*meso*) 7.67 (dd, *J* = 5.5, 3.4 Hz, 2H, Ar*H*), 7.41 (dd, *J* = 5.6, 3.4 Hz, 2H, Ar*H*), 7.02 – 6.91 (m, 4H, Ar*H*), 6.85 – 6.74 (m, 2H, Ar*H*), 6.21 (s, 2H, ArC*H*OH), 4.12 (s, 2H, O*H*).^{19}F NMR (376.6 MHz, $CDCl_3$) δ (*rac*) -109.21 (s); ^{19}F NMR (376.6 MHz, $CDCl_3$) δ (*meso*) -109.25 (s). ^{13}C NMR (100.6 MHz, $CDCl_3$) δ (*rac*) 161.5 (d, *J* = 13.4 Hz, C), 137.5 (C), 129.6 (CH), 128.2 (CH), 124.9 (dd, *J* = 11.7, 2.8 Hz, C), 114.8 (dd, *J* = 11.6, 2.8 Hz, CH), 105.2 (dd, *J* = 25.3, 4.4 Hz, CH), 89.7 (C), 85.5 (t, *J* = 3.9 Hz, C), 62.3 (CH); ^{13}C NMR (100.6 MHz, $CDCl_3$) δ (*meso*) 164.1 (d, *J* = 13.3 Hz, C), 138.0 (C), 129.6 (CH), 129.4 (CH), 124.9 (dd, *J* = 11.8, 2.8 Hz, C), 114.9 (dd, *J* = 26.9, 2.8 Hz, CH), 105.0 (dd, *J* = 25.3, 4.5 Hz, CH), 90.1 (C), 85.3 (t, *J* = 3.9 Hz, C), 63.8 (CH), IR (film): ν_{max}/cm^{-1} 3583, 3090, 3012, 2458, 2242, 1705, 1618, 1590, 1514, 1502, 1474, 1456, 1431, 1378, 1338, 1260, 1241, 1166,1123, 1094, 1044, 991, 954, 909, 860, MS (+ESI) calcd. for $C_{24}H_{14}O_2F_4$ *m/z* 433.0822 (M+Na), found 433.0812 (M+Na)

*1,1'-(1,2-Phenylene)bis(3-(4-(trifluoromethyl)phenyl)prop-2-yn-1-ol) (**8f**)*

From 4-(trifluoromethyl)phenylacetylene (103 µL, 1.07 g, 6.30 mmol), *n*-BuLi (4.1 mL, 1.54 M in hexanes, 6.3 mmol) and phthaldialdehyde (402 mg, 3.00 mmol) to yield **8f** as a off-white solid 1.24 g, 89% (*rac*:*meso* 1.0:1.1) with literature properties.[6] R_f 0.28 (1:1 pentane:diethyl ether), m.p. 53-54 °C. ^{1}H NMR (400.1 MHz, $CDCl_3$): δ 7.90 (dd, *J* = 5.7, 3.5 Hz, 2H, Ar*H*), 7.57 (s, 4H, Ar*H*), 7.55 (s, 4H, Ar*H*), 7.46 (dd, *J* = 5.7, 3.5 Hz, 2H, Ar*H*), 6.17 (s, 2H, ArC*H*OH), 3.39 (s, 2H, O*H*); δ(*meso*) 7.73 (dd, *J* = 5.7, 3.5 Hz, 2H, Ar*H*), 7.57 (s, 4H, Ar*H*), 7.55 (s, 4H, Ar*H*), 7.44 (dd, *J* = 5.7, 3.5 Hz, 2H, Ar*H*), 6.29 (s, 2H, ArC*H*OH), 3.77 (s, 2H, O*H*). ^{13}C NMR (100.6 MHz, $CDCl_3$): δ (*rac*) 137.7 (C), 132.1 (CH), 130.7 (C, q, J_{CF} = 32.8 Hz), 129.6 (CH), 128.5 (CH), 126.3-126.0 (m, C), 125.6-125.3 (CH, m), 123.9 (C, q, J_{CF} = 272.1 Hz), 90.1 (C), 86.5 (C), 62.6 (CH); δ(*meso*) 138.1 (C), 132.2 (CH), 130.7 (C, q, J_{CF} = 32.8 Hz), 129.6 (CH), 129.4 (CH), 126.3-126.0 (m, C), 125.6-125.3 (CH, m), 123.92 (C, q, J_{CF} = 272.1 Hz), 90.5 (C), 86.2 (C), 64.0 (CH). Multiple J_{CF} couplings prevent complete assignments being made. ^{19}F NMR (376.6 MHz, CD_3OD): δ(*rac*/*meso*) -62.92 (s), -62.93 (s). IR (thin film): ν_{max}/cm^{-1} 3690, 3586, 3009, 2960, 2929, 2873, 2359, 1922, 1700, 1615, 1516, 1488, 1455, 1405, 1374, 1324, 1265, 1172, 1134, 1107, 1068, 1018, 951, 844. MS (+ESI): calcd. for $C_{26}H_{16}O_2F_6$ *m/z* expected 497.0947(M+Na), found *m/z* 497.0942 (M+Na).

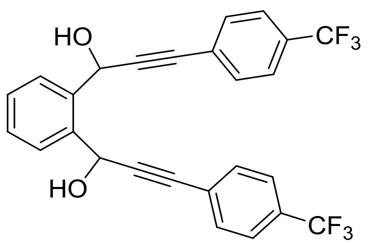

*1,1'-(1,2-Phenylene)bis(3-(4-methoxyphenyl)prop-2-yn-1-ol) (**8g**)*

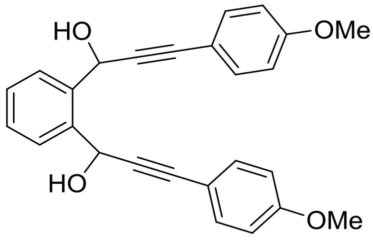

From 4-methoxyphenylacetylene (0.8 mL, 6.2 mmol), *n*-BuLi (4.1 mL, 1.54 M in hexanes, 6.3 mmol) and phthaldialdehyde (402 mg, 3.00 mmol) to yield **8g** as an off-white solid 0.98 g, 82% (*rac*:*meso* 1.3:1.0) with literature properties.[6] R_f 0.12 (1:1 pentane:diethyl ether), m.p. 48-49 °C. ^{1}H NMR (400.2 MHz, $CDCl_3$): δ(*rac*) 7.93 (dd, *J* = 5.7, 3.5 Hz, 2H, Ar*H*), 7.49-7.35 (m, 6H, Ar*H*), 6.88-6.74 (m, 4H, Ar*H*), 6.15 (d, *J* = 4.4 Hz, 2H, ArC*H*OH), 3.80 (s, 6H, Me*H*); δ(*meso*) 7.74 (dd, *J* = 5.6, 3.5 Hz, 2H, Ar*H*), 7.49-7.35 (m, 6H, Ar*H*), 6.88-6.74 (m, 4H, Ar*H*), 6.29 (d, *J* = 5.6 Hz, 2H, ArC*H*OH); 3.80 (s, 6H, OCH_3); OH signals not detected due to exchange. ^{13}C NMR (100.6 MHz, $CDCl_3$): δ(*rac*) 160.0 (C), 138.3 (C), 133.4 (CH), 129.2 (CH), 128.2 (CH), 114.5 (C), 114.1 (CH), 87.8 (C), 86.5 (C), 62.7 (CH), 55.4 (CH_3); δ(*meso*) 160.0 (C), 138.6 (C), 133.4 (CH), 129.3 (CH), 129.2 (CH), 114.6 (C), 114.1 (CH), 87.4 (C), 86.9 (C), 64.1 (CH), 55.4 (CH_3). The signal at 129.2 is formed from a *meso* CH and *rac* CH signal overlapping (as confirmed by HMBC spectral data). IR (thin film): ν_{max}/cm^{-1} 3691, 3587, 3008, 2928, 2856, 2361, 2230, 1601, 1491, 1455, 1444, 1375, 1242, 1070, 1031, 1015, 998, 950, 922, 843. MS (+ESI) calcd. for $C_{26}H_{16}O_4$ *m/z* 497.0947(M+Na), found *m/z* 497.0942 (M+Na).

*1,1'-(1,2-Phenylene)bis(3-(4-tert-butylphenyl)prop-2-yn-1-ol) (**8h**)*

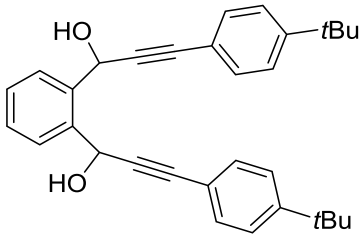

From 4-(*tert*-butyl)phenylacetylene (2.1 mL, 11.6 mmol), *n*-BuLi (6.8 mL, 1.7 M in hexanes, 11.5 mmol) and phthaldialdehyde (0.77 g, 5.74 mmol) to yield **8h** as a colourless solid 1.71 g, 66% (*rac*:*meso* 1.0:1.6) with literature properties.[7] R_f 0.11 (7:3 pentane:diethyl ether), m.p. 78-79 °C. ^{1}H NMR (400.2 MHz, $CDCl_3$): δ(*rac*) 8.00 (dd, *J* = 5.7, 3.4 Hz, 2H, Ar*H*), 7.54-7.47 (m, 4H, Ar*H*), 7.43 (dd, *J* = 5.8, 3.4 Hz, 2H, Ar*H*), 7.39-7.34 (m, 4H, Ar*H*), 6.15 (s, 2H, ArC*H*OH), 4.14 (s, 2H, O*H*), 1.36 (s, 18H, ^{t}Bu*H*); δ(*meso*) 7.77 (dd, *J* = 5.6, 3.4 Hz, 2H, Ar*H*), 7.54-7.47 (m, 4H, Ar*H*), 7.40 (dd, *J* = 5.6, 3.4 Hz, 2H, Ar*H*), 7.39-7.34 (m, 4H, Ar*H*), 6.35 (s, 2H, ArC*H*OH), 4.54 (s, 2H, O*H*), 1.35 (s, 18H, ^{t}Bu*H*). ^{13}C NMR (100.6 MHz, $CDCl_3$): δ(*rac*) 151.8 (C), 138.0 (C), 131.7 (CH), 129.3 (CH), 128.1 (CH), 125.3 (CH), 119.5 (C), 87.7 (C), 87.4 (C), 62.3 (CH), 34.8 (C), 31.2 (CH_3); δ(*meso*) 151.8 (C), 138.6 (C), 131.6 (CH), 129.1 (CH), 128.1 (CH), 125.2 (CH), 119.5 (C), 87.8 (C), 87.4 (C), 63.9 (CH), 34.8 (C), 31.2 (CH_3). IR (thin film): ν_{max}/cm^{-1} 3690, 3602, 3451, 3075, 3011, 2970, 2360, 2342, 2230, 1603, 1505, 1408, 1397, 1365, 965, 948, 837, MS (+ESI) calcd. for $C_{32}H_{34}O_2$ *m/z* 473.2451 (M+Na), found *m/z* 473.2448 (M+Na).

*1,1'-(1,2-Phenylene)bis(3-(thiophen-2-yl)prop-2-yn-1-ol) (**8i**)*

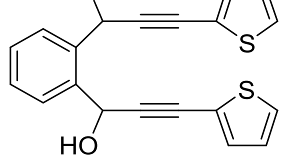

From 3-(thiophen-2-yl)acetylene (500 mg, 4.62 mmol), *n*-BuLi (2.8 mL, 1.68 M in hexanes, 4.7 mmol) and phthaldialdehyde (310 mg, 2.31 mmol) to yield novel **8i** as a low melting yellow solid 529 mg, 65% (*rac*:*meso* 1.0:1.0), R_f 0.30 (1:1 pentane:diethyl ether). ^{1}H NMR (400.2 MHz, $CDCl_3$): δ(*rac*) 7.89 (dd, *J* = 5.7, 3.4 Hz, 2H, Ar*H*), 7.46 (dd, *J* = 5.7, 3.4 Hz, 2H, Ar*H*), 7.31-7.26 (m, 4H, Ar*H*), 7.00 (dd, , *J* = 3.4, 1.7 Hz, 2H, Ar*H*), 6.20 (d, *J* = 4.8 Hz, 2H, ArC*H*OH), 2.95 (d, *J* = 4.8 Hz, 2H, O*H*); δ(*meso*) 7.73 (dd, *J* = 5.6, 3.4 Hz, 2H, Ar*H*), 7.43 (dd, *J* = 5.7, 3.4 Hz, 2H, Ar*H*), 7.31-7.26 (m, 4H, Ar*H*), 6.98 (dd, *J* = 3.4, 1.9 Hz, 2H, Ar*H*), 6.98 (d, *J* = 6.3 Hz, 2H, ArC*H*OH), 3.35 (d, *J* = 6.3 Hz, 2H, O*H*). ^{13}C NMR (100.6 MHz, $CDCl_3$): δ(*rac*) 137.5 (C),

132.7 (CH), 129.1 (CH), 128.0 (CH), 127.6 (CH), 126.9 (CH), 122.2 (C), 91.8 (C), 80.8 (C), 62.8 (CH); δ(*meso*) 138.0 (C), 132.7 (CH), 129.2 (CH), 129.2 (CH), 127.6 (CH), 127.0 (CH), 122.2 (C), 92.0 (C), 80.6 (C), 63.8 (CH). IR (thin film): ν_{max}/cm^{-1} 3691, 3676, 3583, 3438, 3113, 3079, 3011, 2878, 2360, 2340, 2224, 1801. 1669, 1603, 1559, 1540, 1519, 1487, 1454, 1425, 1408, 1373, 1269, 1191, 1109, 1081, 1046, 1004, 935, 852, 834. MS (+ESI) calcd. for $C_{20}H_{14}S_2O_2$ *m/z* 373.0319 (M+Na), found *m/z* 373.0327 (M+Na).

1-(2-(1-Hydroxyprop-2-yn-1-yl)phenyl)-3-phenylprop-2-yn-1-ol ***(8j)***

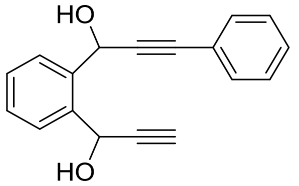

Compound **8j** was prepared in a different manner. At −78 °C under argon, *n*-BuLi (1.25 mL of 1.6 M hexane solution, 2.0 mmol) was added to a solution of trimethylsilylacetylene (280 μL, 194 mg, 1.98 mmol) in dimethoxyethane (10 mL). After 15 minutes 2-bromobenzaldehyde (231 μL, 366 mg, 1.98 mmol) was added and the mixture left to stir at −78 °C (30 min). Additional *n*-BuLi (1.25 mL of 1.6 M hexane solution, 2.0 mmol) was then added at −78 °C resulting in a colour change to bright orange/red from colourless. After 10 minutes phenylpropargylaldehyde[1] (260 mg, 2.00 mmol) was added and the reaction mixture allowed to warm slowly to room temperature over a period of 1.5 hours. The reaction was then quenched with methanol (7 mL), and stirred with aqueous potassium hydroxide solution (7 mL, 10 wt %) until complete conversion to **8j** was attained (TLC, <1 h). Final extraction with ethyl acetate (3 × 10 mL), drying ($MgSO_4$) and concentration in vacuo to provide the crude product. Purification by flash column chromatography (3:2 hexane:ethyl acetate) provided **8j** (508 mg, 98%) as a yellow oil (*syn*:*anti* 2.2:1.0), R_f 0.39 (3:2 hexane:ethyl acetate). ^{1}H NMR (400.2 MHz, $CDCl_3$): δ(*syn*) 7.71-7.66 (m, 2H, Ar*H*), 7.52-7.48 (m, 2H, Ar*H*), 7.45-7.28 (m, 5H, Ar*H*), 6.20 (s, 1H, ArC*H*OH), 6.07 (s, 1H, ArC*H*OH), 4.29 (s, 1H, O*H*), 4.18 (s, 1H, O*H*), 2.70 (d, *J* = 2.3 Hz, 1H, CC*H*); δ(*anti*) 7.96-7.83 (m, 2H, Ar*H*), 7.52-7.48 (m, 2H, Ar*H*), 7.45-7.28 (m, 5H, Ar*H*), 6.02 (s, 1H, ArC*H*OH), 5.89 (s, 1H, ArC*H*OH), 3.84 (s, 1H, O*H*), 3.77 (s, 1H, O*H*), 2.72 (d, *J* = 2.3 Hz, 1H, CC*H*). The long range acetylenic coupling on the ArC*H*OH signals were not resolved due to exchange broadening. ^{13}C NMR (100.6 MHz, $CDCl_3$): δ 138.2 (C), 137.9 (C), 137.8 (C), 137.4 (C), 131.9 (CH), 129.3 (CH), 128.8 (CH), 128.8 (CH), 128.4 (CH), 128.4 (CH), 128.2 (CH), 128.1 (CH), 122.4 (C), 122.3 (C), 87.9 (C), 87.9 (C), 87.6 (C), 87.5 (C), 82.9 (C), 82.5 (C), 76.1 (CH), 75.7 (CH), 64.0 (CH), 63.1 (CH), 62.4 (CH), 61.7 (CH). IR (thin film): ν_{max}/cm^{-1} 3294, 3066, 3036, 2891, 2232, 2220, 1957, 1886, 1634, 1599, 1572, 1490, 1454, 1443, 1412, 1329, 1280, 1202, 1177, 1098, 1070, 1018. MS (+ESI) calcd. for $C_{18}H_{14}NaO_2$ *m/z* 285.0886 (M+Na), found *m/z* 285.0887 (M+Na).

Stereochemical correlation: standard Sonogashira coupling of PhI to *anti* enriched **8j** led to *rac*-**8a** enriched samples.

3. Preparation of tetracenes and anthra[2,3-*b*]thiophene 7a–j

3.1 Optimisation of one-pot tetracene preparation

While preliminary investigations soon revealed that the xanthate esters **1c** were *not* the intimate precursors of the tetracenes **7** significant optimisation of the base, deprotonation temperature, rearrangement temperature and times were required to maximise yields of **7a**. Diol **8a** (*rac*:*meso* 1.0:1.1) was used for all these studies. Key results are summarised in Table S1.

Table S1. Details of optimisation of one-pot tetracene formation.

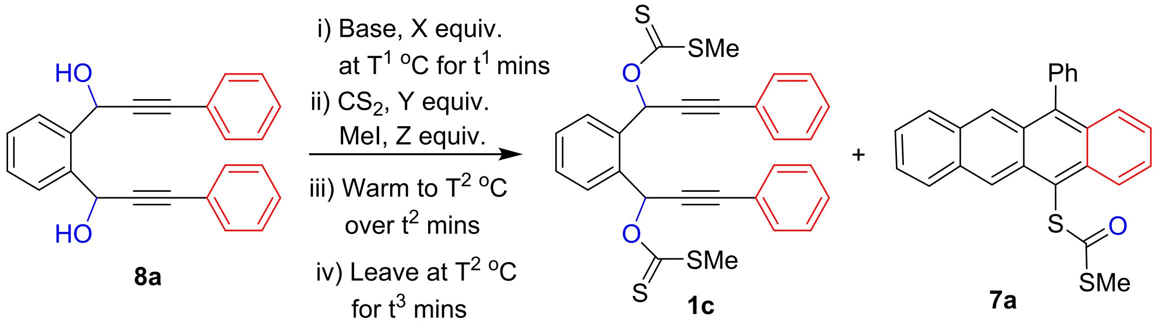

Run	Base	X	Y	Z	T^1 (°C)	t^1 (min)	T^2 (°C)	t^2 (min)	t^3 (min)	**8a:1c:7a**[a] (%)
1	NaH	2.0	3.0	8.0	-30	20	40	20	60	0:95:0
2	NaH	2.0	3.0	8.0	-50	20	40	5	60	0:45:30
3	NaH	2.0	3.0	8.0	-78	20	22	20	60	0:23:9
4	NaH	2.0	3.0	8.0	0	20	60	5	90	0:30:43
5	NEt_3	2.0	3.0	8.0	0	20	40	5	60	100:0:0
6	DMAP	2.0	3.0	8.0	0	20	40	5	60	100:0:0
7	KHMDS	2.0	3.0	8.0	0	20	60	5	90	0:9:50
8	NaHMDS	2.0	3.0	8.0	0	20	60	5	90	0:40:10
9	LiHMDS	2.0	3.0	8.0	-50	20	40	5	60	0:39:38
10	LiHMDS	2.0	3.0	8.0	0	10	60	5	90	0:30:60[b]

[a] Ratio determined by ^{1}H NMR spectroscopy on crude product. [b] See Table 1 main paper.

*3.2 General procedure for formation of tetracenes 7 and antrathiophene **7h***

Acenes **7** could be prepared via deprotonation via addition of LiHMDS (370 µL of 1.0 M tetrahydrofuran solution, 0.37 mmol) of stirred solutions of diols **8a–j** (0.18 mmol) in tetrahydrofuran (4 mL) at 0 °C under an argon atmosphere. After 20 min, CS_2 (32 µL, 0.53 mmol) and iodomethane (88 µL, 1.42 mmol) were added, and the reaction vessel transferred to a oil bath preheated to 60 °C and stirred for 90 min. This was usually accompanied by a bright orange or red colour developing within 5-15 min. The reaction was quenched with water (4 mL), extracted with dichloromethane (3 × 4 mL) and dried with $MgSO_4$. The reaction mixture could then be purified via preparative TLC (7:3 pentane: diethyl ether for **7a-j**; 6:1 pentane:CH_2Cl_2 for **7h**). The moderately soluble acenes could then be recrystallised either by liquid layering of pentane:diethyl ether and cooling from r.t. to 5 °C, or from stepwise cooling of refluxing acetonitrile solutions first to ambient temperature then to −24 °C.

*5-(Methylthiocarbonylthio)-12-phenyltetracene (**7a**)*

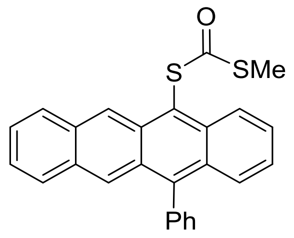

From diol **8a** (62 mg, 0.15 mmol), LiHDMS (0.35 mL, 1.0 M in THF, 0.35 mmol), CS_2 (32 μL, 40.6 mg, 0.53 mmol) and iodomethane (88 μL, 202 mg, 1.42 mmol) to yield **7a** as an orange solid 64 mg, 85% that could be recrystallised from by liquid layering (pentane:ether, 5°C). R_f 0.76 (3:2 pentane:ether), m.p. 166-167 °C. ^{1}H NMR (400.1 MHz, $CDCl_3$): δ 9.38 (s, 1H, Ar*H*), 8.72 (d, *J* = 9.0 Hz, 1H, Ar*H*), 8.32 (s, 1H, Ar*H*), 8.09 (d, *J* = 8.5 Hz, 1H, Ar*H*), 7.81 (d, *J* = 8.5 Hz, 1H, Ar*H*), 7.70-7.62 (m, 4H, Ar*H*), 7.56 (ddd, *J* = 9.0, 6.4, 1.2 Hz, 2H, Ar*H*), 7.53 (broad, s, 2H), 7.47-7.42 (m, 1H, Ar*H*), 7.39-7.34 (m, 1H, Ar*H*), 7.32 (ddd, *J* = 9.0, 6.4, 1.2 Hz, 1H, Ar*H*), 2.31 (s, 3H, SC*H*$_3$). ^{13}C NMR (100.6 MHz, $CDCl_3$): δ 190.3 (C), 142.6 (C), 138.6 (C), 135.3 (C), 132.4 (C), 132.4 (C), 131.3 (C), 131.2 (CH), 130.0 (C), 129.7 (C), 128.6 (2 overlapping signals, both CH), 128.5 (CH), 128.1 (CH), 127.9 (CH), 127.5 (CH), 127.1 (CH), 126.3 (CH), 126.3 (CH), 125.7 (CH), 125.1 (CH), 124.9 (CH), 120.2 (C), 13.7 (CH_3). UV-vis ($CHCl_3$): λ_{max} 286.8 nm. IR ($CHCl_3$): ν_{max}/cm^{-1} 3692, 3060, 3003, 2979, 2930, 2873, 2361, 2341, 1639, 1602, 1491, 1462, 1491, 1462, 1442, 1384, 1344, 1319, 1261, 1110, 1024, 909, 881, 854, 660. MS (+ESI) calcd. for $C_{26}H_{18}OS_2$ *m/z* 433.0691 (M+Na), found *m/z* 433.0702 (M+Na). Anal. calcd. for $C_{26}H_{18}OS_2$ C: 76.06, H: 4.42; found C: 76.01, H: 4.58%.

*5-(Methylthiocarbonylthio)-1,2,3-trimethoxy-12-(1,2,3-trimethoxyphenyl)tetracene (**7b**)*

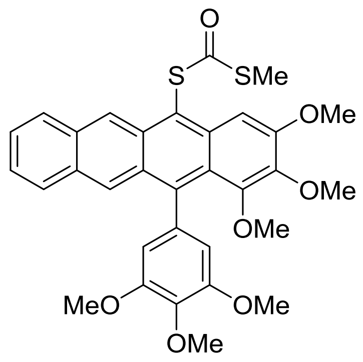

From diol **8b** (93 mg, 0.16 mmol), LiHDMS (0.35 mL, 1.0 M in THF, 0.35 mmol), CS_2 (32 μL, 40.6 mg, 0.53 mmol) and iodomethane (88 μL, 202 mg, 1.42 mmol) to yield **7a** as an orange solid 49 mg, 47% that could be recrystallised from by liquid layering (pentane:ether, 5 °C). R_f 0.14 (7:3 pentane:ether), m.p. 214-216, ^{1}H NMR (500.1 MHz, $CDCl_3$) δ 9.20 (s, 1H), 8.20 (s, 1H), 8.04 (d, *J* = 8.5 Hz, 1H), 7.82 (d, *J* = 8.5 Hz, 1H), 7.80 (s, 1H), 7.45 – 7.40 (m, 1H), 7.38 – 7.33 (m, 1H), 6.67 (s, broad, H), 4.09 (s, 3H), 4.04 (s, 3H), 3.92 (s, 3H), 3.85 (s, 6H), 3.46 (s, 3H), 2.33 (s, 3H), ^{13}C NMR (125.8 MHz, $CDCl_3$) δ 190.5 (C), 154.7 (C), 152.5 (C), 149.3 (C), 142.4 (C), 139.9 (C), 137.9 (C), 136.7 (C), 134.6 (C), 132.5 (C), 132.1 (C), 130.8 (C), 130.1 (C), 128.9 (CH), 128.4 (CH), 127.7 (CH), 126.5 (CH), 125.4 (CH), 123.9 (CH), 123.2 (C), 117.6 (C), 107.1 (CH), 99.3 (CH), 61.4 (CH_3), 61.0 (CH_3), 60.9 (CH_3), 56.3 (CH_3), 56.1 (CH_3), 13.8 (CH_3), UV-vis (CH_2Cl_2): λ_{max} 297.3 nm, IR ($CHCl_3$): ν_{max}/cm^{-1} 3690, 3059, 3045, 3006, 2966, 2938, 2838, 2422, 1717, 1635, 1618, 1601, 1584, 1544, 1525, 1509, 1464, 1421, 1414, 1379, 1350, 1313, 1297, 1254, 1164, 1129, 1105, 1053, 1006, 964, 938, 896, 880, 854, MS (+ESI) calcd. for $C_{32}H_{30}O_7S_2$ *m/z* 613.1325 (M+Na), found *m/z* 613.1313 (M+Na)

*1,3-Bis(trifluoromethyl)-12-((3,5-bis(trifluoromethyl)phenyl)-5-(methylthiocarbonylthio)tetracene (**7c**)*

From diol **8c** (109 mg, 0.18 mmol), LiHDMS (0.35 mL, 1.0 M in THF, 0.35 mmol), CS_2 (32 μL, 40.6 mg, 0.53 mmol) and iodomethane (88 μL, 202 mg, 1.42 mmol) to yield **7c** as an orange solid 60.1 mg, 56% that could be recrystallised from acetonitrile at -24 °C. R_f 0.72 (7:3 pentane:ether), m.p. 226-228 ^{o}C. ^{1}H NMR (400.1 MHz, $CDCl_3$): δ 9.45 (s, 1H, Ar*H*), 9.36 (s, 1H, Ar*H*), 8.15 (s, 1H, Ar*H*), 8.14 (d, *J* = 8.4 Hz, 1H, Ar*H*), 8.07 (s, 1H, Ar*H*), 7.98 (s, 1H, Ar*H*), 7.96 (s, 2H, Ar*H*), 7.83 (d, *J* = 8.5 Hz, 1H, Ar*H*), 7.60-7.54 (m, 1H, Ar*H*), 7.51 (m, 1H, Ar*H*), 2.41 (s, 3H, SC*H*$_3$), ^{13}C

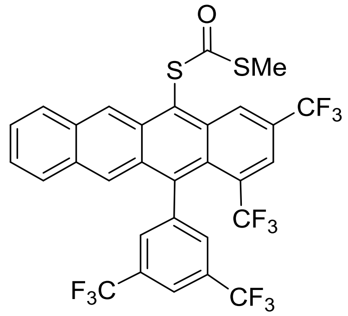

NMR (125.8 MHz, $CDCl_3$): Extensive J_{CF} couplings prevented complete assignment of the spectrum, see below ^{19}F NMR (376.5 MHz, $CDCl_3$): δ -53.5 (s, 3F), -62.8 (s, 6F), -63.6 (s, 3F). UV-vis ($CHCl_3$): λ_{max} 297.2 nm. IR ($CHCl_3$): ν_{max}/cm^{-1} 3696, 3089, 3054, 2928, 2856, 2361, 1719, 1648, 1587, 1542, 1458, 1420, 1396, 1367, 1345, 1314, 1279, 1266, 1246, 1176, 1142, 1108, 1006, 956, 902, 880, 871, 850, 640. MS (+ESI) calcd. for $C_{30}H_{14}F_{12}OS_2$ *m/z* 705.0194 (M+Na), found *m/z* 705.0187 (M+Na).

*1,3-Dimethoxy-12-(3,5-dimethoxyphenyl)-5-(methylthiocarbonylthio)tetracene (**7d**)*

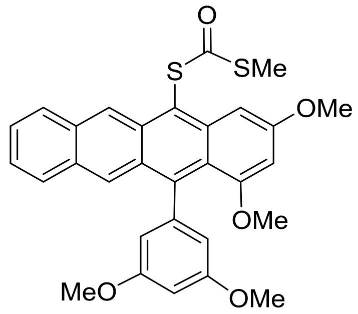

From diol **8d** (84.6 mg, 0.18 mmol), LiHDMS (0.35 mL, 1.0 M in THF, 0.35 mmol), CS_2 (32 μL, 40.6 mg, 0.53 mmol) and iodomethane (88 μL, 202 mg, 1.42 mmol) to provide a quantitative yield of **7d** as an red solid (96.3 mg) that could be recrystallised from liquid layering (2:1 pentane ether, 5°C). R_f 0.43 (7:3 pentane:ether), m.p. 220-221 °C. ^{1}H NMR (400.1 MHz, $CDCl_3$): δ 9.19 (s, 1H, Ar*H*), 8.27 (s, 1H, Ar*H*), 8.03 (d, *J* = 8.5 Hz, 1H, Ar*H*), 7.81 (d, *J* = 8.5 Hz, 1H, Ar*H*), 7.57 (d, *J* = 2.3 Hz, 1H, Ar*H*), 7.45-7.40 (m, 1H, Ar*H*), 7.35-7.30 (broad, s, 1H, Ar*H*), 6.62 (dd, *J* = 2.3, 2.2 Hz, 1H, Ar*H*), 6.32 (d, *J* = 2.2 Hz, 1H, Ar*H*), 4.02 (s, 3H, Me*H*), 3.83 (s, 6H, Me*H*), 3.51 (s, 3H, Me*H*), 2.29 (s, 3H, SC*H*$_3$). ^{13}C NMR (125.8 MHz, $CDCl_3$): 191.2 (C), 160.0 (2C, C), 159.5 (C), 158.9 (C), 144.8 (C), 141.8 (C), 138.3 (C), 132.9 (C), 132.8 (C), 130.6 (C), 129.4 (C), 129.0 (CH), 128.4 (CH), 128.3 (CH), 126.6 (CH), 125.1 (CH), 123.5 (CH), 120.8 (C), 116.7 (C), 99.2 (CH), 98.9 (CH), 94.9 (CH), 55.9 (CH_3), 55.7 (CH_3), 55.6 (CH_3), 13.8 (CH_3), UV-vis (CH_2Cl_2): λ_{max} 296.4 nm, IR ($CHCl_3$): ν_{max}/cm^{-1} 3691, 3604, 3011, 2963, 2938, 2840, 1766, 1720, 1696, 1633, 1624, 1591, 1558, 1527, 1504, 1465, 1455, 1422, 1382, 1347, 1324, 1280, 1265, 1241, 1165, 1156, 1113, 1061, 1014, 992, 969, 946, 927, 893, 880, 856, 827, MS (+ESI) calcd. for $C_{30}H_{26}O_5S_2$ *m/z* 531.1294 (M+H), found *m/z* 531.1305 (M+H). Anal. calcd. for $C_{30}H_{26}O_3S_2$ C: 67.90, H: 4.94; found C: 67.86, H: 5.01%.

*1,3-Difluoro-12-(3,5-difluorophenyl)-5-(methylthiocarbonylthio)tetracene (**7e**)*

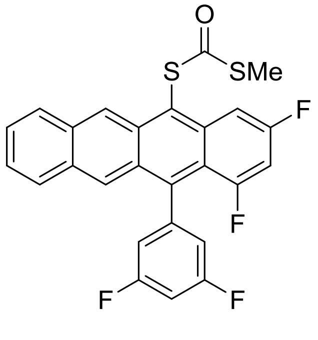

From diol **8e** (69.9 mg, 0.170 mmol), LiHDMS (0.35 mL, 1.0 M in THF, 0.35 mmol), CS_2 (31 μL, 38.9 mg, 0.51 mmol) and iodomethane (85 μL, 193 mg, 1.36 mmol) to yield **7a** as an orange solid 23.9 mg, 29% that could be recrystallised from liquid layering (2:1 pentane:ether) at 5 °C. R_f 0.67 (7:3 pentane:ether), m.p. 224-226 °C. ^{1}H NMR (500.1 MHz, $CDCl_3$) δ 9.30 (s, 1H, ArH), 8.21 (ddd, *J* = 11.2, 2.3, 1.4 Hz, 1H, ArH), 8.16 (s, 1H, ArH), 8.08 (d, *J* = 8.6 Hz, 1H, ArH), 7.83 (d, *J* = 8.6 Hz, 1H, ArH), 7.53-7.48 (m, 1H, ArH), 7.45-7.40 (m, 1H, ArH), 7.09 – 7.03 (m, 1H, ArH), 7.03 (broad, s, 2H, ArH), 6.92 (ddd, *J* = 12.4, 8.0, 2.4 Hz, 1H, ArH), 2.37 (s, 3H, MeH), ^{13}C NMR (125.8 Hz, $CDCl_3$): δ 189.0 (C), 133.3 (C), 132.8 (C), 131.8 (C), 129.3 (C), 128.7 (CH), 128.6 (CH), 127.4 (s), 127.2 (s), 126.55 (s), 125.05 (s), 13.83 (CH_3). ^{19}F NMR (376.5 MHz, $CDCl_3$) δ -98.93 (d, *J* = 9.0 Hz, 1F), -107.28 (d, *J* = 9.0 Hz, 1F), -110.00 to -110.28 (m, 2F), UV-vis (CH_2Cl_2): λ_{max} 286.3 nm, IR ($CHCl_3$): ν_{max}/cm^{-1} 3691, 3606, 3045, 3009, 2933, 2419, 1720, 1648, 1622, 1594, 1600, 1459, 1432, 1386, 1370, 1348, 1320, 1283, 1256, 1193, 1150, 1133, 1121, 1074, 1004, 989, 942, 881, 857, 831, MS (+ESI) calcd. for $C_{26}H_{14}OS_2F_4$ m/z 482.0422 (M), found m/z 482.0434 (M).

*5-(Methylthiocarbonylthio)-2-(trifluoromethyl)-12-(4-(trifluoromethyl)phenyl)tetracene (**7f**)*

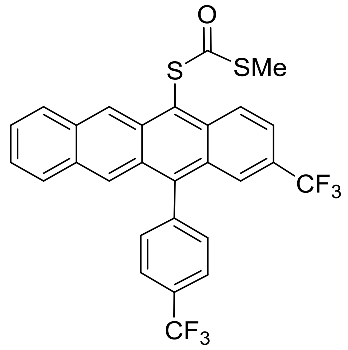

From diol **8f** (167 mg, 0.36 mmol), LiHDMS (0.71 mL, 1.0 M in THF, 0.71 mmol), CS_2 (64 µL, 81.2 mg, 1.07 mmol) and iodomethane (177 µL, 404 mg, 2.84 mmol) to yield **7f** as an orange solid 86.0 mg, 44% that could be recrystallised from liquid layering (pentane:ether) 5 ^{o}C. R_f 0.72 (3:2 pentane:ether), m.p. 225-226 °C. ^{1}H NMR (500.1 MHz, $CDCl_3$): δ 9.42 (s, 1H, Ar*H*), 8.84 (d, *J* = 9.4 Hz, 1H, Ar*H*), 8.23 (s, 1H, Ar*H*), 8.12 (d, *J* = 8.6 Hz, 1H, Ar*H*), 7.98 (d, *J* = 8.2 Hz, 2H, Ar*H*), 7.91 (s, 1H, Ar*H*), 7.89 (d, *J* = 8.5 Hz, 1H, Ar*H*), 7.68 (dd, *J* = 9.4, 1.7 Hz, 1H, Ar*H*), 7.66 (broad, s, 2H, Ar*H*), 7.57-7.48 (m, 1H, Ar*H*), 7.48-7.39 (m, 1H, Ar*H*), 2.36 (s, 3H, SC*H*$_3$). ^{13}C NMR (125.8 MHz, $CDCl_3$): δ 189.3 (C), 142.6 (C), 141.5 (C), 135.0 (C), 133.2 (C), 133.0 (C), 132.0 (C), 131.6 (CH), 131.1 (C, q, J_{CF} = 32.7 Hz), 129.7 (C), 128. 7 (CH), 128.6 (CH), 128.4 (CH), 128.1 (C), 127.4 (C, q, J_{CF} = 32.3 Hz), 127.3 (CH), 127.1 (CH), 126.7 (CH), 126.1 (CH, dd, J_{CF} = 7.2, 3.6 Hz), 125.6 (CH), 125.5 (CH, q, J_{CF} = 5.1 Hz), 125.3 (C, q, J_{CF} = 27.3 Hz), 123.1 (C, q, J_{CF} = 27.0 Hz), 122.4 (CH, q, J_{CF} = 2.7 Hz), 122.4 (C), 13.8 (CH_3), ^{19}F NMR (376.6 MHz, $CDCl_3$): δ -62.4 (s), -63.3 (s). UV-vis (CH_2Cl_2): λ_{max} 290.9 nm. IR ($CHCl_3$): ν_{max}/cm^{-1} 2928, 2856, 1721, 1645, 1456, 1406, 1386, 1363, 1347, 1325, 1300, 1278, 1263, 1168, 1131, 1108, 1068, 1020, 975, 882, 847, 821, 638, 609. MS (+ESI) calcd. for $C_{30}H_{16}OS_2F_6$ *m/z* 547.0620 (M+H), found *m/z* 547.0625 (M+H).

*2-Methoxy-12-(4-methoxyphenyl)-5-(methylthiocarbonylthio)tetracene (**7g**)*

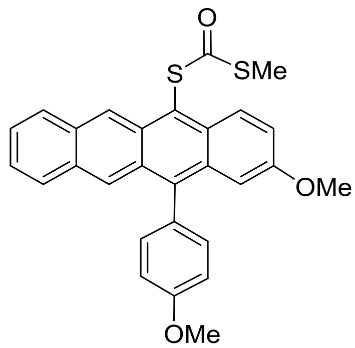

From diol **8g** (70.8 mg, 0.18 mmol), LiHDMS (0.35 mL, 1.0 M in THF, 0.35 mmol), CS_2 (32 µL, 40.6 mg, 0.53 mmol) and iodomethane (88 µL, 202 mg, 1.42 mmol) to yield **7g** as an orange solid 32 mg, 38% that could be recrystallised from hot acetonitrile subsequently chilled to -28 °C. R_f 0.41 (7:3 pentane:ether). m.p. 176-178 °C ^{1}H NMR (500.1 MHz, $CDCl_3$): δ 9.32 (s, 1H, Ar*H*), 8.64 (d, *J* = 9.6 Hz, 1H, Ar*H*), 8.28 (s, 1H, Ar*H*), 8.06 (d, *J* = 8.5 Hz, 1H, Ar*H*), 7.81 (d, *J* = 8.5 Hz, 1H, Ar*H*), 7.44 (s, broad, 2H, Ar*H*), 7.43-7.39 (m, 2H, Ar*H*), 7.37-7.33 (m, 2H, Ar*H*), 7.28 (d, *J* = 2.5 Hz, 1H, Ar*H*), 7.20 (d, *J* = 8.8 Hz, 2H, Ar*H*), 6.87 (d, *J* = 2.5 Hz, 1H, Ar*H*), 4.01 (s, 3H, OC*H*$_3$), 3.73 (s, 3H, OC*H*$_3$), 2.30 (s, 3H, SC*H*$_3$); ^{13}C NMR (125.8 MHz, $CDCl_3$): δ 190.6 (C), 159.4 (C), 156.6 (C), 139.8 (C), 132.7 (C), 132.3 (CH), 131.9 (C), 131.5 (C), 131.4 (C), 131.4 (C), 131.1 (C), 130.6 (C), 128.7 (CH), 128.5 (CH), 128.3 (CH), 126.4 (CH), 125.9 (CH), 125.7 (CH), 125.0 (CH), 123.2 (CH), 120.2 (C), 114.3 (CH), 103.0 (CH), 55.6 (CH_3), 55.3 (CH_3), 13.8 (CH_3). UV-vis ($CHCl_3$): λ_{max} 290.0 nm. IR ($CHCl_3$): ν_{max}/cm^{-1} 3607, 3489, 3045, 2928, 2854, 2359, 2338, 1718, 1648, 1602, 1506, 1457, 1396, 1368, 1345, 1314, 1279, 1176, 1142, 1107, 1106, 956, 902, 880, 871, 850. MS (+ESI) calcd. for $C_{30}H_{22}O_3S_2$ *m/z* 471.1083 (M+H), found *m/z* 471.1100 (M+H).

*2-(tert-Butyl)-12-(4-tert-butylphenyl)-5-(methylthiocarbonylthio)tetracene (**7h**)*

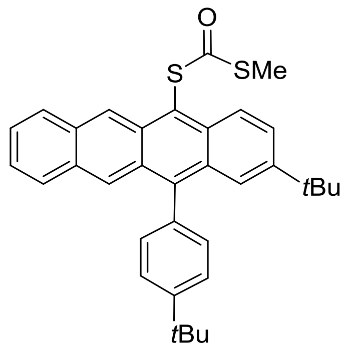

From diol **8h** (80 mg, 0.18 mmol), LiHDMS (0.35 mL, 1.0 M in THF, 0.35 mmol), CS_2 (32 µL, 40.6 mg, 0.53 mmol) and iodomethane (88 µL, 202 mg, 1.42 mmol) to yield **7h** as an orange solid 20.3 mg, 22% that could be recrystallised by liquid layering (pentane:ether). R_f 0.58 (7:3 pentane:ether), m.p. 255-256 °C. ^{1}H NMR (500.1 MHz, $CDCl_3$): δ 9.33 (s, 1H), 8.64 (d, *J* = 9.4 Hz, 1H), 8.39 (s, 1H), 8.07 (d, *J* = 8.6 Hz, 1H), 7.84 (d, *J* = 8.6 Hz, 1H), 7.69-7.64 (m, 3H), 7.56 (d, *J* = 1.6 Hz, 1H), 7.45 (s, broad 2H), 7.44-7.40 (m, 1H), 7.37-7.33 (m, 1H), 2.31 (s, 3H, SCH_3), 1.52 (s, 9H, ^{t}Bu*H*), 1.28 (s, 9H, ^{t}Bu*H*).^{13}C NMR (125.8 MHz, $CDCl_3$): δ 190.8 (C), 151.0 (C), 147.1 (C), 142.6 (C), 135.6 (C), 134.4 (C), 132.3 (C), 132.2 (C), 131.2 (CH), 131.0 (C), 130.4 (C), 130.1 (C), 128.7 (CH), 128.6 (CH), 127.4 (CH), 127.2 (CH), 126.2 (CH), 126.0 (CH), 125.5 (CH), 125.4 (CH), 124.7 (CH), 122.1 (CH), 119.2 (C), 35.0 (C), 35.0 (C), 31.7 (CH_3), 30.6 (CH_3), 13.8 (CH_3). UV-vis (CH_2Cl_2): λ_{max} 289.0 nm. IR ($CHCl_3$): ν_{max}/cm^{-1} 3092, 3871, 3853, 3838, 3821, 3802, 3691, 3676, 3649, 3608, 3080, 3057, 2966, 2931, 2870, 2377, 2337, 2229, 1734, 1699, 1684, 1636, 1602, 1559, 1541, 1490, 1457, 1396, 1365, 1341, 1314, 1263, 1248, 1190, 1128, 1107, 1019, 998, 979, 956, 923, 881, 852. MS (+ESI) calcd. for $C_{34}H_{34}OS_2$ *m/z* 545.1943 (M+Na), found *m/z* 545.1968 (M+Na).

*11-(Methylthiocarbonylthio)-4-(thiophen-2-yl)anthra[2,3-b]thiophene (**7i**)*

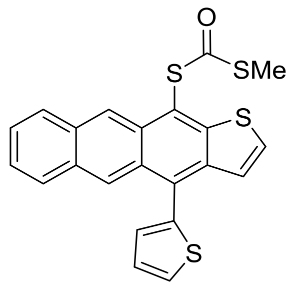

From diol **8i** (82.9 mg, 0.24 mmol), LiHDMS (0.35 mL, 1.0 M in THF, 0.35 mmol), CS_2 (43 µL, 54.1 mg, 0.71 mmol) and iodomethane (88 µL, 298 mg, 1.89 mmol) to yield **7i** as a yellow-orange solid 38.3 mg, 38% that could be recrystallised from liquid layering (2:1 pentane:ether) at 5°C. R_f 0.65 (7:3 pentane:ether), m.p. 209-210 °C. ^{1}H NMR (400.1 MHz, $CDCl_3$): δ 9.06 (s, 1H), 8.69 (s, 1H), 8.10 (d, *J* = 8.4 Hz, 1H), 7.93 (d, *J* = 8.4 Hz, 1H), 7.66 (dd, *J* = 4.1, 2.2 Hz, 1H), 7.53-7.47 (m, 1H), 7.47-7.42 (m, 1H), 7.44 (d, *J* = 5.8 Hz, 1H), 7.36 (s, 1H), 7.35 (d, *J* = 2.0 Hz, 1H), 7.33 (d, *J* = 5.8 Hz, 1H), 2.35 (s, 3H, SCH_3). ^{13}C NMR (125.8 MHz, $CDCl_3$): δ 188.8 (C), 149.0 (C), 138.9 (C), 138.3 (C), 132.1 (C), 131.3 (C), 130.8 (C), 130.5 (C), 130.2 (C), 129.6 (CH), 129.3 (CH), 128.5 (CH), 128.5 (CH), 127.5 (CH), 127.2 (CH), 126.6 (CH), 126.4 (CH), 125.8 (CH), 124.8 (CH), 123.0 (CH), 116.6 (C), 13.8 (CH_3). UV-vis (CH_2Cl_2): λ_{max} 283.7 nm. IR ($CHCl_3$): ν_{max}/cm^{-1} 3692, 3058, 3007, 2692, 2932, 1719, 1648, 1502, 1460, 1432, 1413, 1369, 1321, 1290, 1262, 1176, 1115, 1091, 1045, 1028, 973, 909, 882, 853, 821. MS (+ESI) calcd. for $C_{22}H_{14}OS_4$ *m/z* 444.9820 (M+Na), found *m/z* 444.9811 (M+Na). Anal. calcd. for $C_{22}H_{14}OS_4$ C: 62.53, H: 3.34%, found C: 62.67, H: 3.44%.

*5-(Methylthiocarbonylthio)tetracene (**7j**)*

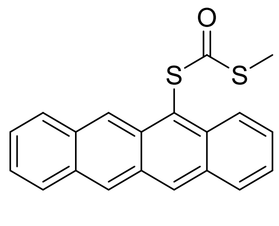

To a solution of **8j** (118 mg, 0.450 mmol) in dimethoxyethane (5 mL) at 0 °C under argon, lithium bis(trimethylsilyl)amide (0.95 mL of 1.0 M dimethoxyethane solution, 0.95 mmol) was added. After 10 minutes at 0 ^{o}C carbon disulfide (81 µL, 1.35 mmol) then methyl iodide (224 µL, 3.60 mmol) were added at the same temperature. The reaction mixture was immediately placed in a preheated 80 °C oil bath. The reaction was left at 80 °C (1 h) before prompt cooled to room temperature. The reaction mixture was extracted with

dichloromethane (3 × 10 mL) and washed with water (10 mL). The combined organic extracts were dried ($MgSO_4$) passed through a plug of silica and concentrated *in vacuo* to provide the crude product. Purification was achieved by dissolution in warm diethyl ether (1.5 mL) followed by cooling (-24 °C, 2 h) to provide pure **7j** (75.5 mg, 50%) as a red solid. R_f (6:1 pentane:dichloromethane) 0.25, m.p. 200-203 °C. ^{1}H NMR (500.1 MHz, $CDCl_3$): δ 9.29 (s, 1H, Ar*H*), 8.91 (s, 1H, Ar*H*), 8.74 (s, 1H, Ar*H*), 8.68-8.61 (m, 1H, Ar*H*), 8.14-8.00 (m, 3H, Ar*H*), 7.61-7.55 (m, 1H, Ar*H*), 7.50-7.42 (m, 3H, Ar*H*), 2.26 (s, 3H, SMe); ^{13}C NMR (125.8 MHz, $CDCl_3$): δ 190.6 (C), 135.8 (C), 133.0 (C), 132.8 (C), 132.4 (CH), 131.7 (C), 131.6 (C), 130.3 (C), 129.4 (CH), 129.1 (CH), 128.1 (CH), 128.0 (CH), 127.8 (CH), 126.3 (CH), 126.2 (CH), 126.0 (CH), 125.4 (CH), 124.9 (CH), 120.1 (C), 13.8 (CH_3). UV-vis (CH_2Cl_2): λ_{max} 283.0 nm. IR (thin film): ν_{max}/cm^{-1} 3414, 2929, 2250, 2109, 1787, 1723, 1658, 1631, 1611, 1551, 1530, 1513, 1502, 1481, 1462, 1443, 1382, 1289, 1262. MS (+ESI) calcd. for $C_{20}H_{14}NaOS_2$ *m/z* 357.0378 (M+Na), found *m/z* 357.0383 (M+Na).

3.3 By-products and control reactions

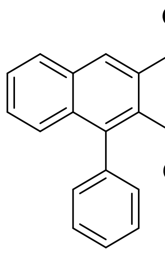

12 R = H, Me

Co-product formation in the preparation of **7a** (at a 1.78 mmol scale) was investigated. Chromatography of the crude product run under argon atmosphere also afforded trace amounts of a red solid tentatively assigned as a mixture of 12-methoxy-6-phenyltetracen-5-ol and 6-phenyltetracene-5,12-diol **12** 40.1 mg (~6% yield), R_f (0.82, 4:1 pentane:CH_2Cl_2), based on its ^{1}H NMR spectroscopy and EI+. Further data could not be attained due to the compound's rapid degradation even in dry degassed solvents. ^{1}H NMR (400.1 MHz, $CDCl_3$): δ 9.71 (s), 9.23 (s), 9.06 (d, *J* = 9.1 Hz), 8.96 – 8.90 (m), 8.72 (d, *J* = 13.9 Hz), 8.54 (d, *J* = 9.1 Hz), 8.43 (s), 8.36 (s), 8.29 (s), 8.24 (s), 8.17 – 8.08 (m), 8.02 (dd, *J* = 16.9, 8.4 Hz), 7.95 (dd, *J* = 6.2, 3.3 Hz), 7.89-7.78 (m), 7.74 (dd, *J* = 8.7, 7.0 Hz), 7.71-7.28 (m), 7.14 (dd, *J* = 6.8, 3.1 Hz), 2.60-2.51 (m). MS (+EI) calcd. for $C_{24}H_{16}OS_2$ *m/z* 336.1145 (M), found *m/z* 336.1108 (M); MS (+EI) calcd. for $C_{25}H_{18}OS_2$ *m/z* 350.1301 (M), found *m/z* 350.1289 (M).

Continued elution afforded first **7a** (113 mg, 15% yield), data as above, followed by a red compound (44.6 mg) that could not be fully characterised: ^{1}H NMR (400.1 MHz, $CDCl_3$): δ 7.88-7.24 (m), 2.63 (s), 2.53 (s), 2.49 (s), 2.44 (s), 2.43 (s), 2.39 (s), 2.37 (s), 2.33 (s), 2.32 (s), 2.30 (s), 2.27 (s), 2.24 (s), 2.24 (s), 2.21 (s), 2.17 (s), 2.13 (s), 2.08 (s), 2.06 (s), 2.04 (s), 2.04 (s), 1.96 (s), 1.89 (s), 1.77 (s), 1.70 (s). Because of these issues allene **14** was prepared as a model compound.

*1,3-Diphenyl-5-(methylthiocarbonylthio)propa-1,2-diene (**14**) and (3,4-di((Z)-benzylidene)-(1,2-(methylthiocarbonylthio)cyclobutane-1,2-diyl)dibenzene (**15**)*

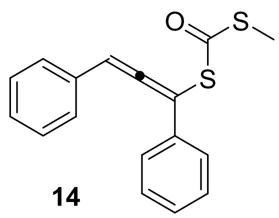

14

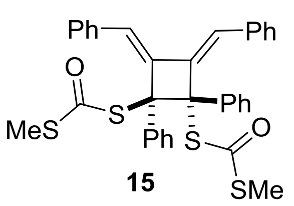

15

Alcohol **13** (see Scheme 4 main paper, 1.50 g, 7.21 mmol) was dissolved in tetrahydrofuran (30 mL) and cooled to −78 °C under argon. The alcohol was then treated with sodium hydride (433 mg, 10.8 mmol, 60% in oil), followed by addition of carbon disulfide (0.65 mL, 10.8 mmol). After 15 minutes methyl iodide (2.15 mL, 28.8 mmol) was added to the solution and mixture stirred for a further 15 minutes at -78 °C. The reaction mixture was allowed to come to room while stirring (3 h). The reaction mixture was then quenched with water until gas evolution stopped and promptly extracted

with diethyl ether (2 × 30 mL). The combined organic layers were washed rapidly with brine (2 × 30 mL), dried ($MgSO_4$) and concentrated to provide crude allene **14** (2.15 g, 7.21 mmol, >99%) as an orange oil. R_f (3:2 pentane:diethyl ether) 0.25 whose data were attained immediately at room temperature. ^{1}H NMR (400.2 MHz, $CDCl_3$): δ 7.63 (m, 2H, Ar*H*), 7.49-7.44 (m, 2H, Ar*H*), 7.42-7.27 (m, 6H, Ar*H*), 6.73 (s, 1H, CC*H*Ar), 2.43 (s, 3H, SC*H*3); ^{13}C NMR (100.6 MHz, $CDCl_3$) δ 212.2 (C), 188.5 (C), 133.9 (C), 131.9 (C), 129.1 (CH), 128.8 (CH), 128.4 (CH), 128.3 (CH), 128.1 (CH), 126.7 (CH), 100.3 (C), 97.8 (CH), 13.7 (CH_3); IR (film): ν_{max}/cm^{-1} 3060, 3029, 2927, 2856, 1713, 1645, 1597, 1491, 1446, 1311, 1201, 1150, 1073, 1054, 1029. MS (+ESI) calcd. for $C_{17}H_{14}NaOS_2$ *m/z* 321.0369 (M+Na), found *m/z* 321.0378 (M+Na). On standing **14** rapidly oligomerised giving broad ^{1}H NMR spectra and mass spectra showing signals a (**14**)$_n$ (n>1) due to its rapid decomposition in solution it could only be characterised in the solid state. In one crystal of cyclobutane **15** were isolated serendipitously and characterised by X-ray crystallography (space group P2yn; *a* = 11.293, *b* = 14.124, *c* = 18.890 Å; *α* = 103.6 ^{o}). The compound is highly reactive in solution and all attempts at solution NMR studies failed.

4. Characterisation of electro-optic properties of acenes 7

4.1 Cyclic voltammetry studies

Cyclic voltammetry measurements were carried out in a three electrode cell under an argon atmosphere using 0.30 M $TBAPF_6$ in dry CH_2Cl_2 as the supporting electrolyte. Typical concentrations of the tetracene analytes **7** ranged from 2 mM to 5 mM. Cyclic voltammagrams were recorded using a CH instruments CHI700D potentiostat. Curves were referenced from the Ag reference electrode calibrated using the ferrocene/ferrocinium (Fc/Fc^+) redox couple as an internal standard, the halfwave potential ($E_{½}$) of which was found to be 0.44-0.47 V relative to the reference electrode. A representative trace is shown in Chart S1. Estimates of the HOMO levels could be attained by taking the onset of the oxidation peak and the known HOMO of Ferrocene (−4.4 eV) using the formula HOMO= $-[E^{Onset}_{Ox} + 4.4]$eV.[8] These values are presented in Table S2. Estimation of the LUMO values in **7** were prevented by unpredictable competing reduction processes (we believe are associated with the xanthate function).

Chart S1. Representative CV of **7a** (2mM vs (Fc/Fc^+) redox couple, 0.3 M $TBAPF_6$ in CH_2Cl_2).

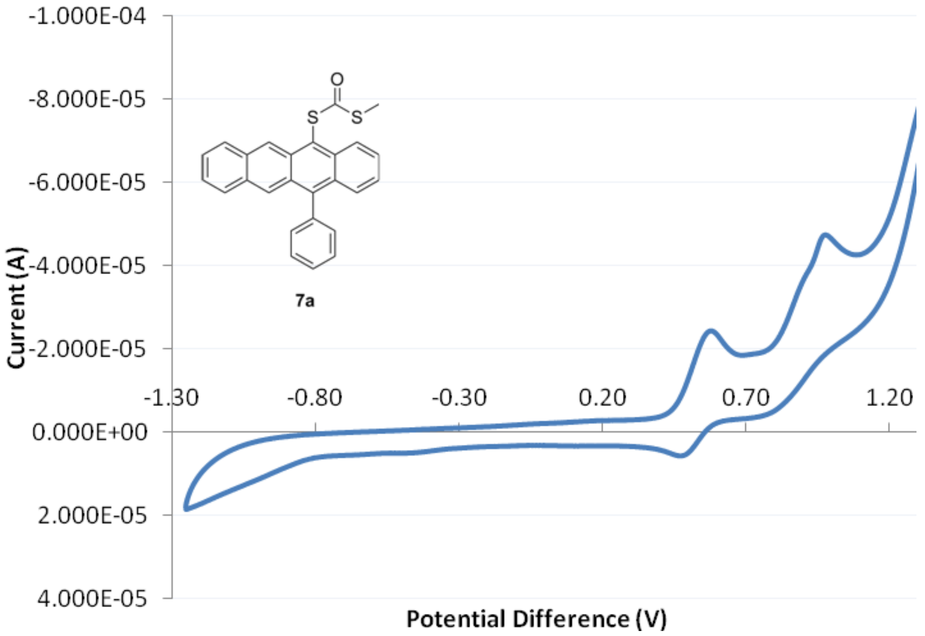

Table S2. Table of estimated HOMO levels from CV measurements.

Structure	$E_{½}^{Ox}$ (V)	$E_{½}^{Ox}$ (V) (relative to Fc/Fc^{+})	HOMO estimate (eV)
**7a**	+0.97	+0.52	-4.82
**7b**	+0.68	+0.22	-4.62
**7c**	+1.52	+1.07	-5.14
**7d**	+0.74	+0.29	-4.65
**7e**	+1.10	+0.65	-5.04
**7f**	+1.21	+0.76	-5.46
**7g**	+0.88	+0.43	-4.70
**7h**	+0.99	+0.55	-4.79
**7i**	+1.05	+0.60	-4.88
**7j**	+1.09	+0.62	-4.82

4.2 Optical studies

Estimates of the optical bandgap E_g opt. were attained form the onset of the lowest energy band in the visible spectrum. The method of Tauc was used: plots of $(ah\nu)^{1/2}$ as a function of hν from the primary A vs. wavelength (converted into eV).[9]

4.3 DFT calculations

We utilised the B3LYP hybrid functional[10] in conjunction with the 6-31G(d,p) basis set for all *in silico* studies. Calculations were carried out using Gaussian 09 Rev.D.01 software on the NSCCS Columbus and Slater servers.[11]

5. Crystallographic studies of acenes 7 and cyclobutane 15

Crystallographic data on compounds **7b**,**c**, **7e**,**f** and **15** were collected using the University of Nottingham, School of Chemistry Crystallography Service. Compounds **7a** and **7h** were collected using the DIAMOND[12] facility. Details of the structures are available through their CIF information available through the Cambridge Crystallographic Information Service for files (CCDC 1027105-1027111).[13] Aside from the long C-C bond shown in **17** all of the intramolecular distances and angles were in the expected range so discussions here focus mainly on intermolecular packing.

*5-(Methylthiocarbonylthio)-12-phenyltetracene (**7a**)*

In the lattice of **7a** four units are found in close association, with local C_2 symmetry, two linked by slip stack $C_\pi \ldots C_\pi$ interactions (see Scheme 5A, main paper) and two by SMe…C_π contacts (Figure S2a). Columnar features of these repeat units are in filled, inclined, molecules of **7a** (Figure S2b).

Figure S2. (a) Association of **7a** into tetramer cores. (b) Packing of tetramer cores and infilling.

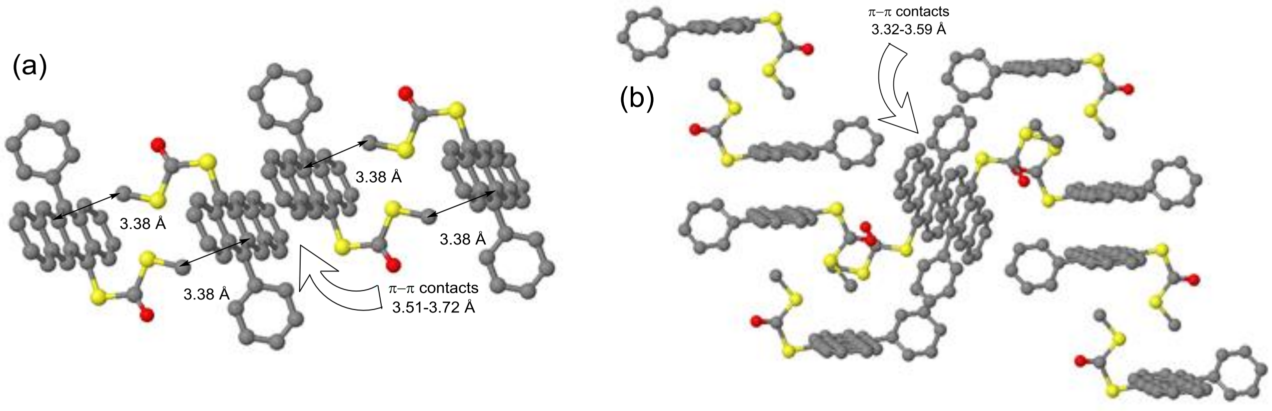

*1,3-Dimethoxy-12-(3,5-dimethoxyphenyl)-5-(((methylthio)carbonyl)thio)tetracene (**7d**)*

Intermolecularly two **7d** molecules combine, in a C_2 unit, *via* CH..O contacts (2.39 Å) which also promotes a close OMe arrangement (3.76 Å) (Figure S3a). Duplicates of this unit overlap, the electron rich dimethoxy aryl ring engaging in face-to-face $C_\pi \ldots C_\pi$ stacking with the unsubstituted aryl in the adjacent unit (Scheme 5**C** main paper and Figure S3b). Perpendicular to this arrangement the brickwork stacked columns propagate through the lattice by xanthate-to-π-aryl contacts (Figure S3c).

Figure S3. (a) Dimers of **7d**. (b) Brickwork face-to-face $C_{\pi}\ldots C_{\pi}$ stacking. (c) Association through xanthate contacts.

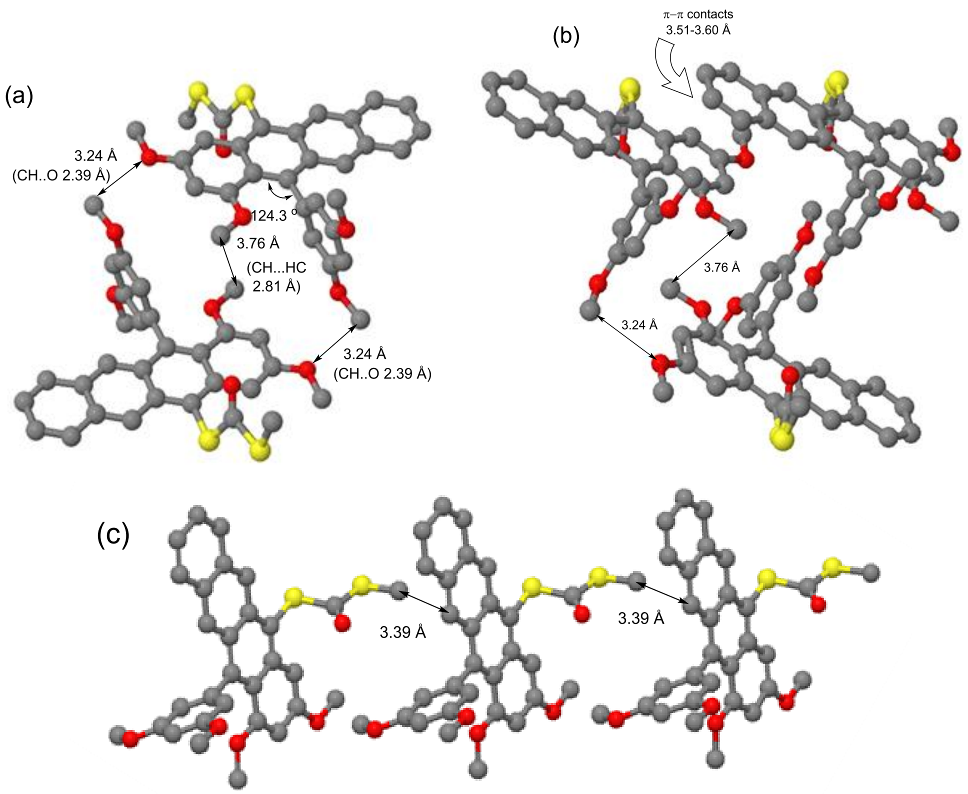

*5-(Methylthiocarbonylthio)-2-(trifluoromethyl)-12-(4-(trifluoromethyl)phenyl)tetracene (**7f**)*

Intermolecular packing of **7f** is dominated by a common step stacking motif in arrays of **7f** is engendered by $C_{\pi}\ldots C_{\pi}$ interactions (see Scheme 5**D**, main paper) and $F\ldots C_{\pi}$ contacts (Figure S4a). This arrangement is duplicated through cross-linking through loose associations of the 4-CF_3Ph groups (Figure S4b) leading duplication of the step stack pattern. Finally, associations of the xanthate groups to neighbouring aryls lead to a final step stack motif approximately perpendicular to these features (Figure S4c).

Figure S4. (a) Step stacking of **7f**. (b) Pairing though 4-CF_3Ph association. (c) Association through xanthate contacts.

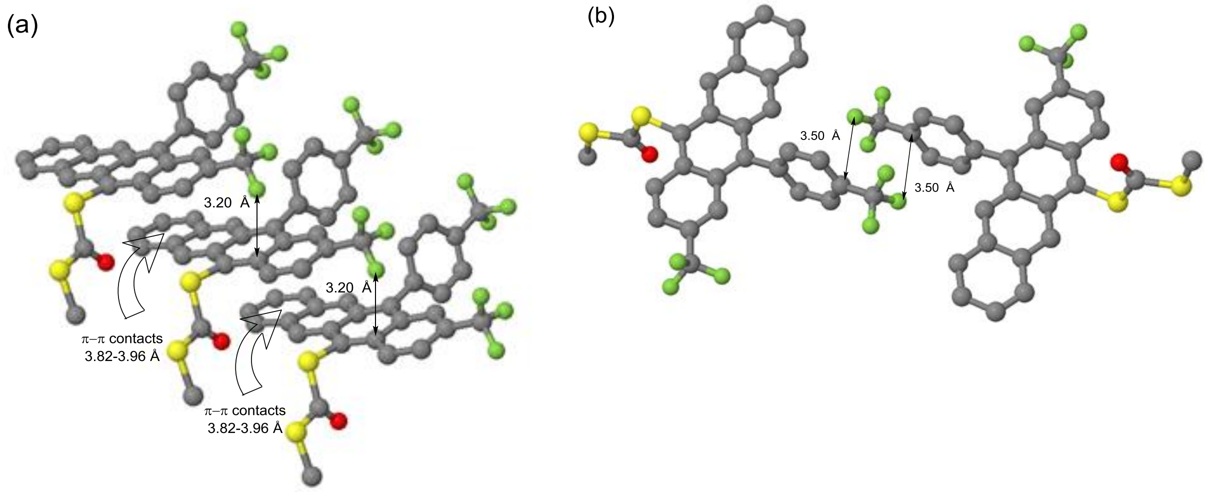

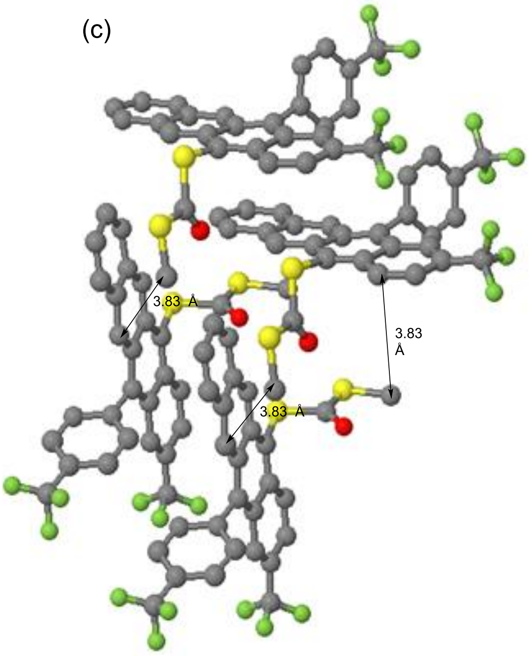

*2-Methoxy-12-(4-methoxyphenyl)-5-(methylthiocarbonylthio)tetracene (**7g**)*

Two molecules of **7g** are associated through slipped $C_\pi \ldots C_\pi$ interactions (Figure S4a). This is shown in Scheme 5**E** in the main paper. The dimers of Figure S5a are associated together by $CH \ldots C_\pi$ interactions (Figure S5b) leading to a pseudo 1D columnar arrangement.

Figure S5. (a) Slipped $C_\pi \ldots C_\pi$ interactions in **7g**. (b) Stacking of dimers through $CH \ldots C_\pi$ interactions.

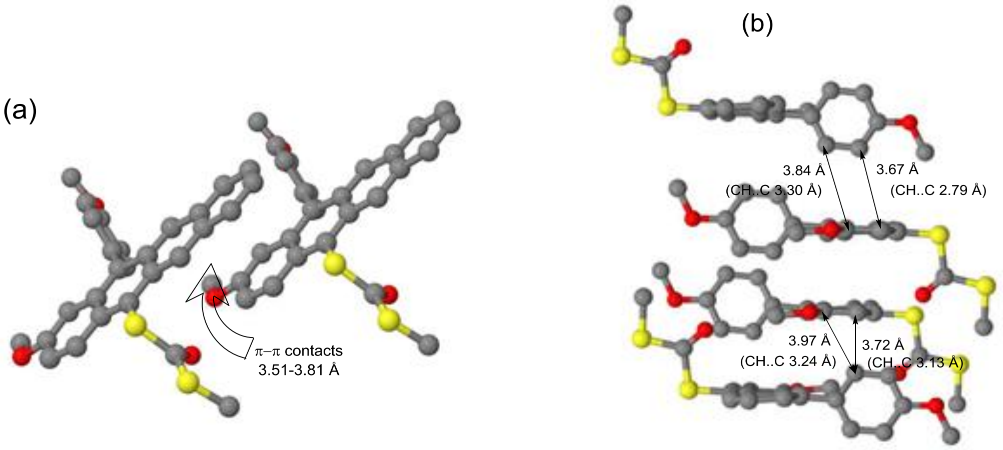

*2-tert-Butyl-12-(4-tert-butylphenyl)-5-(methylthiocarbonylthio)tetracene (**7h**)*

Two units of **7h** are associated, with local C_2 symmetry, by slip stack $C_\pi \ldots C_\pi$ interactions (see Scheme 5A, main paper and Figure S6a). These dimers are further associated through SMe…C_π contacts (Figure S6b). Parallel columns of these assemblies are paired through loose packing of the 4-*t*BuPh units (Figure S6c).

Figure S6. (a) Dimers of **7h** into tetramer cores. (b) Association through xanthate contacts. (c) Pairing though 4-*t*-BuPh association.

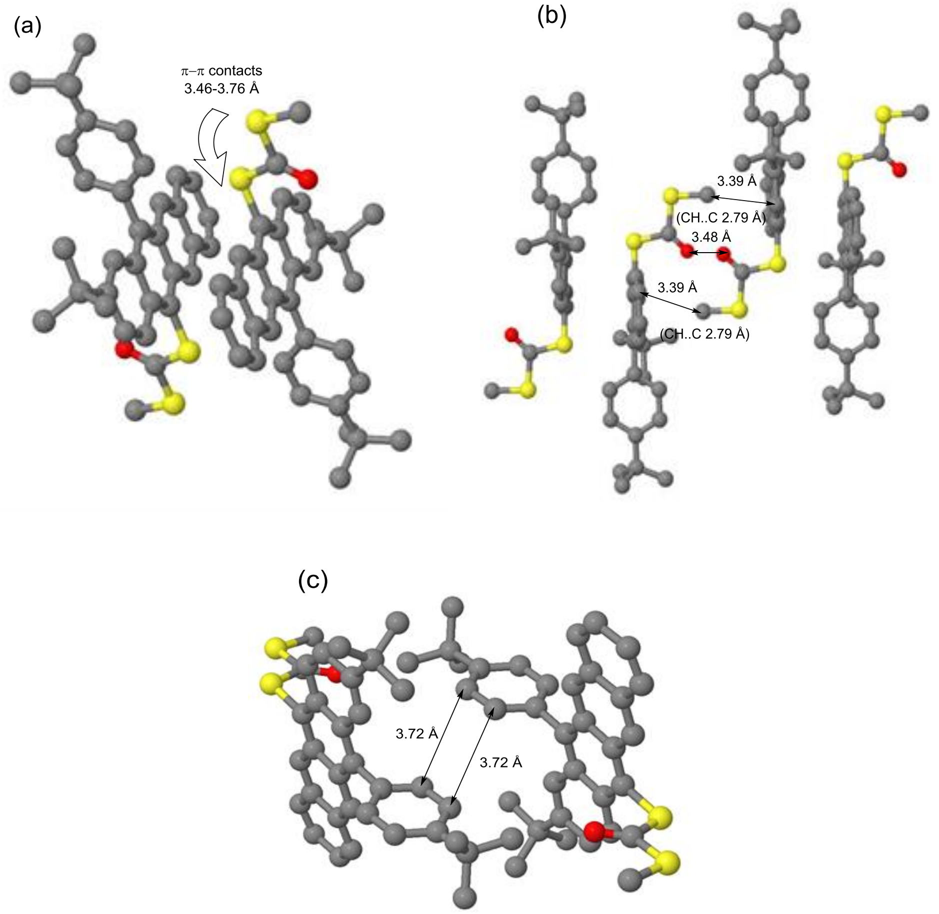

*5-(Methylthiocarbonylthio)tetracene (**7j**)*

Ribbons of **7j** are aligned in a herringbone arrangement through CH…C_π contacts (Figure S7a). Each of the ribbons is linked to the next through xanthate-to-xanthate interactions (Figure S7b).

Figure S7. (a) Edge view of herringbone packing in **7j**. (b) Inter-ribbon packing.

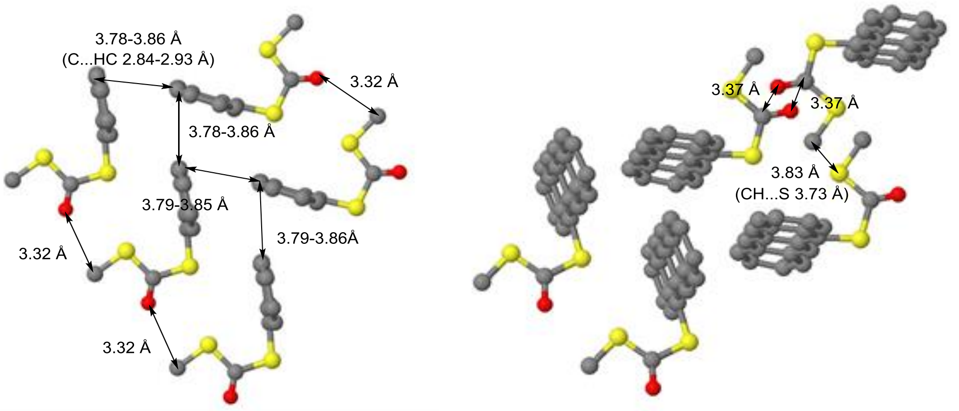

*Comparisons between the structures **7***

The structures **7** show a wide range of structural motifs that are summarised in Table S3. The greatest commonality between them is the presence of xanthate-xanthate and/or xanthate CH…C_{π} contacts which help order all the intermolecular structures. Only the least substituted tetracene **7h** shows a herringbone arrangement. This is known to be associated with lower substituted acenes (*i.e.* the van der Waals total volume of 12 hydrogen substituents in tetracene is 51.3 Å^3.[14] Aryl CH…C_{π} association is also seen in structure **7e**. Substituted **7a** and **7f** show broadly similar facially associated dimers, although the greater steric demands in **7f** produces an off-set. Both are further associated *via* xanthate contacts. Electronic biasing in tetracenes **7c-e** produces significant modification of the association modes, the methoxy substitutued **7c** and **7e** leading to partial brickwork structures, while CF_3 subsituted **7d** forms a stepped structural motif.

Table S3. Comparison of total volume of substituents (V_{subs}, Å^3) with observed intermolecular packing motifs.

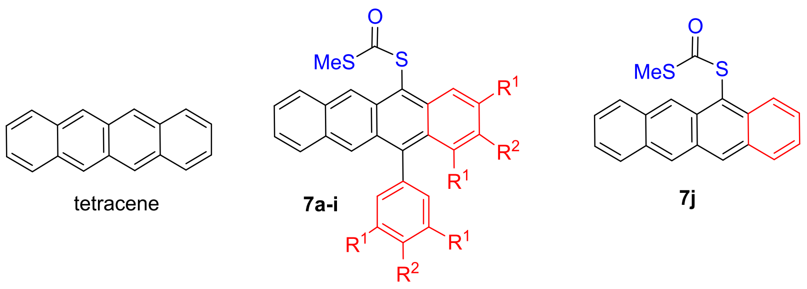

Structure	R^1	R^2	V_{subs} (Å^3)	Structural motif(s)
tetracene	-	-	51.3	herringbone
**7a**	H	H	201.7	dimer-of-dimer, infilling
**7d**	OMe	H	374.4	brickwork
**7f**	H	CF_3	272.7	perpendicular step stacks
**7g**	H	OMe	253.9	dimers, brickwork, CH..C stacking
**7i**	H	*t*-Bu	348.6	dimer-of-dimer
**7j**	H	H	129.1	herringbone ribbons

(3,4-di((Z)-benzylidene)-(1,2-(methylthiocarbonylthio)*cyclobutane-1,2-diyl)dibenzene (**15**)*

The connectivity of **15** is shown in Figure S8 together with a selected number of intramolecular distances. Only a very small amount of this material was attained which was unstable in solution

Figure S8. Molecular structure of cyclobutane **15**.

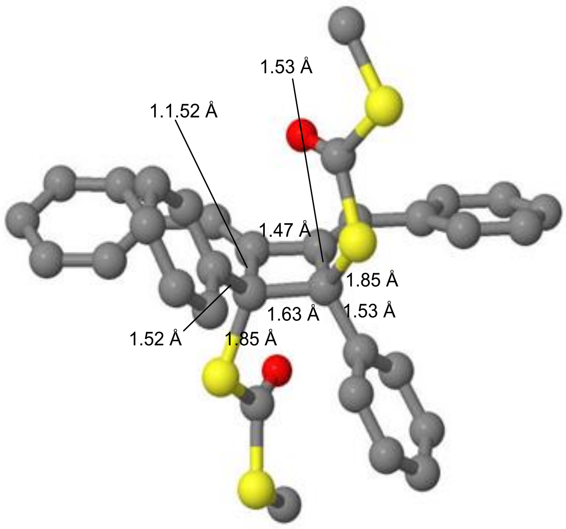

6. Spectroscopic data (all compounds)

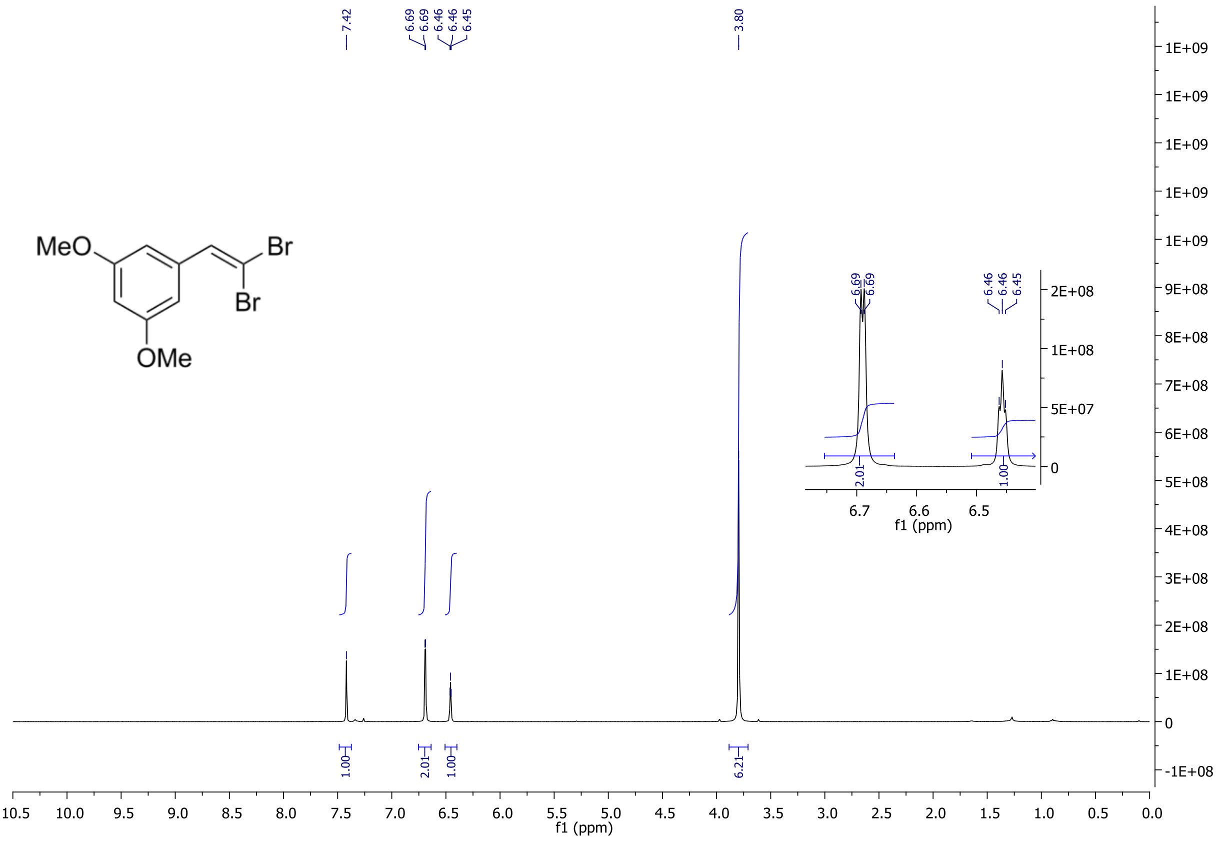
MeO
Br
Br
OMe
7.42
6.69
6.69
6.46
6.46
6.45
3.80
1.00
2.01
1.00
6.21
f1 (ppm)

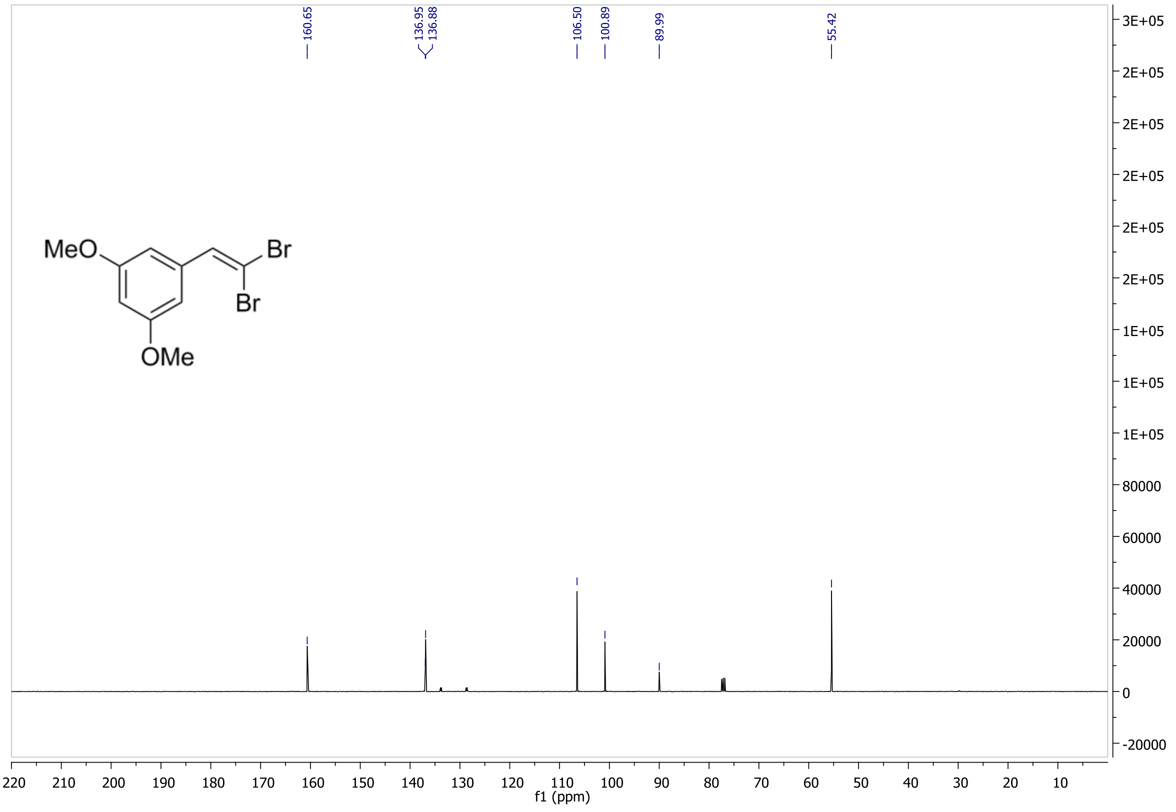
MeO
Br
Br
OMe
160.65
136.95
136.88
106.50
100.89
89.99
55.42
f1 (ppm)

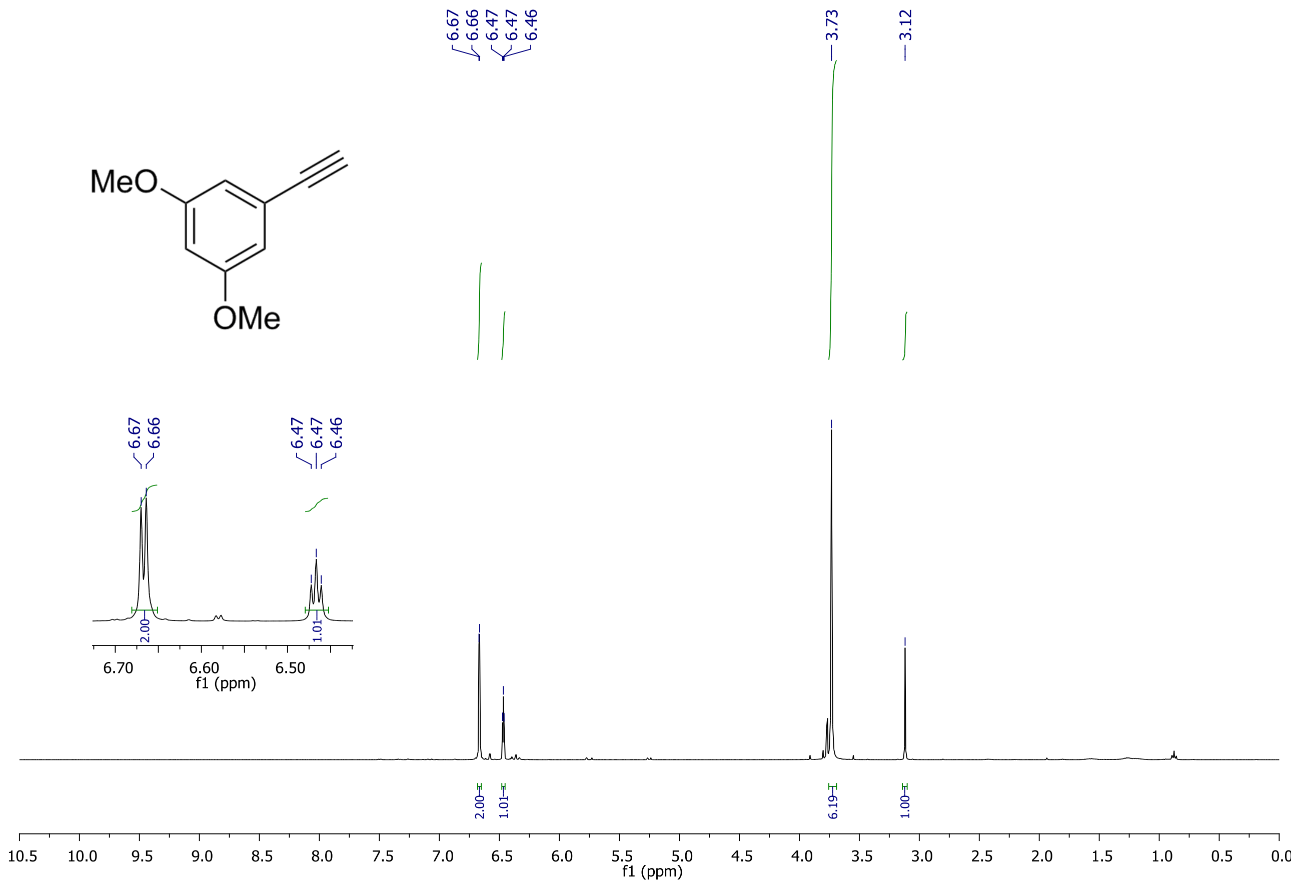

MeO
OMe
6.67
6.66
6.47
6.47
6.46
3.73
3.12
2.00
1.01
6.70
6.60
6.50
f1 (ppm)
6.19
1.00
10.5
10.0
9.5
9.0
8.5
8.0
7.5
7.0
6.5
6.0
5.5
5.0
4.5
4.0
3.5
3.0
2.5
2.0
1.5
1.0
0.5
f1 (ppm)

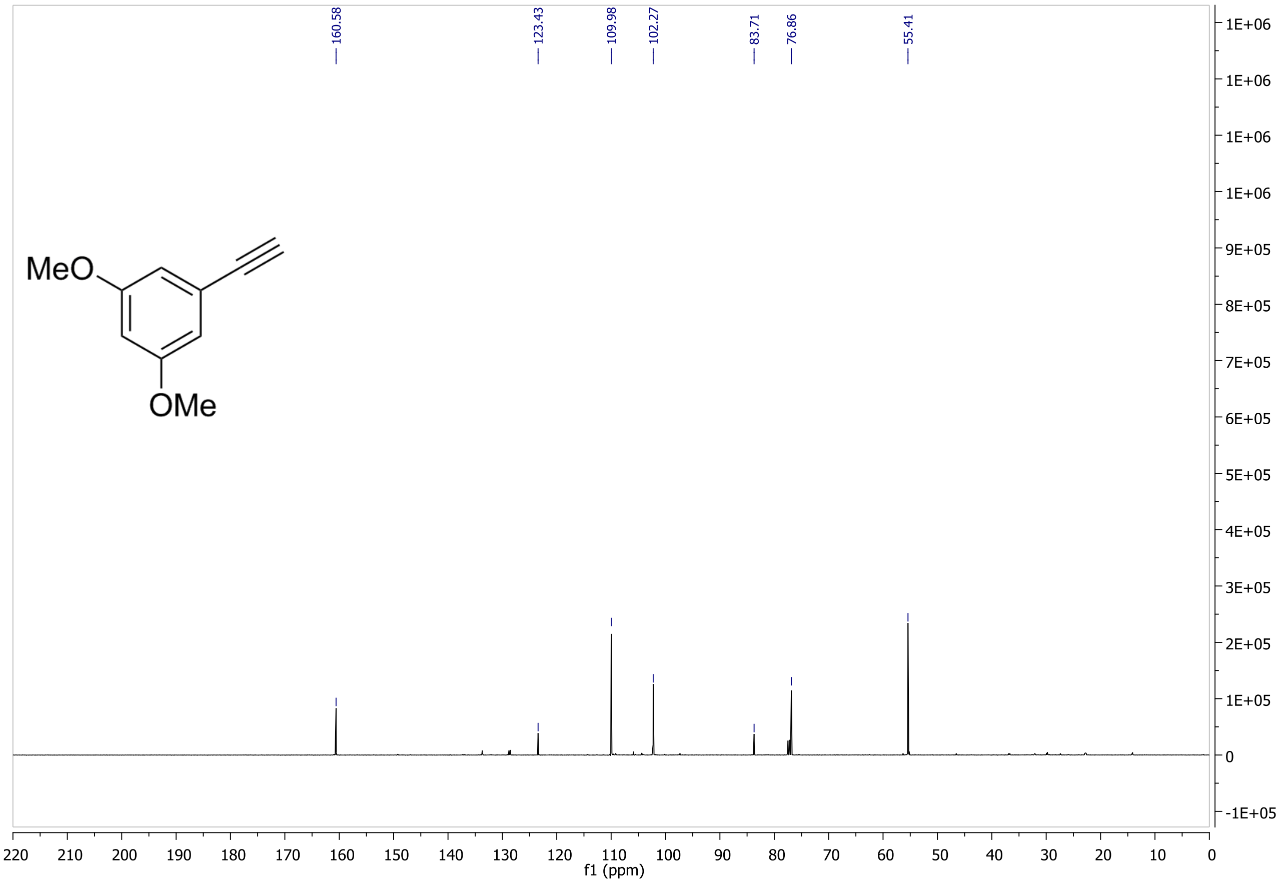

160.58
123.43
109.98
102.27
83.71
76.86
55.41
MeO
OMe
1E+06
9E+05
8E+05
7E+05
6E+05
5E+05
4E+05
3E+05
2E+05
1E+05
0
-1E+05
220
210
200
190
180
170
160
150
140
130
120
110
100
90
80
70
60
50
40
30
20
10
0
f1 (ppm)

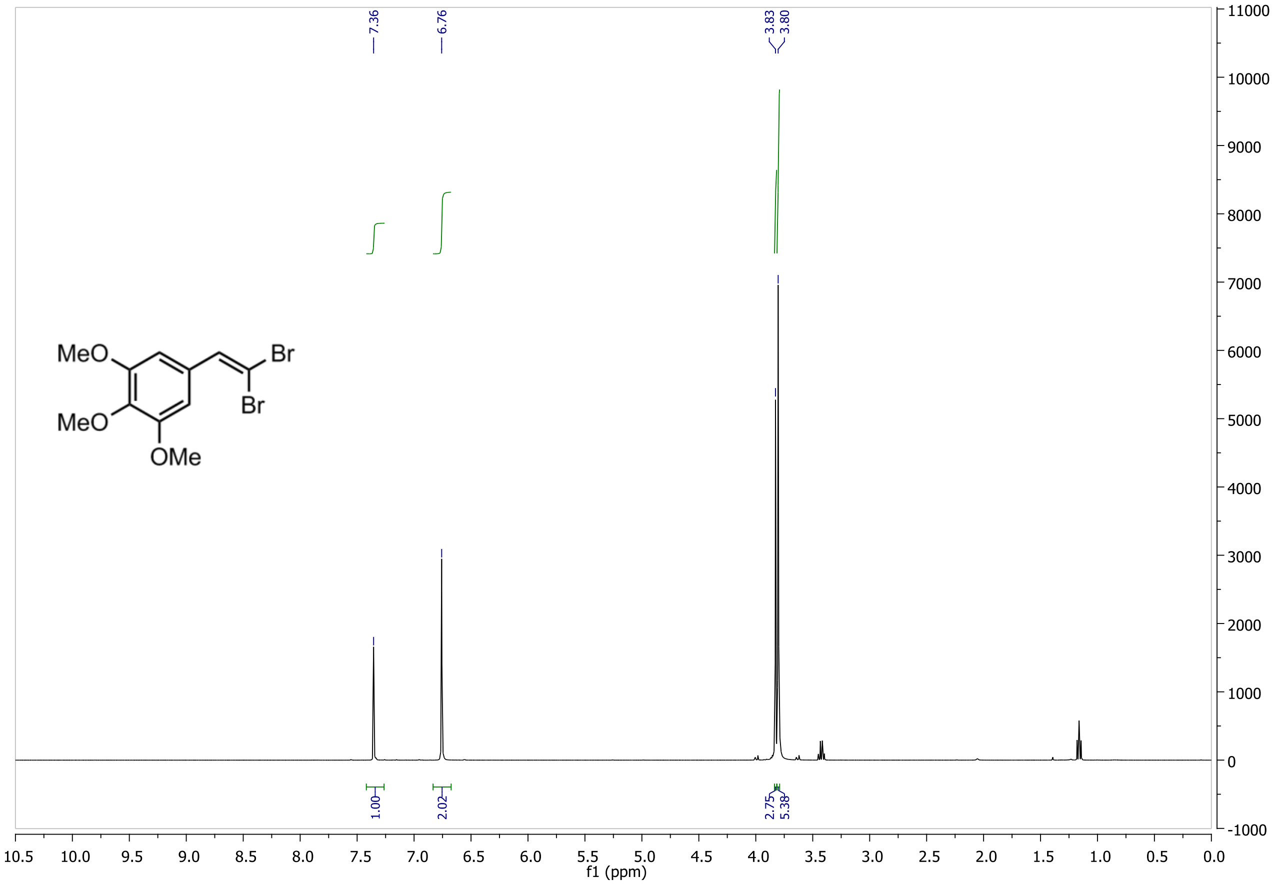
7.36
6.76
3.83
3.80
1.00
2.02
2.75
5.38
MeO
MeO
OMe
Br
Br
f1 (ppm)

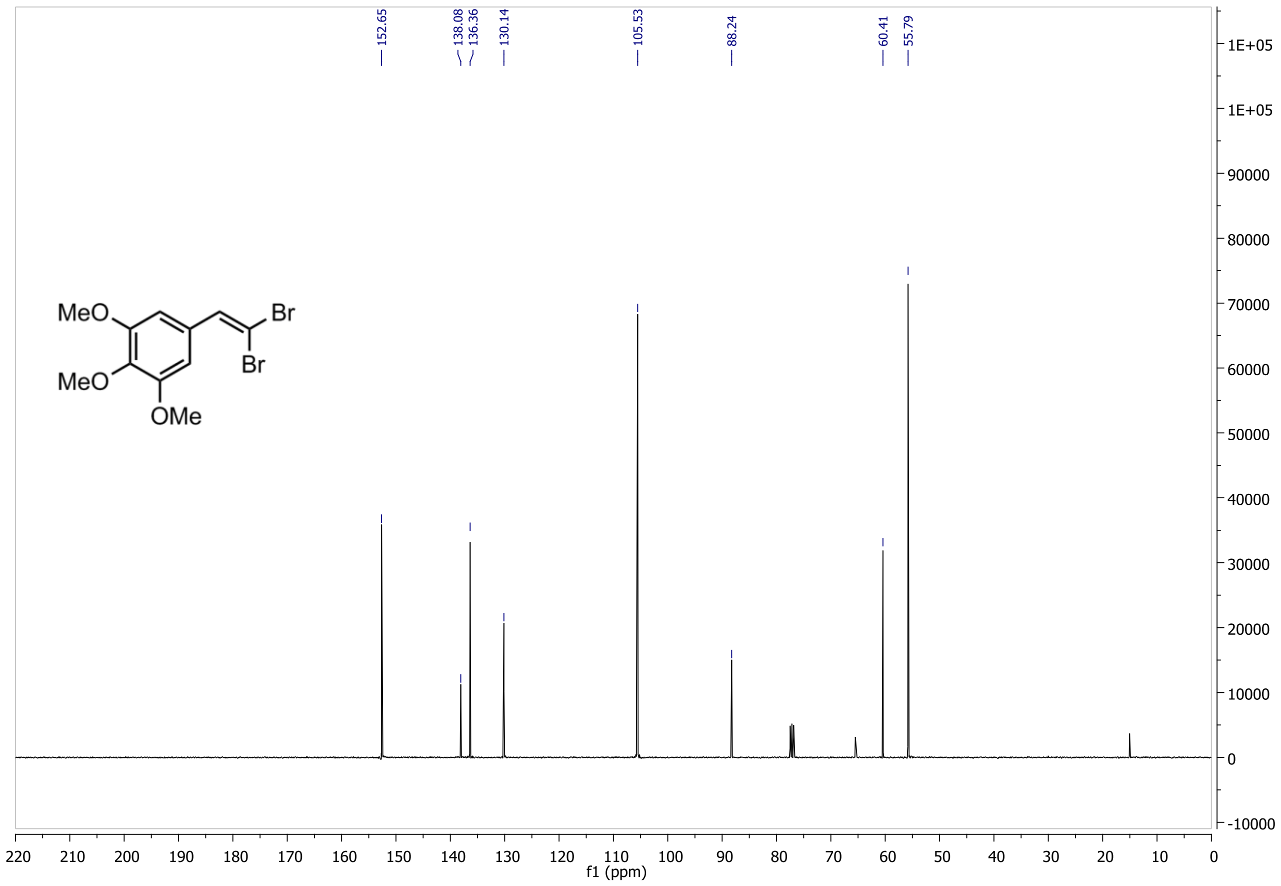
152.65
138.08
136.36
130.14
105.53
88.24
60.41
55.79
MeO
MeO
OMe
Br
Br
f1 (ppm)

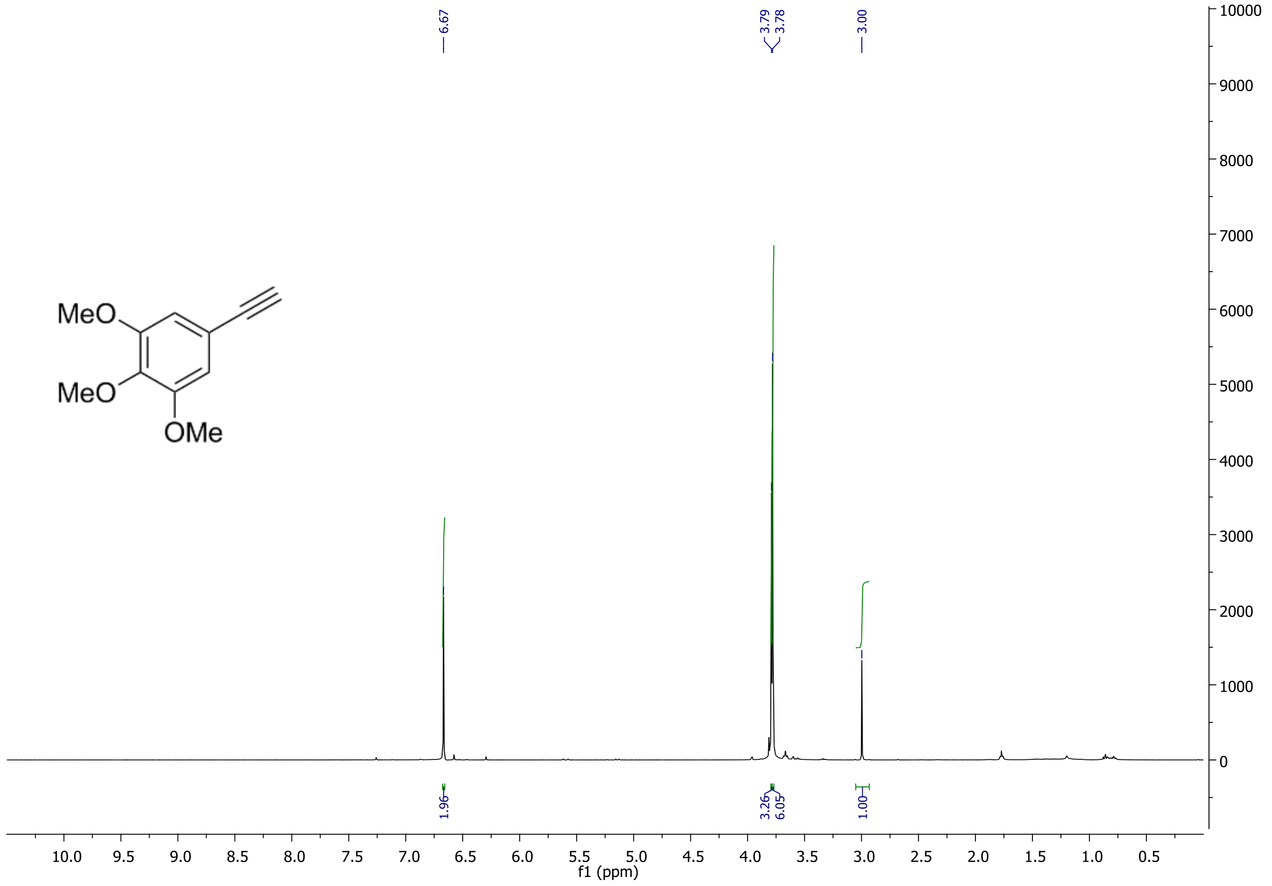
MeO
MeO
OMe
6.67
3.79
3.78
3.00
1.96
3.26
6.05
1.00
f1 (ppm)

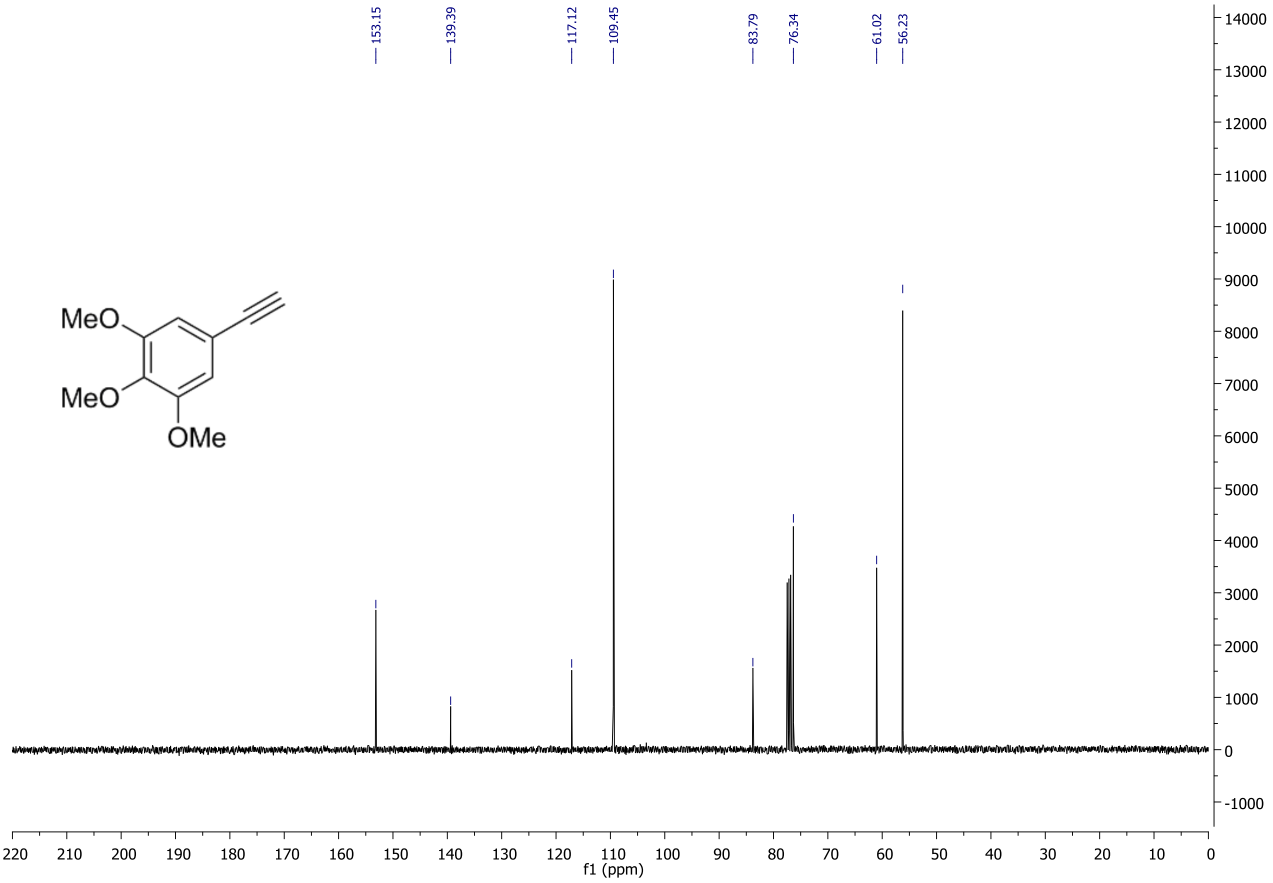
MeO
MeO
OMe
153.15
139.39
117.12
109.45
83.79
76.34
61.02
56.23
f1 (ppm)

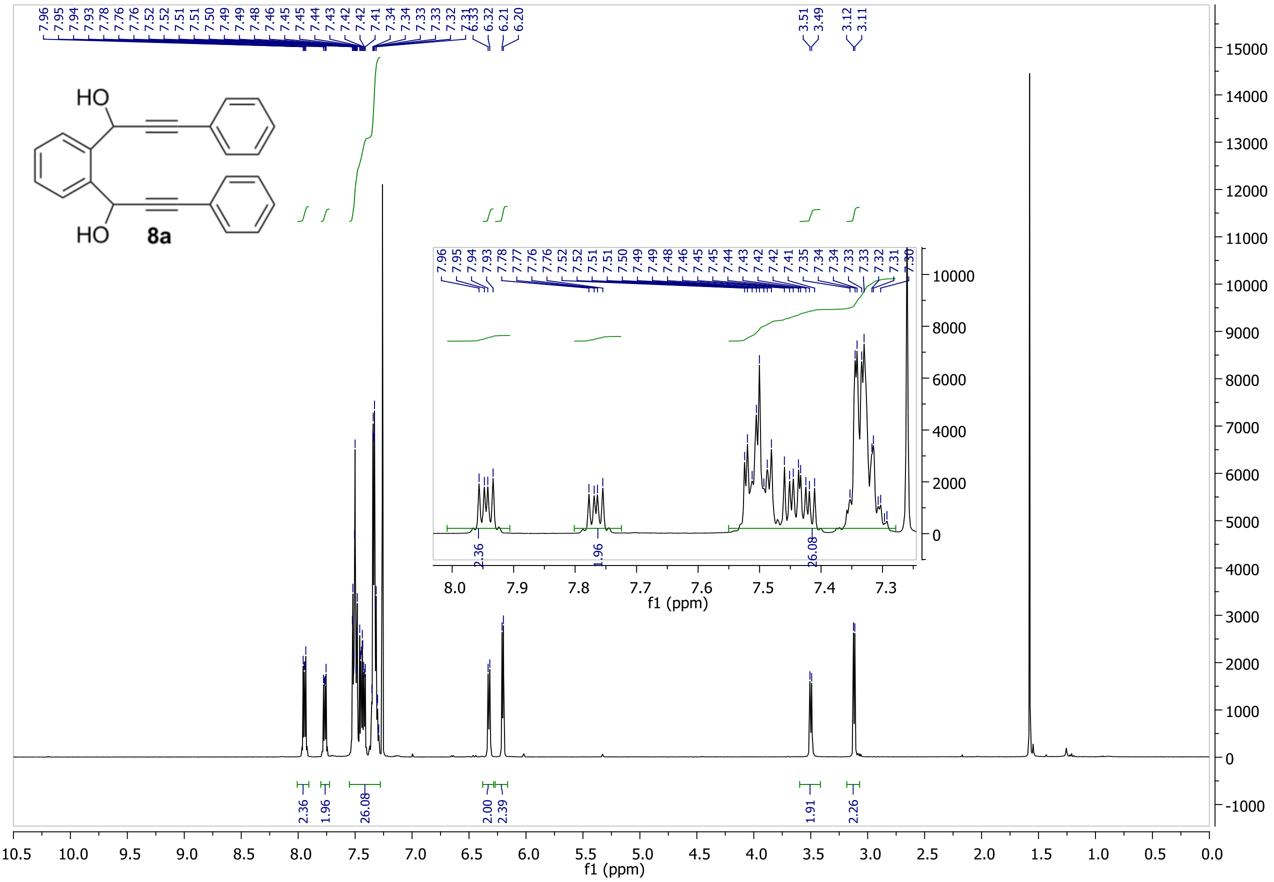
7.96 7.95 7.94 7.93 7.78 7.76 7.76 7.52 7.52 7.51 7.51 7.50 7.49 7.49 7.48 7.46 7.45 7.45 7.44 7.43 7.42 7.42 7.41 7.34 7.34 7.33 7.33 7.32 7.31 6.33 6.32 6.21 6.20
3.51 3.49 3.12 3.11
HO
HO
8a
2.36 1.96 26.08 2.00 2.39 1.91 2.26
f1 (ppm)

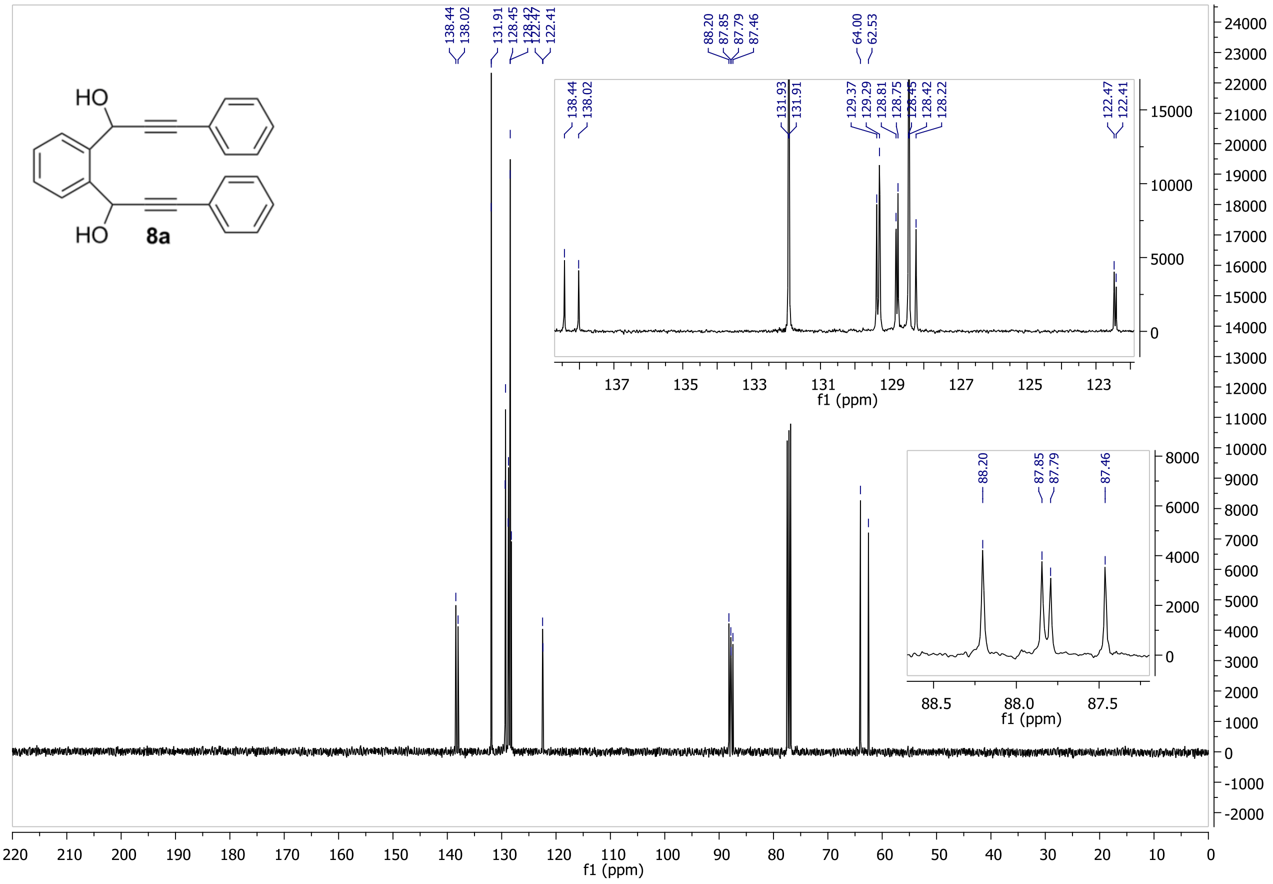
138.44 138.02 131.91 128.45 128.42 122.47 122.41
88.20 87.85 87.79 87.46
64.00 62.53
HO
HO
8a
138.44 138.02 131.93 131.91 129.37 129.29 128.81 128.75 128.45 128.42 128.22 122.47 122.41
88.20 87.85 87.79 87.46
f1 (ppm)

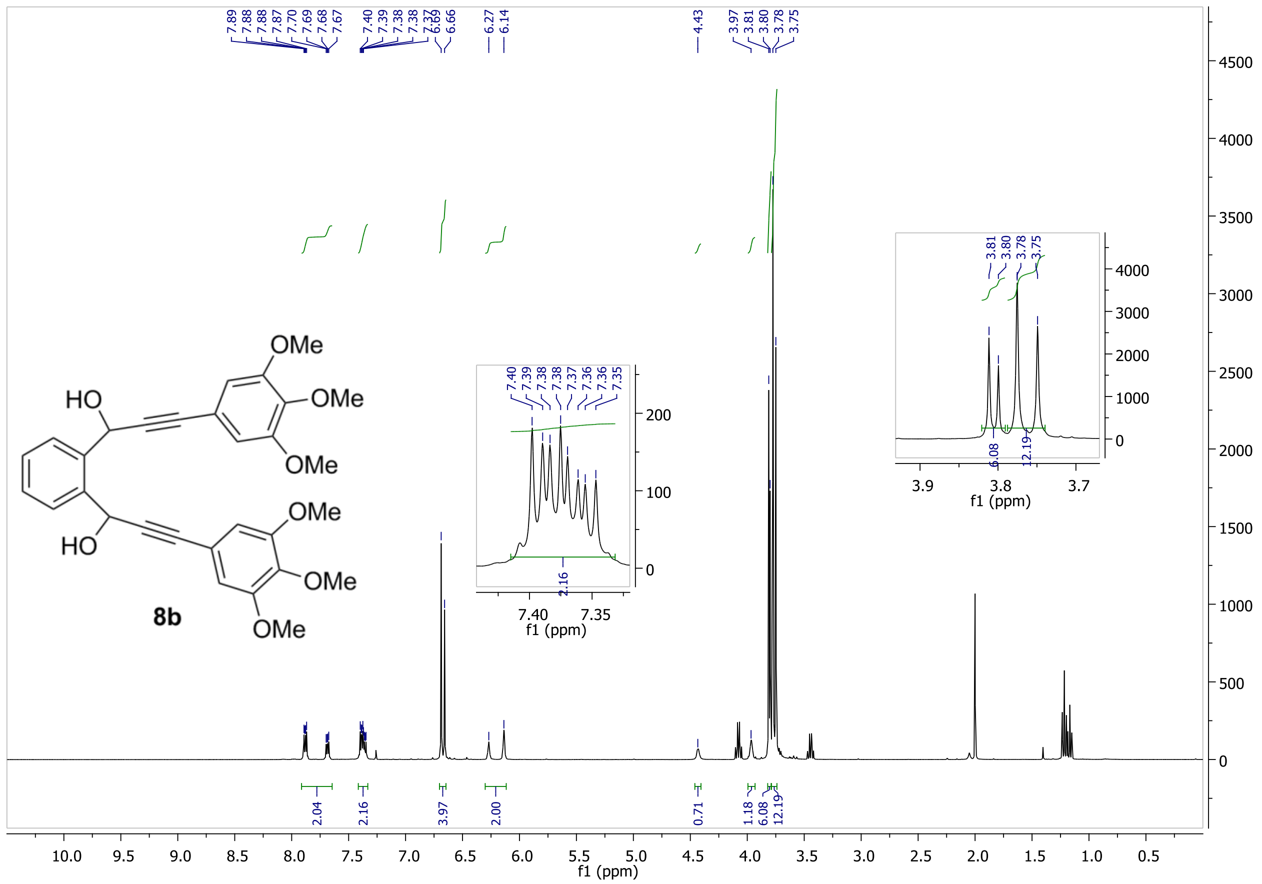
7.89 7.88 7.88 7.87 7.70 7.69 7.68 7.67 7.40 7.39 7.38 7.38 7.37 6.69 6.66 6.27 6.14 4.43 3.97 3.81 3.80 3.78 3.75
HO
OMe
OMe
OMe
HO
OMe
OMe
OMe
8b
2.04 2.16 3.97 2.00 0.71 1.18 6.08 12.19
f1 (ppm)

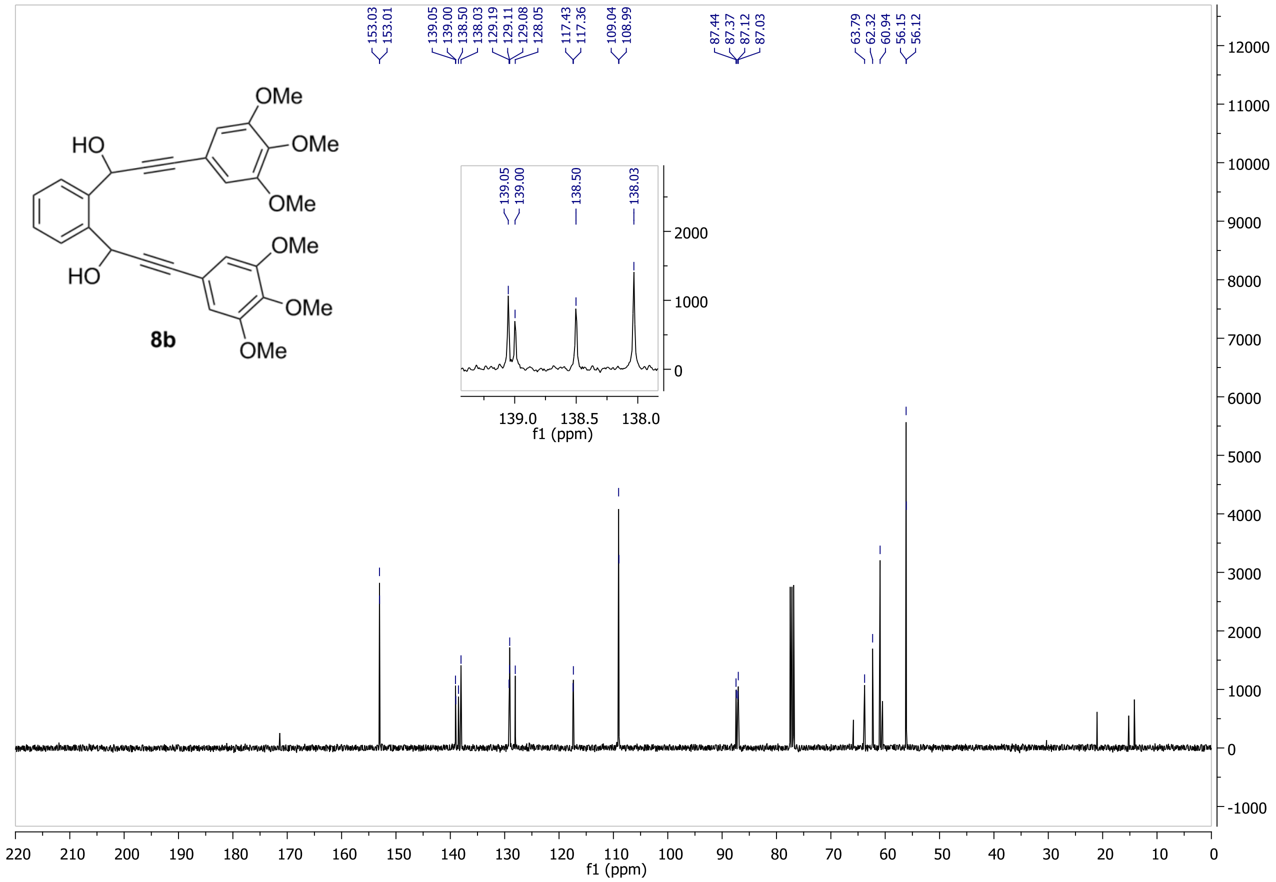
153.03 153.01 139.05 139.00 138.50 138.03 129.19 129.11 129.08 128.05 117.43 117.36 109.04 108.99 87.44 87.37 87.12 87.03 63.79 62.32 60.94 56.15 56.12
HO
OMe
OMe
OMe
HO
OMe
OMe
OMe
8b
f1 (ppm)

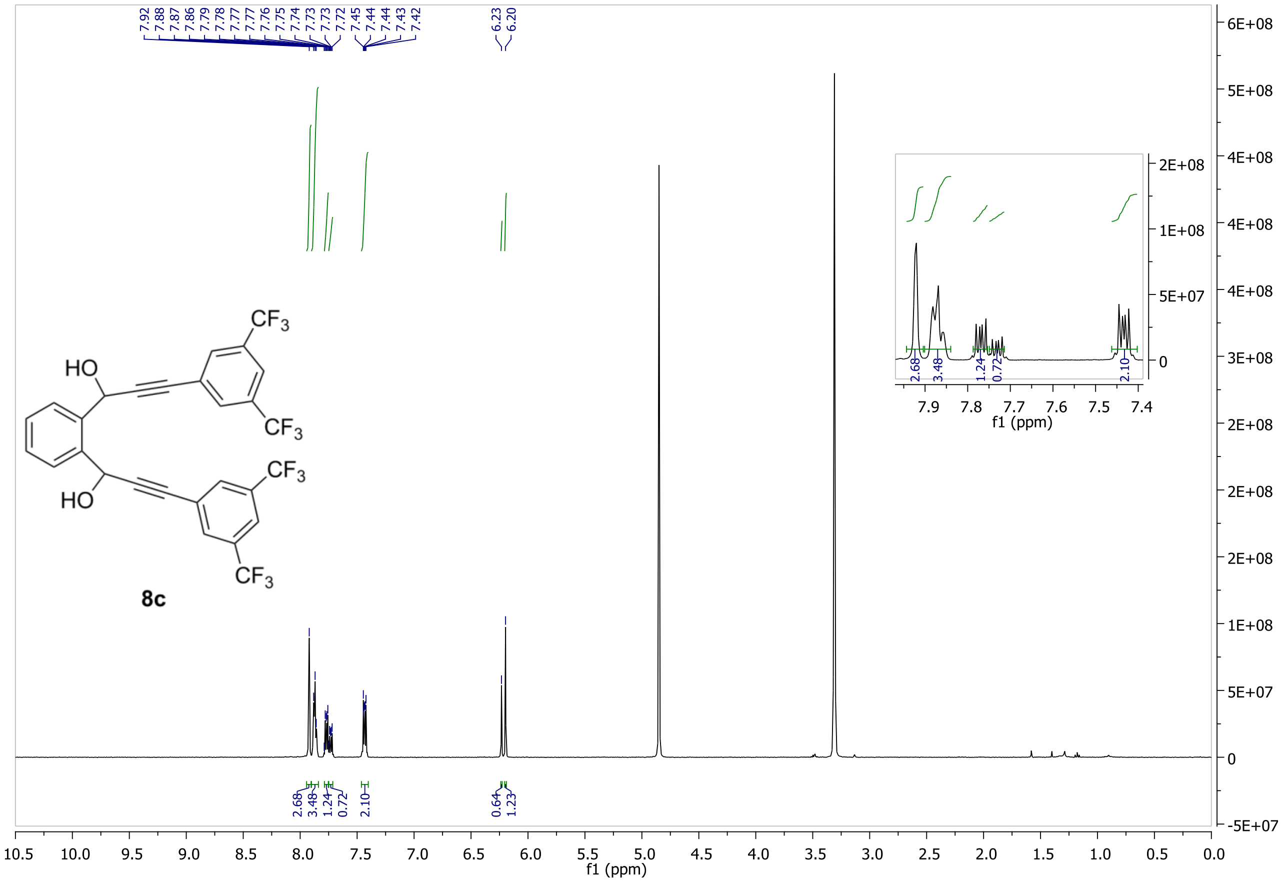

7.92
7.88
7.87
7.86
7.79
7.78
7.77
7.77
7.76
7.75
7.74
7.73
7.73
7.72
7.45
7.44
7.44
7.43
7.42
6.23
6.20
HO
HO
CF_3
CF_3
CF_3
CF_3
8c
2.68
3.48
1.24
0.72
2.10
0.64
1.23
f1 (ppm)

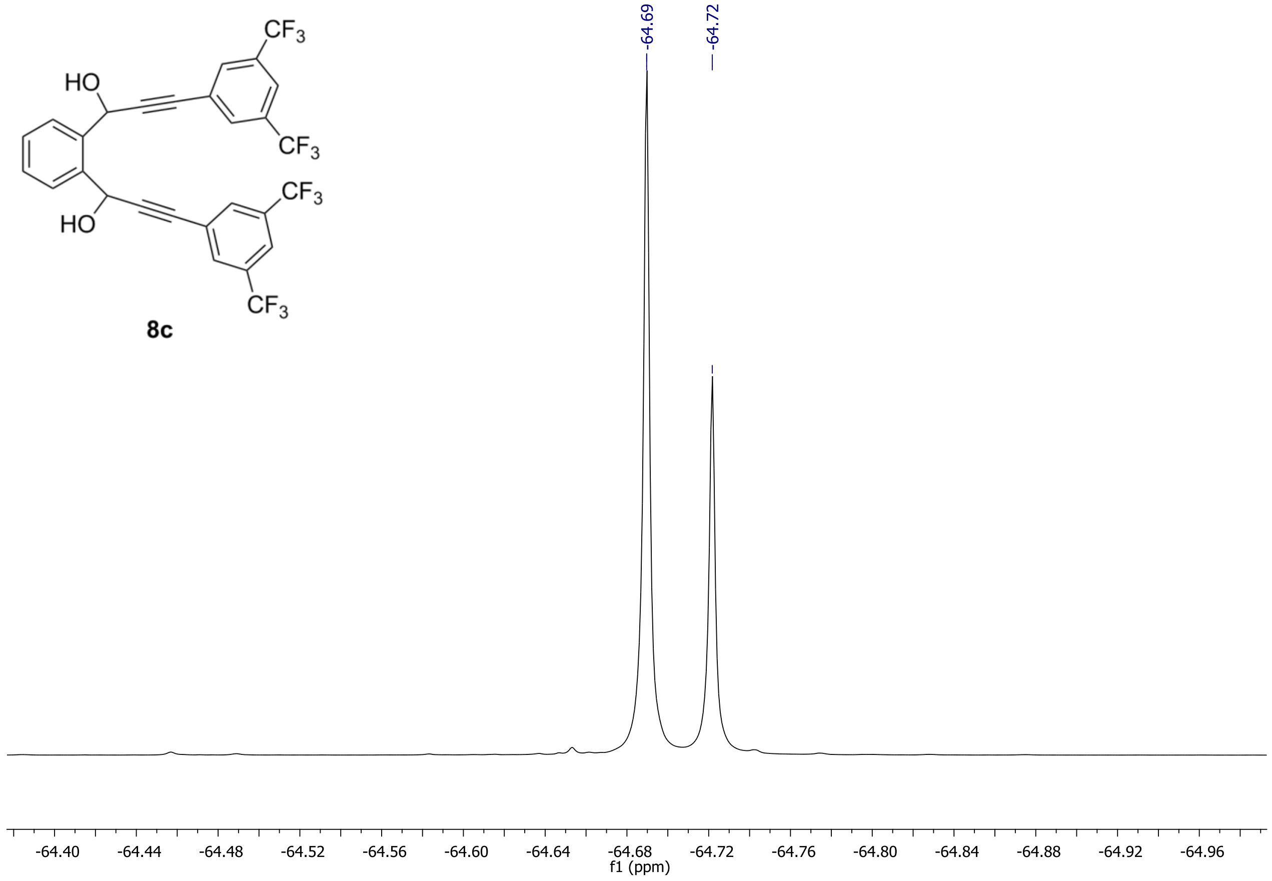

-64.69
-64.72
HO
HO
CF_3
CF_3
CF_3
CF_3
8c
f1 (ppm)

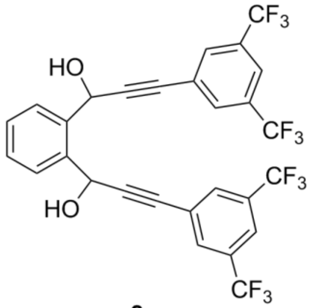

HO
CF3
CF3
CF3
HO
CF3

8c

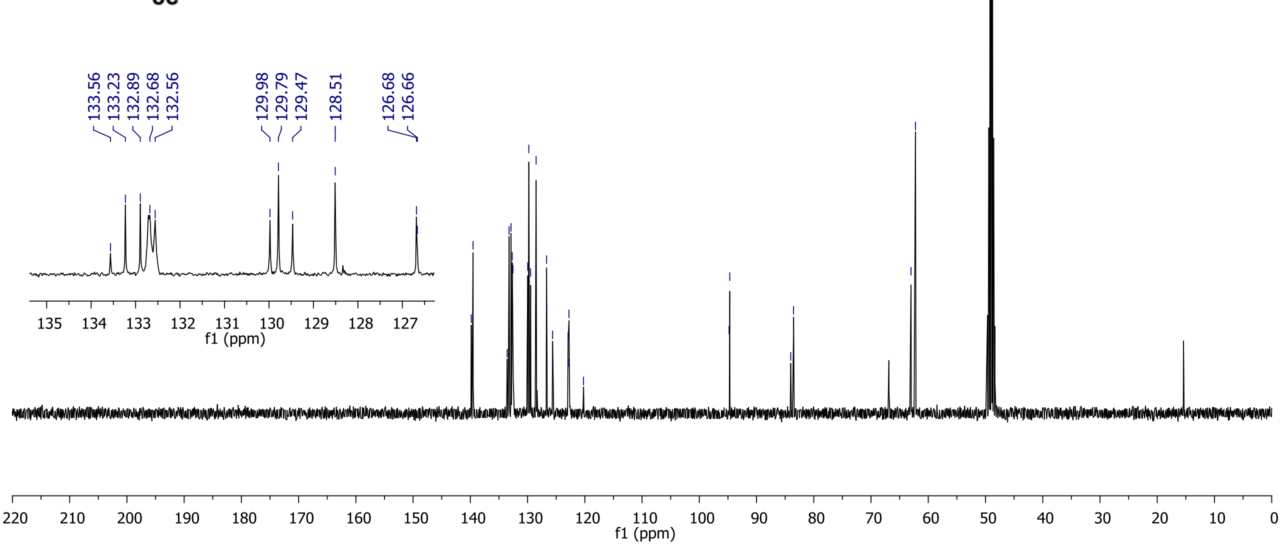

139.84
139.52
133.56
133.23
132.89
132.68
132.56
129.98
129.79
129.47
128.51
126.68
126.66
125.63
125.59
122.93
122.88
122.77
120.22
94.80
94.68
84.03
83.53
63.02
62.23
133.56
133.23
132.89
132.68
132.56
129.98
129.79
129.47
128.51
126.68
126.66
135 134 133 132 131 130 129 128 127
f1 (ppm)
220 210 200 190 180 170 160 150 140 130 120 110 100 90 80 70 60 50 40 30 20 10 0
f1 (ppm)

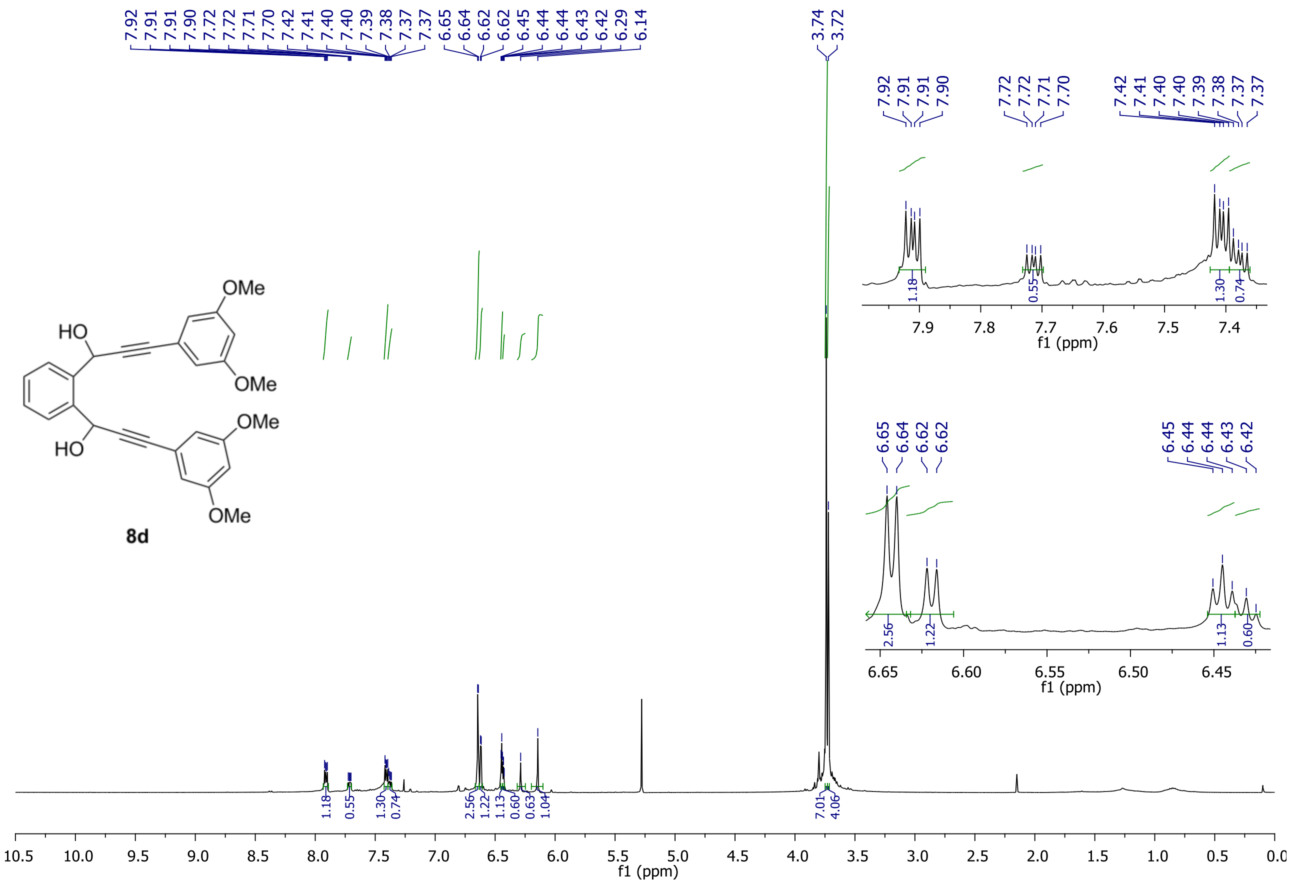
OMe
HO
OMe
OMe
HO
OMe
8d
f1 (ppm)

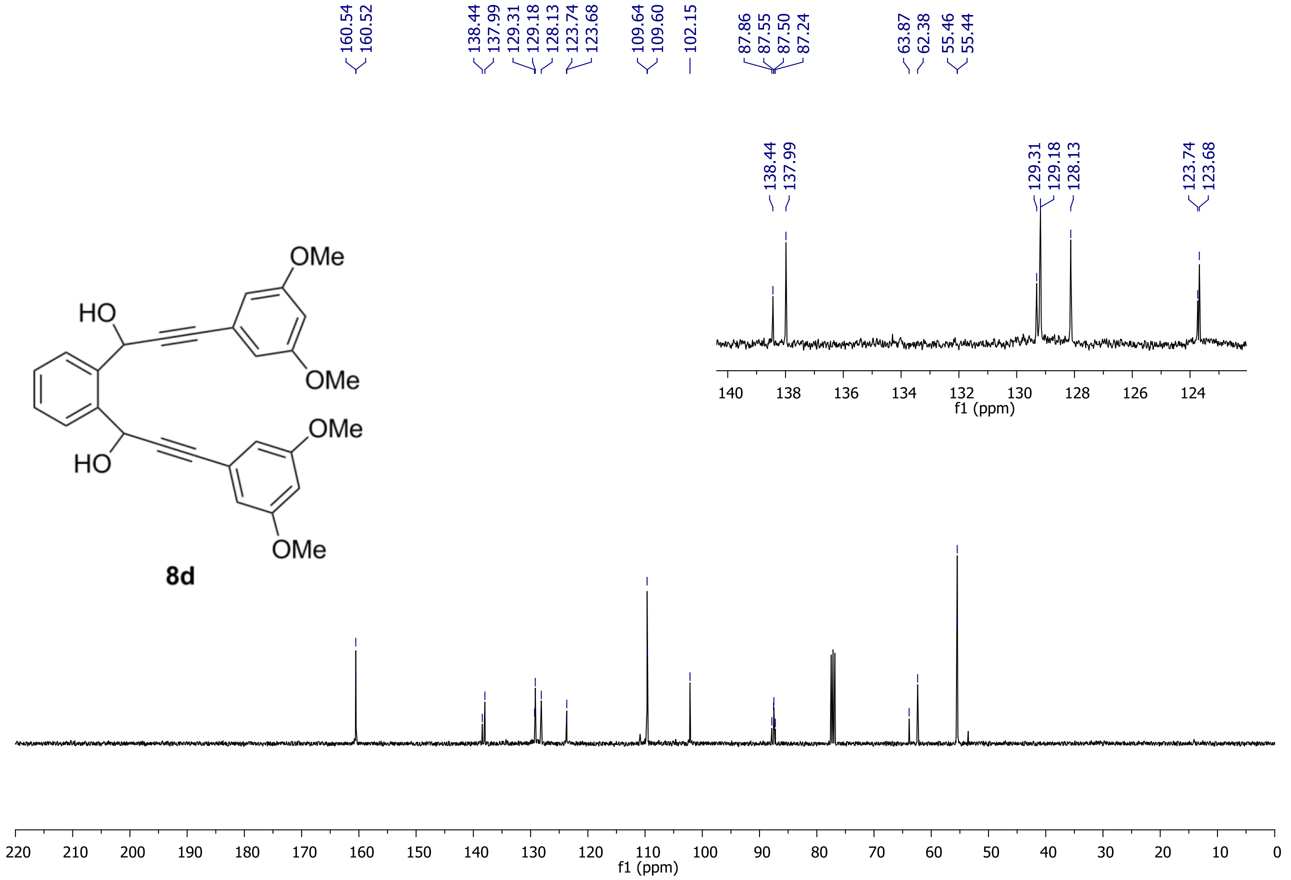
OMe
HO
OMe
OMe
HO
OMe
8d
f1 (ppm)

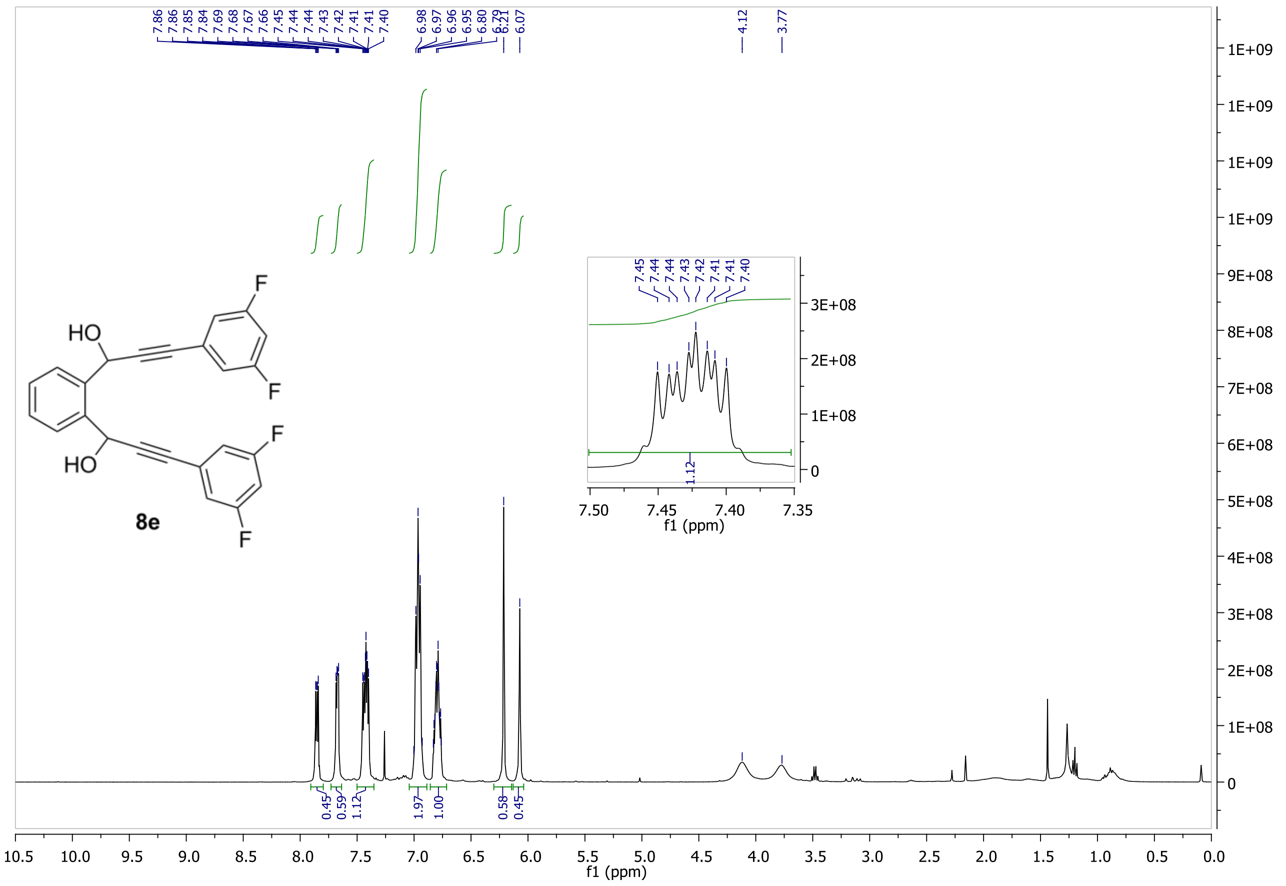
7.86
7.86
7.85
7.84
7.69
7.68
7.67
7.66
7.45
7.44
7.44
7.43
7.42
7.41
7.41
7.40
6.98
6.97
6.96
6.95
6.80
6.79
6.21
6.07
4.12
3.77
HO
HO
F
F
F
F
8e
1.12
7.50
7.45
7.40
7.35
f1 (ppm)
3E+08
2E+08
1E+08
0
0.45
0.59
1.12
1.97
1.00
0.58
0.45
1E+09
9E+08
8E+08
7E+08
6E+08
5E+08
4E+08
3E+08
2E+08
1E+08
10.5
10.0
9.5
9.0
8.5
8.0
7.5
7.0
6.5
6.0
5.5
5.0
4.5
4.0
3.5
3.0
2.5
2.0
1.5
1.0
0.5
0.0
f1 (ppm)

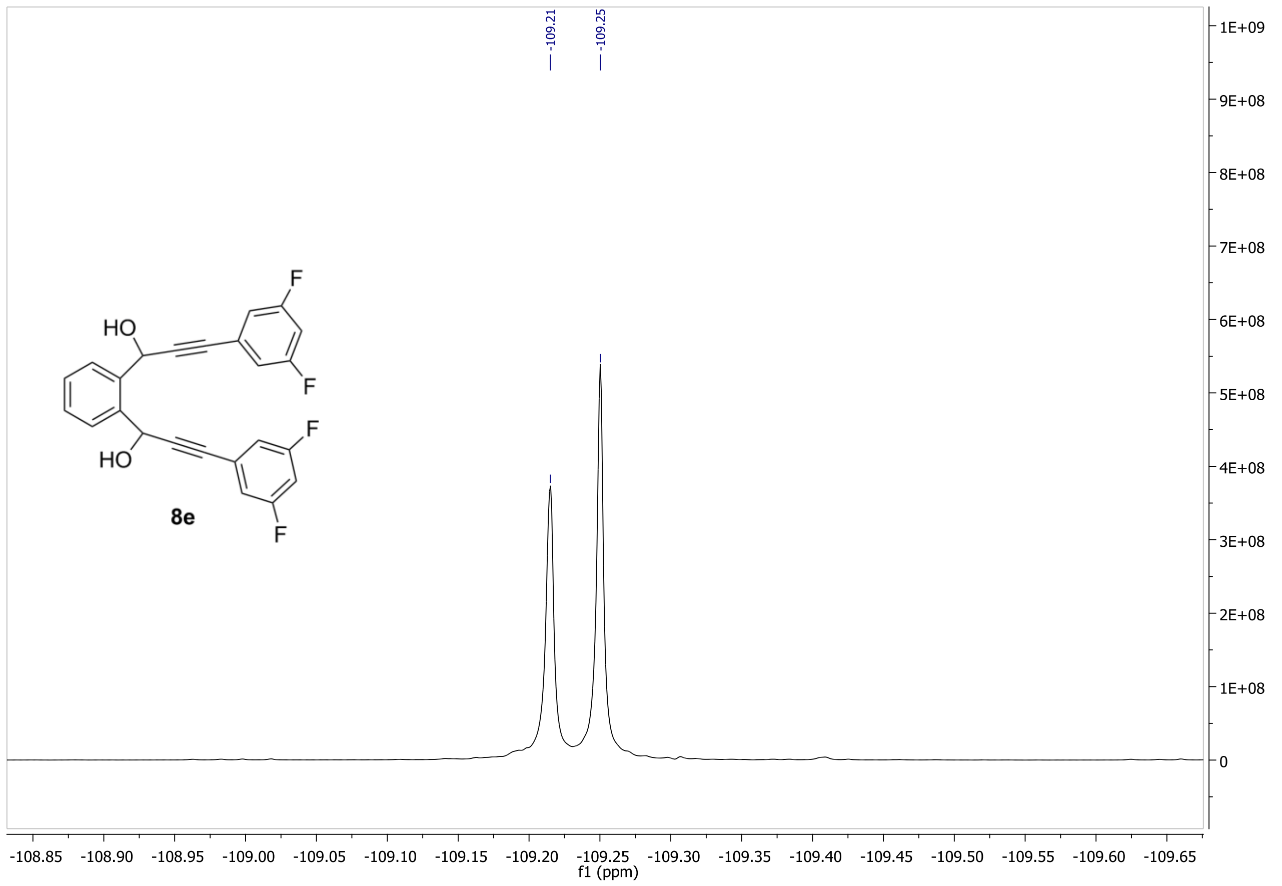
-109.21
-109.25
HO
HO
F
F
F
F
8e
1E+09
9E+08
8E+08
7E+08
6E+08
5E+08
4E+08
3E+08
2E+08
1E+08
0
-108.85
-108.90
-108.95
-109.00
-109.05
-109.10
-109.15
-109.20
-109.25
-109.30
-109.35
-109.40
-109.45
-109.50
-109.55
-109.60
-109.65
f1 (ppm)

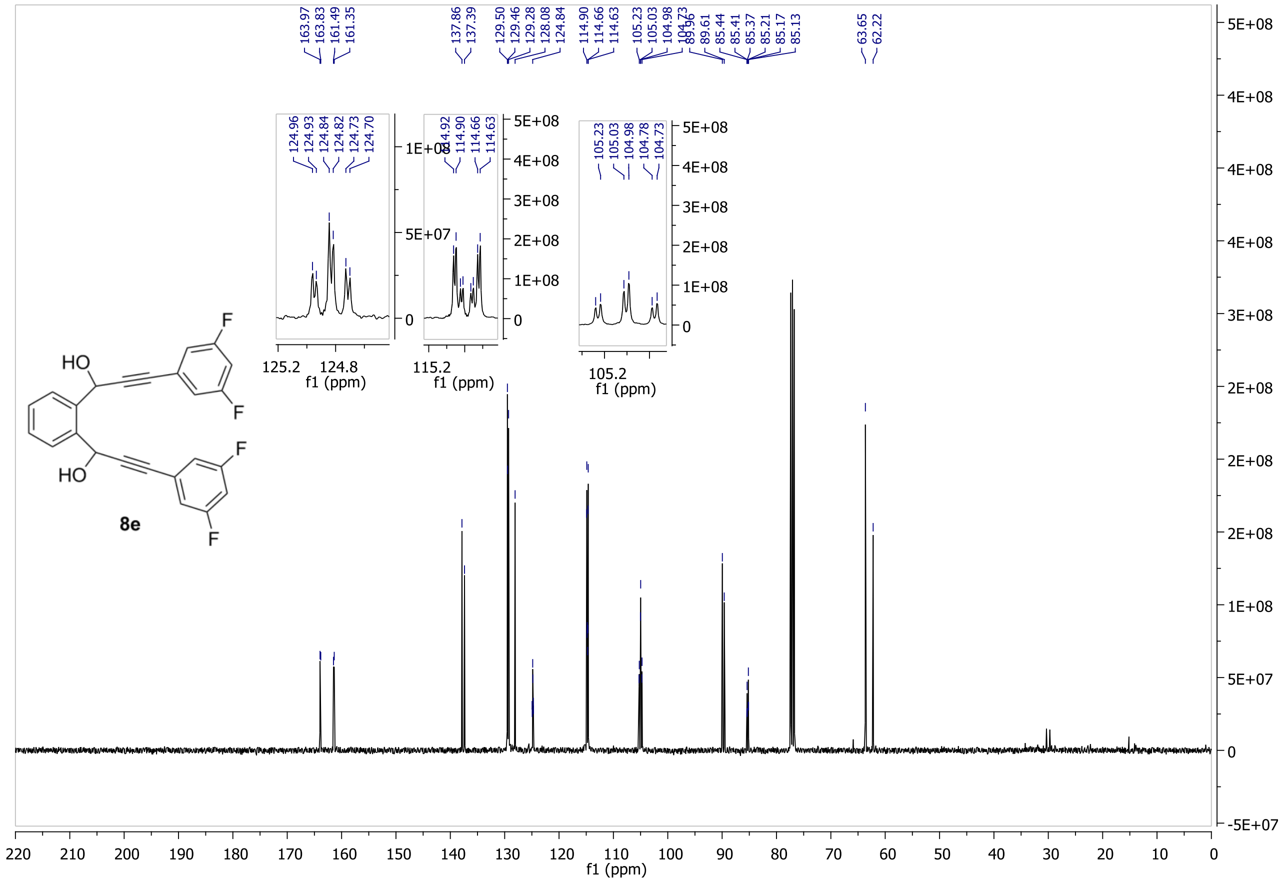
8e
f1 (ppm)

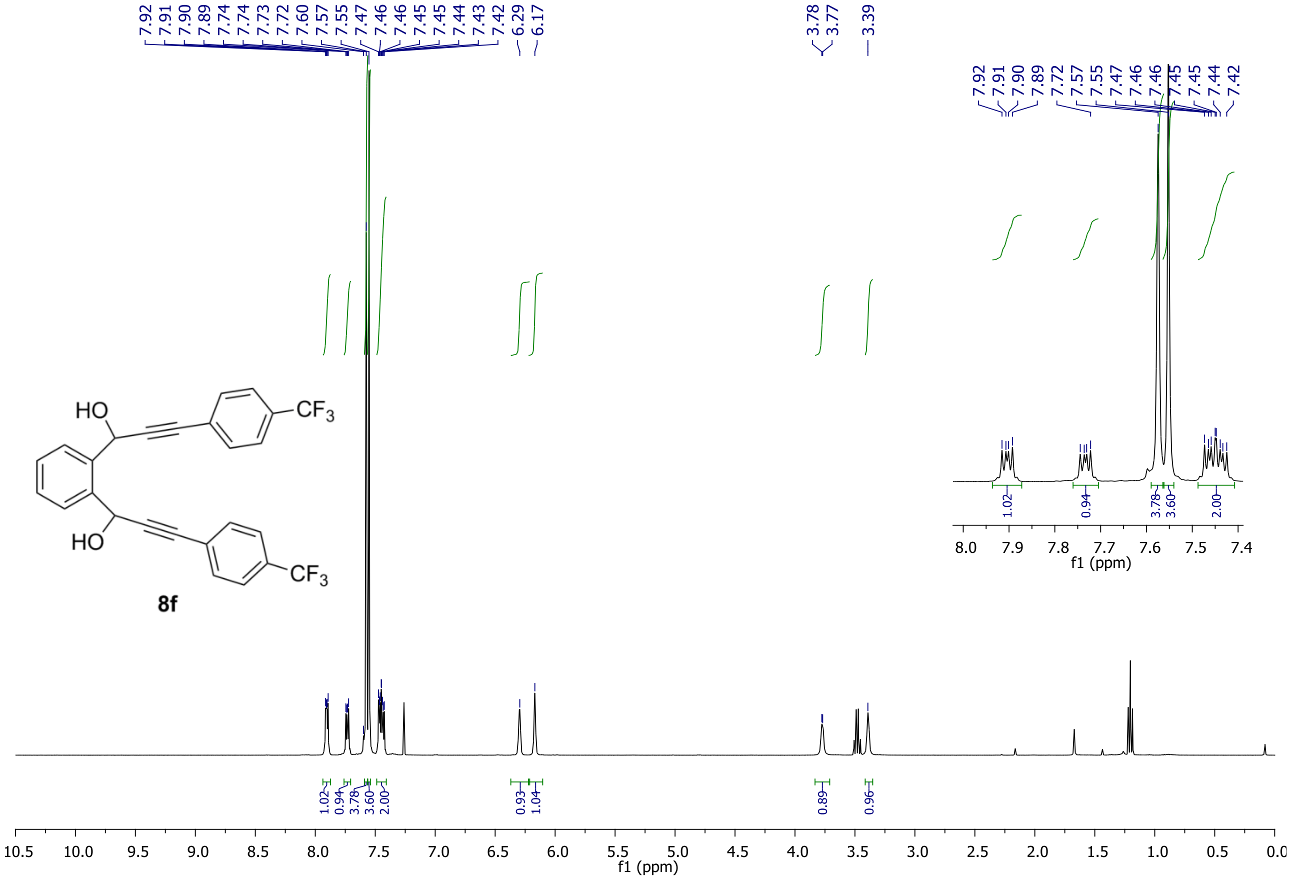
8f
f1 (ppm)

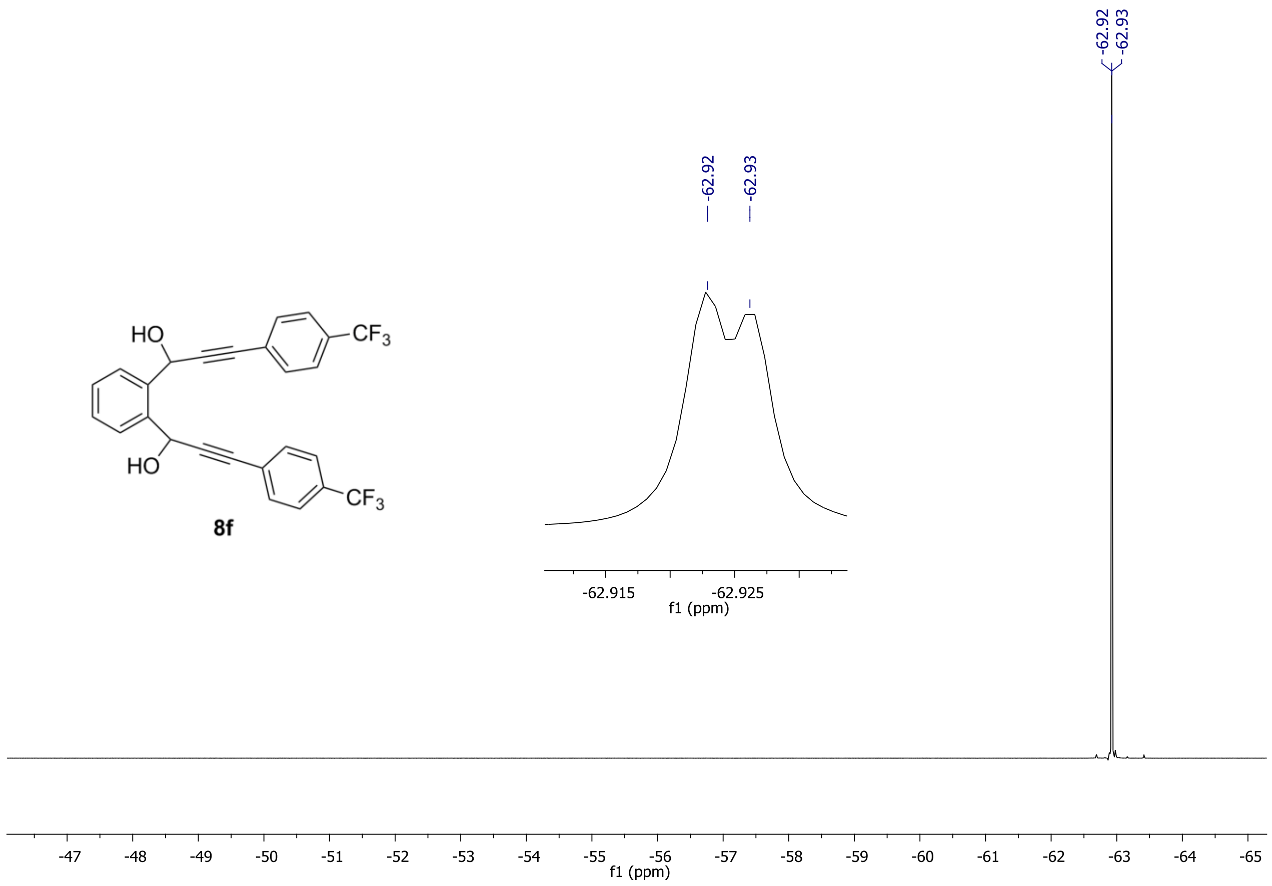
HO
CF3
HO
CF3
8f
-62.92
-62.93
-62.915
-62.925
f1 (ppm)
f1 (ppm)

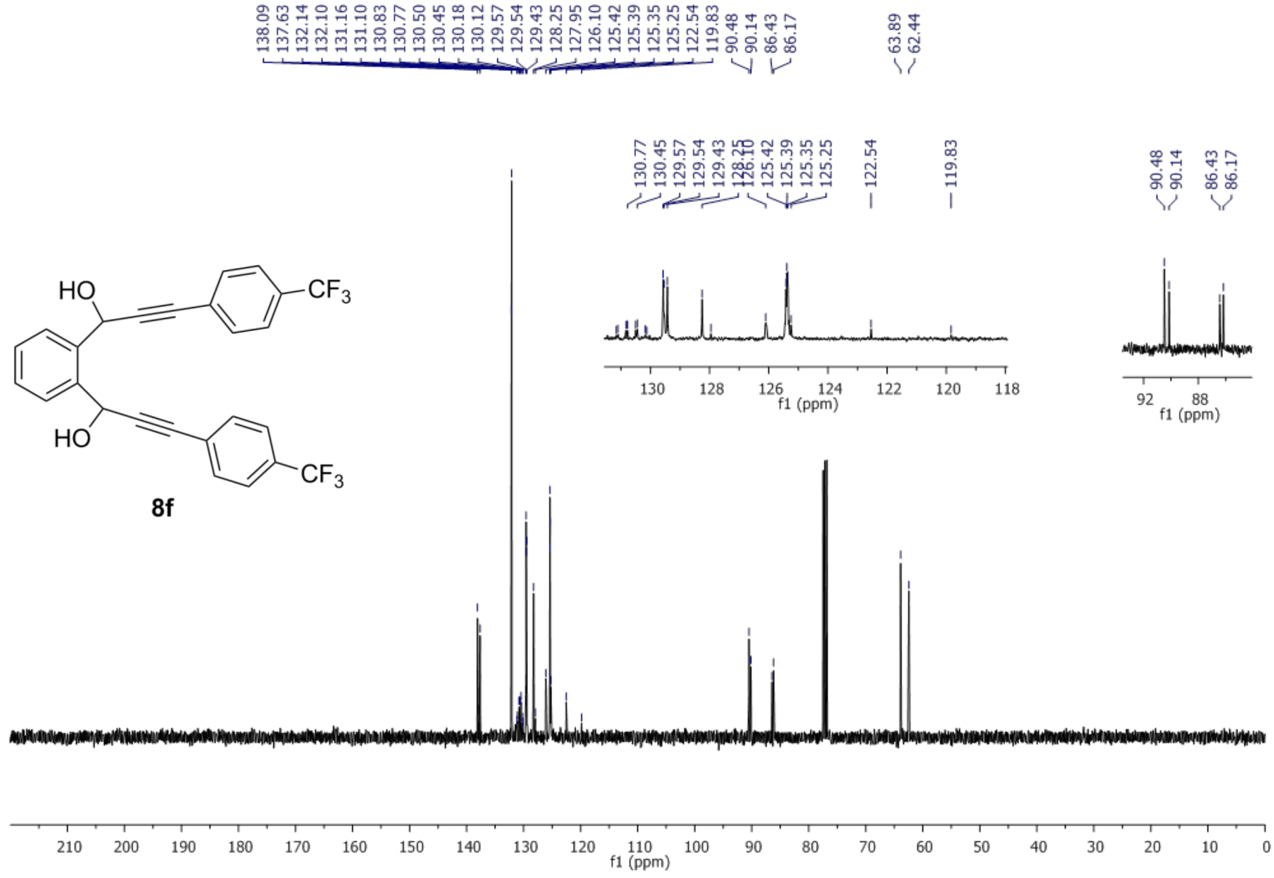
138.09
137.63
132.14
132.10
131.16
131.10
130.83
130.77
130.50
130.45
130.18
130.12
129.57
129.54
129.43
128.25
127.95
126.10
125.42
125.39
125.35
125.25
122.54
119.83
90.48
90.14
86.43
86.17
63.89
62.44
HO
CF3
HO
CF3
8f
f1 (ppm)
f1 (ppm)
f1 (ppm)

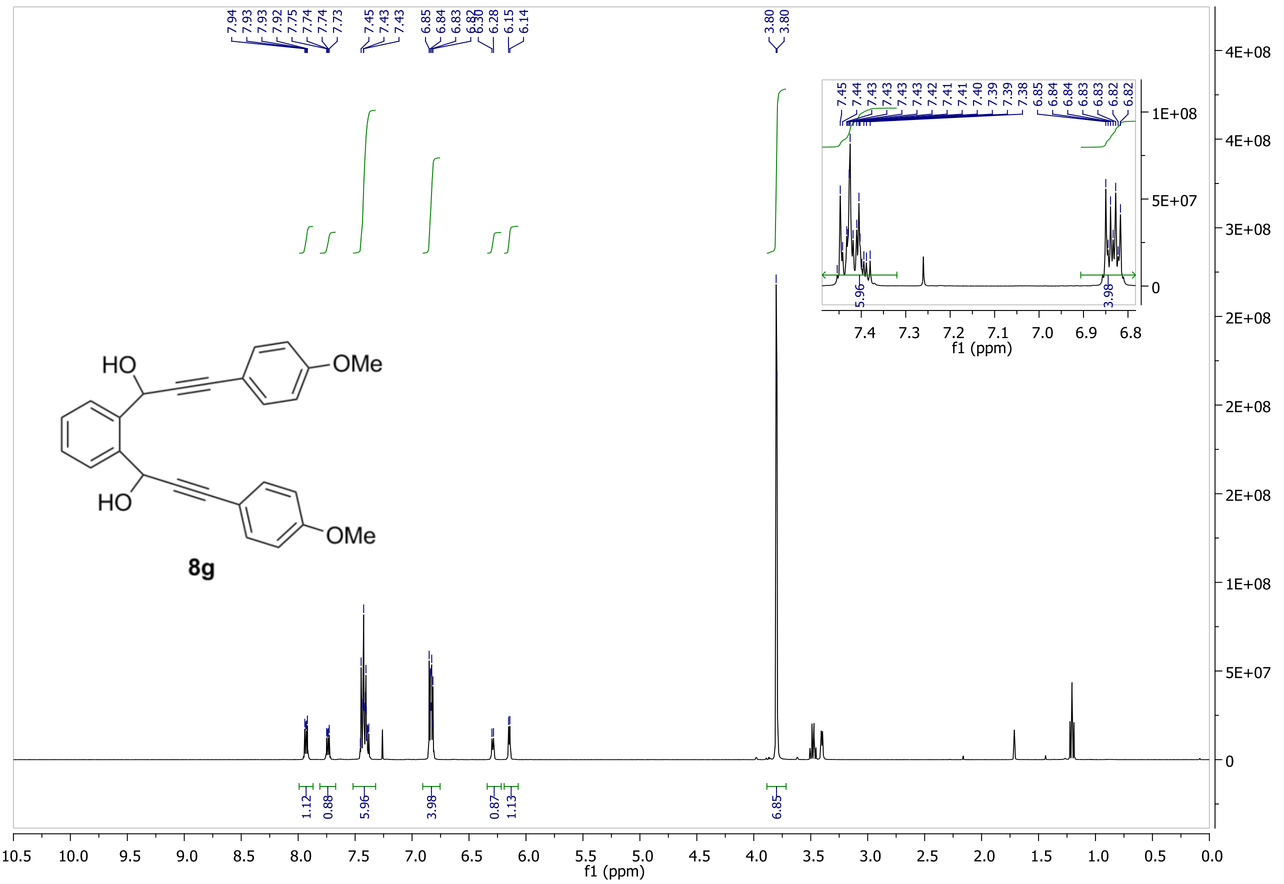
7.94 7.93 7.93 7.92 7.75 7.74 7.74 7.73 7.45 7.43 7.43 6.85 6.84 6.83 6.82 6.30 6.28 6.15 6.14 3.80 3.80
HO
OMe
HO
OMe
8g
1.12 0.88 5.96 3.98 0.87 1.13 6.85
7.4 7.3 7.2 7.1 7.0 6.9 6.8
f1 (ppm)
10.5 10.0 9.5 9.0 8.5 8.0 7.5 7.0 6.5 6.0 5.5 5.0 4.5 4.0 3.5 3.0 2.5 2.0 1.5 1.0 0.5 0.0
f1 (ppm)

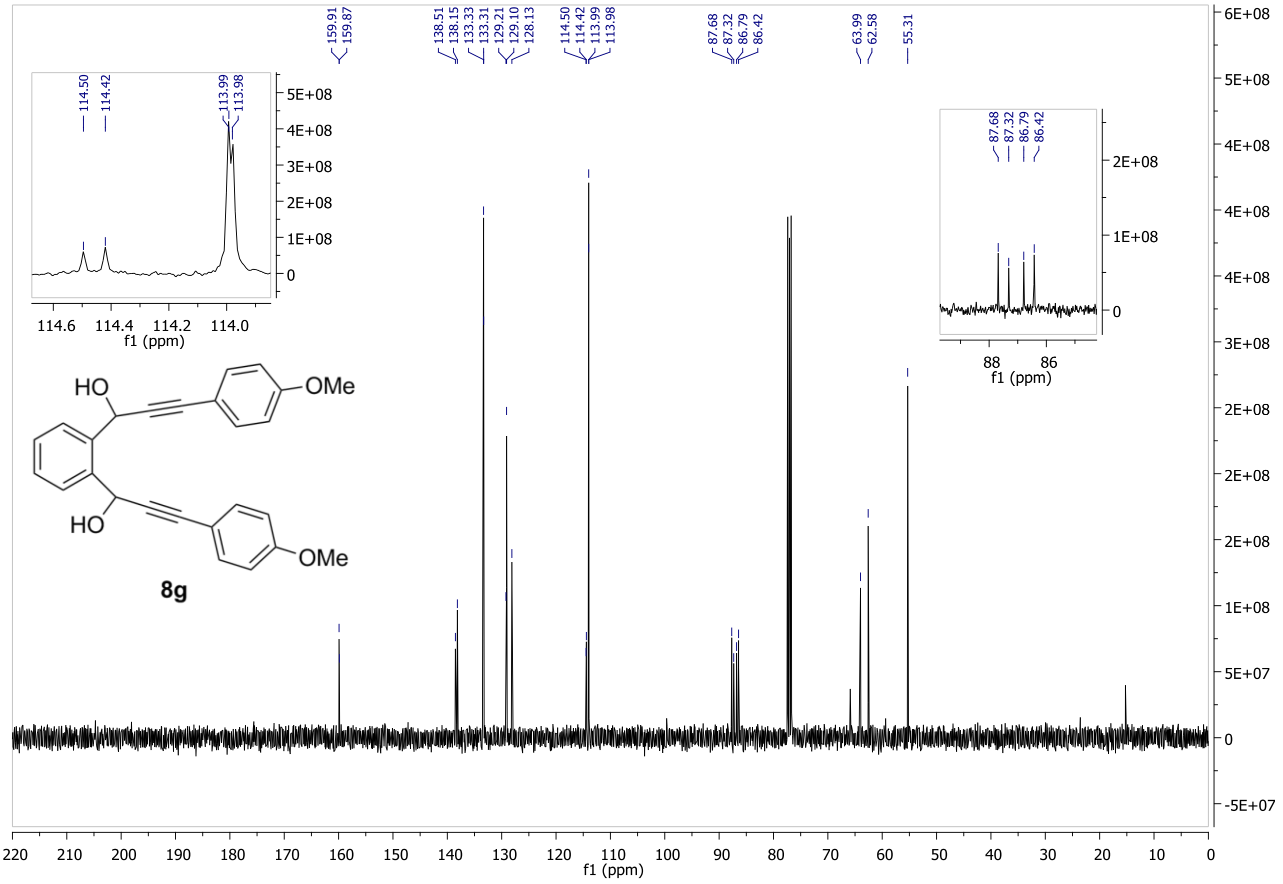
159.91 159.87 138.51 138.15 133.33 133.31 129.21 129.10 128.13 114.50 114.42 113.99 113.98 87.68 87.32 86.79 86.42 63.99 62.58 55.31
114.6 114.4 114.2 114.0
f1 (ppm)
88 86
f1 (ppm)
HO
OMe
HO
OMe
8g
220 210 200 190 180 170 160 150 140 130 120 110 100 90 80 70 60 50 40 30 20 10 0
f1 (ppm)

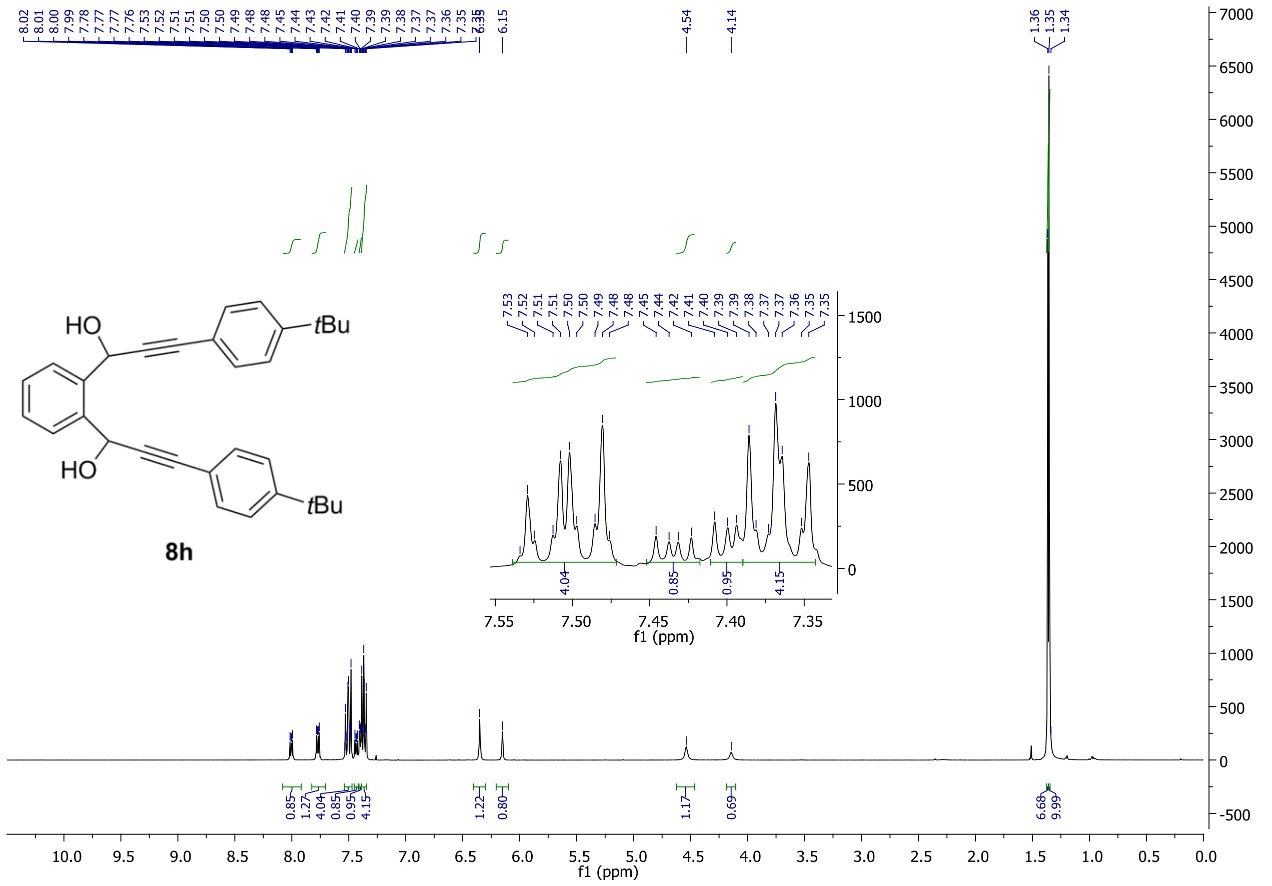

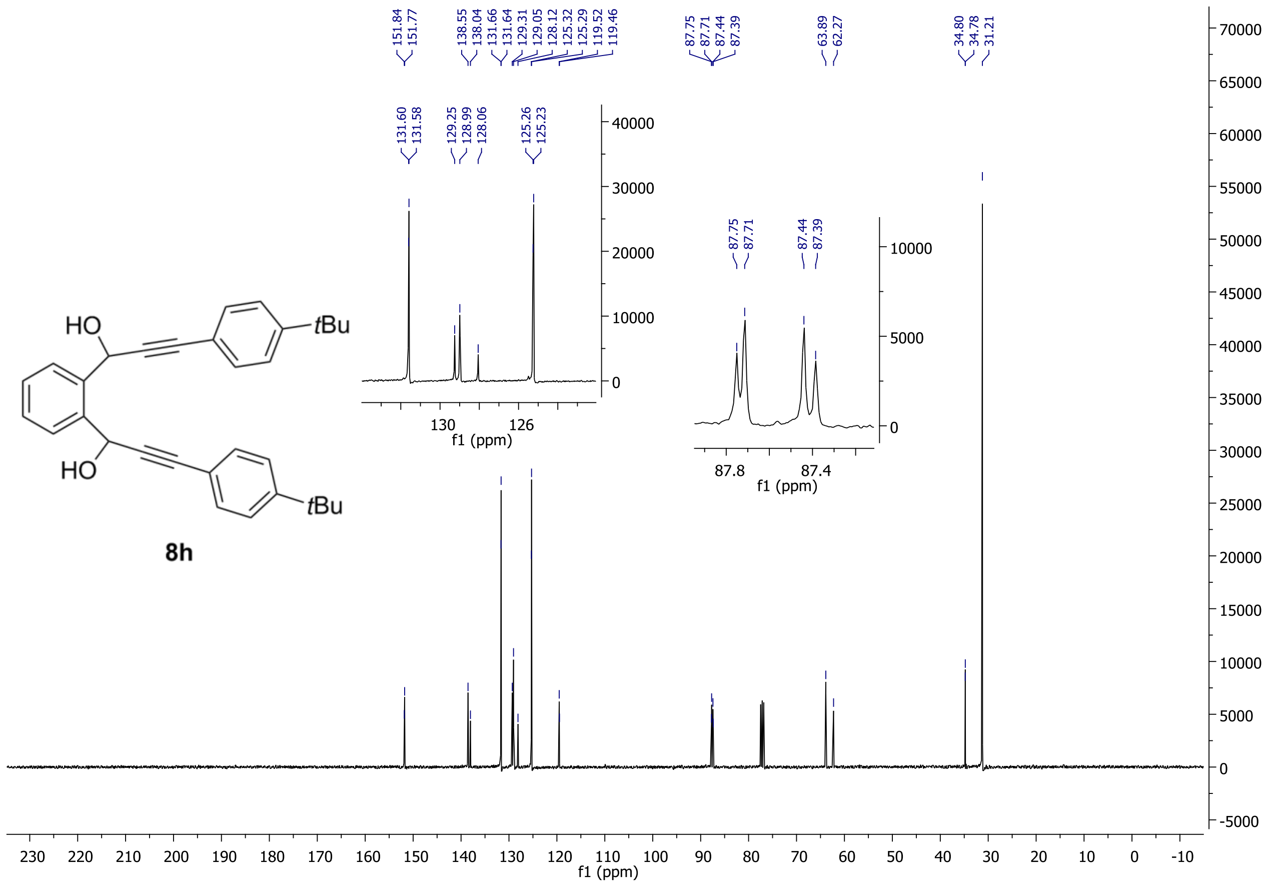

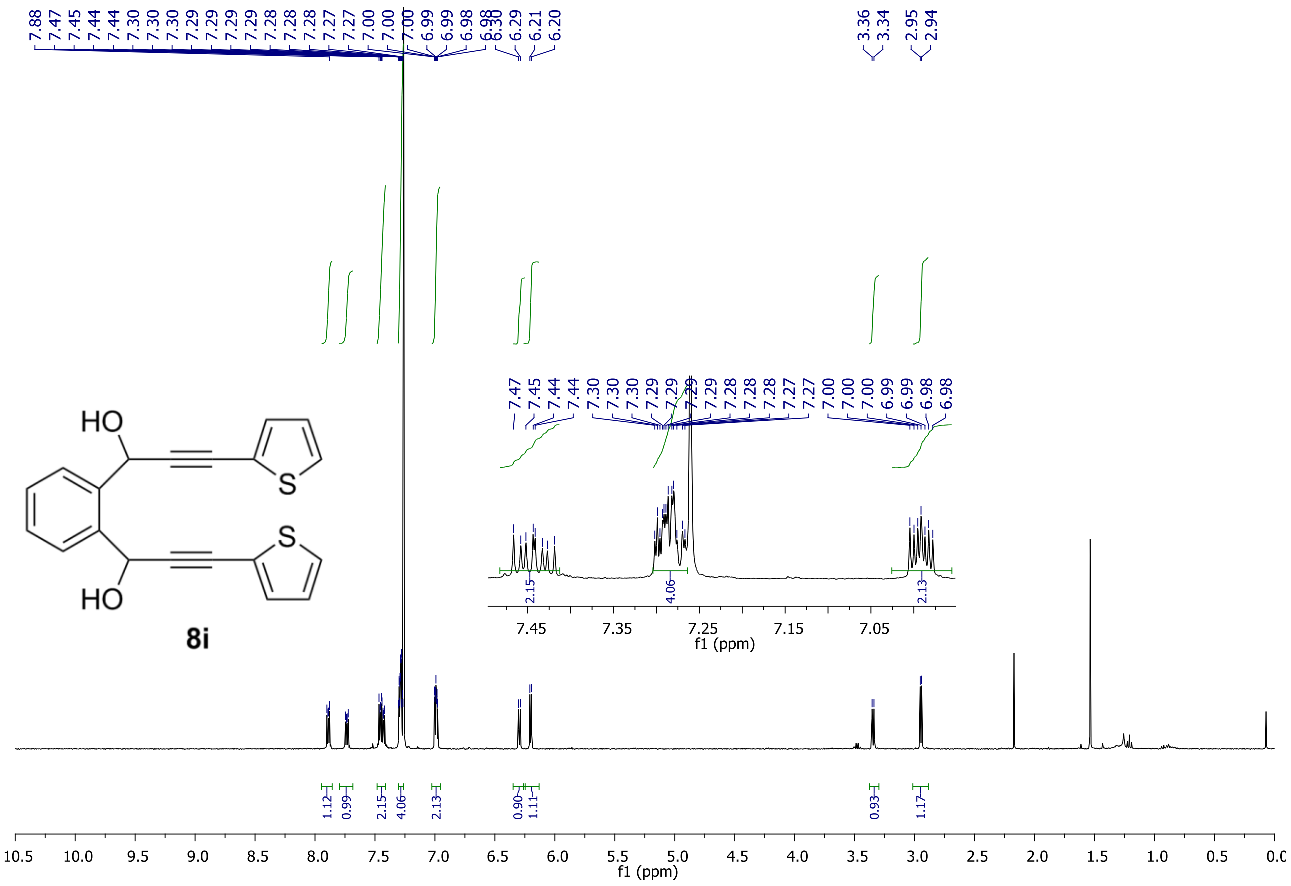
HO
S
S
HO
8i
7.45
7.35
7.25
7.15
7.05
f1 (ppm)
10.5
10.0
9.5
9.0
8.5
8.0
7.5
7.0
6.5
6.0
5.5
5.0
4.5
4.0
3.5
3.0
2.5
2.0
1.5
1.0
0.5
0.0
f1 (ppm)

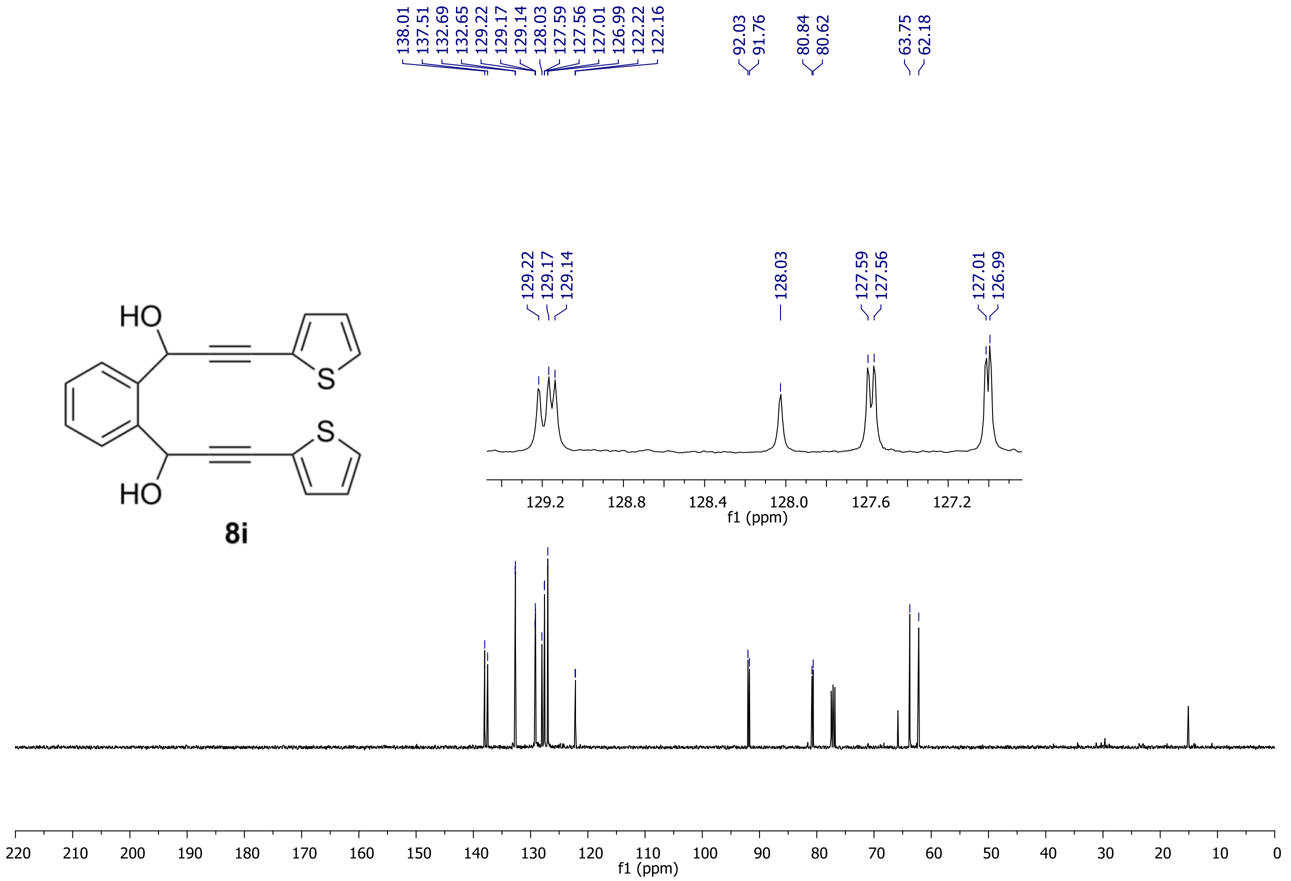
138.01
137.51
132.69
132.65
129.22
129.17
129.14
128.03
127.59
127.56
127.01
126.99
122.22
122.16
92.03
91.76
80.84
80.62
63.75
62.18
HO
S
S
HO
8i
129.2
128.8
128.4
128.0
127.6
127.2
f1 (ppm)
220
210
200
190
180
170
160
150
140
130
120
110
100
90
80
70
60
50
40
30
20
10
0
f1 (ppm)

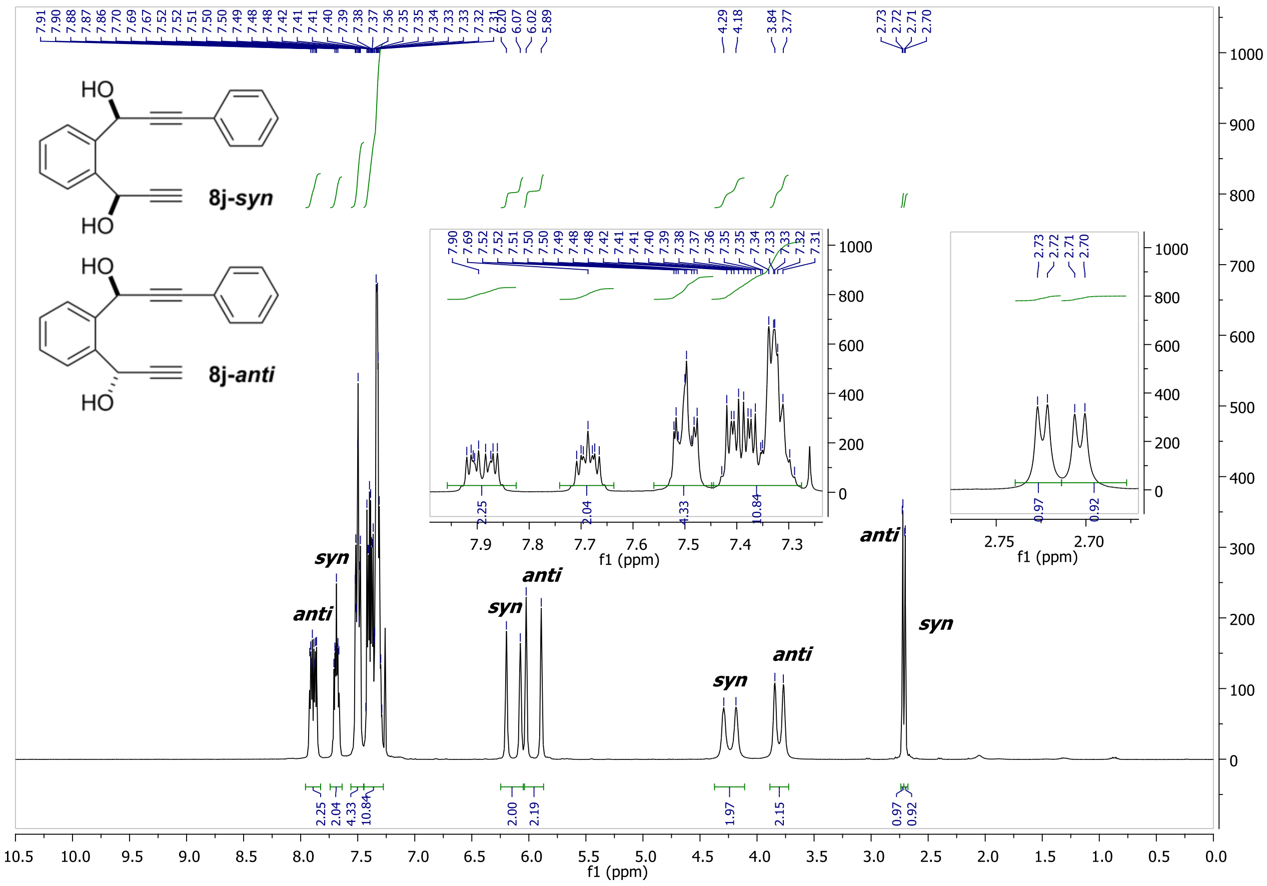
HO
HO
8j-syn
HO
HO
8j-anti
anti
syn
syn
anti
anti
syn
syn
anti
f1 (ppm)

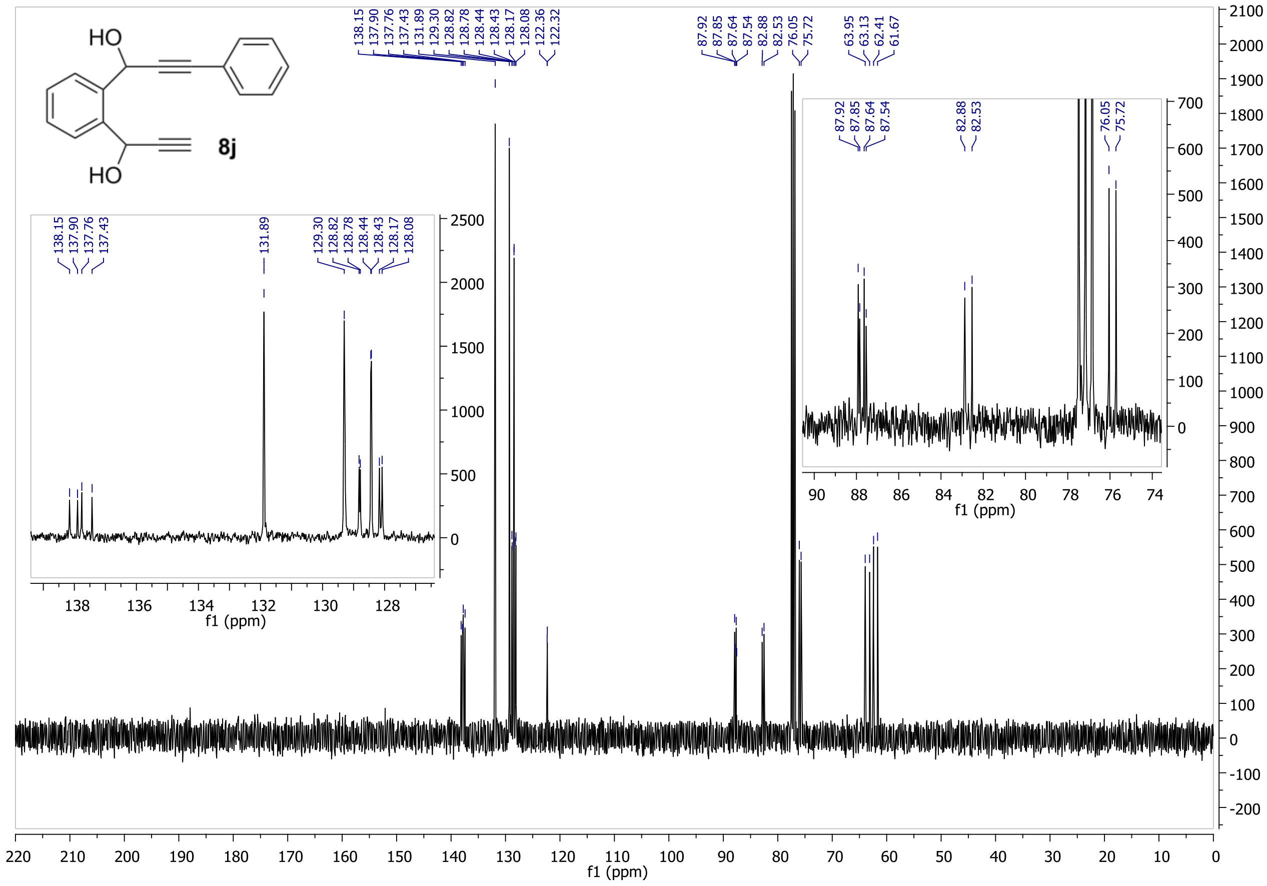
HO
HO
8j
f1 (ppm)

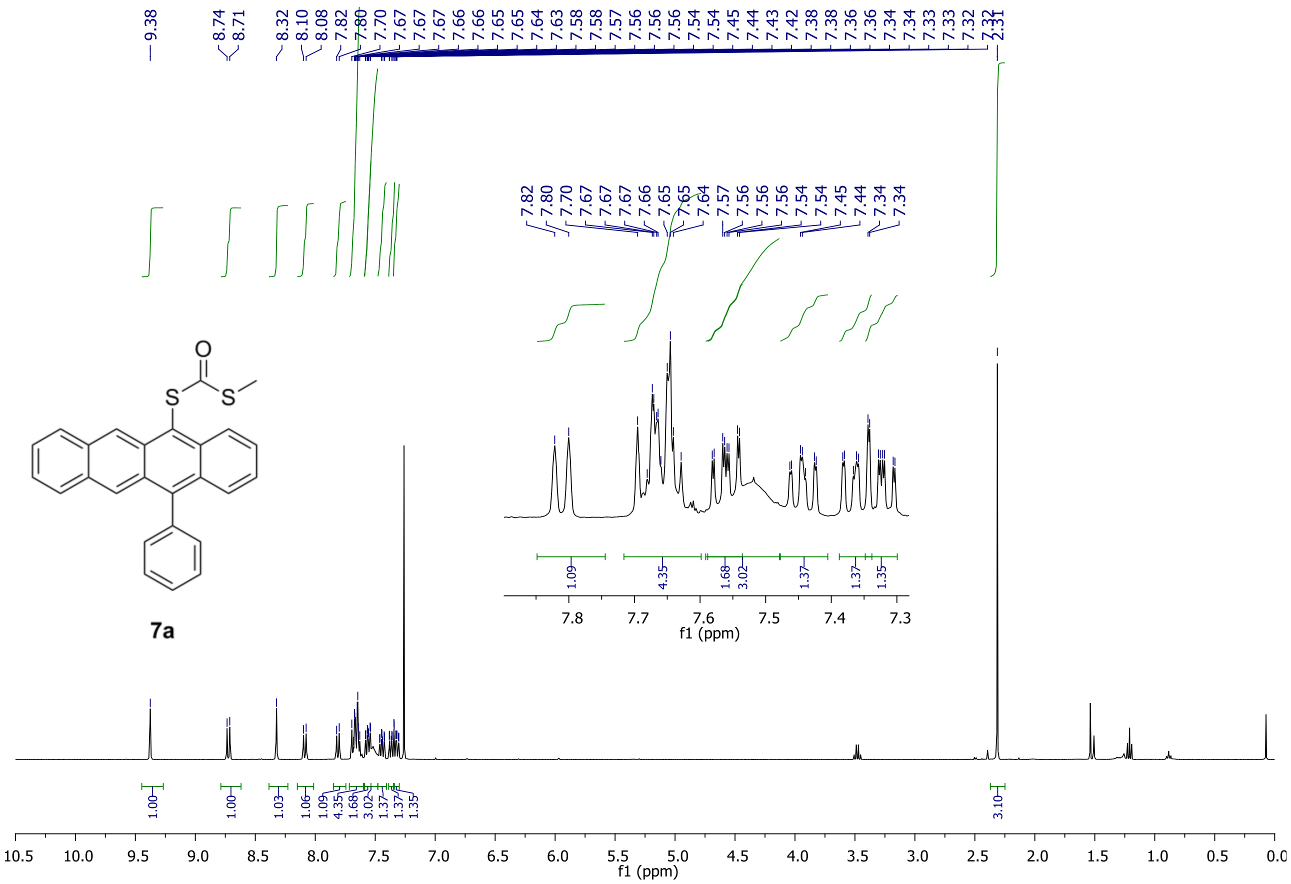
9.38
8.74
8.71
8.32
8.10
8.08
7.82
7.80
7.70
7.67
7.67
7.66
7.66
7.65
7.65
7.64
7.63
7.58
7.58
7.57
7.56
7.56
7.56
7.54
7.54
7.45
7.44
7.43
7.42
7.38
7.38
7.36
7.36
7.34
7.34
7.33
7.33
7.32
7.32
2.31
1.00
1.00
1.03
1.06
1.09
4.35
1.68
3.02
1.37
1.37
1.35
3.10
O
S
S
7a
f1 (ppm)

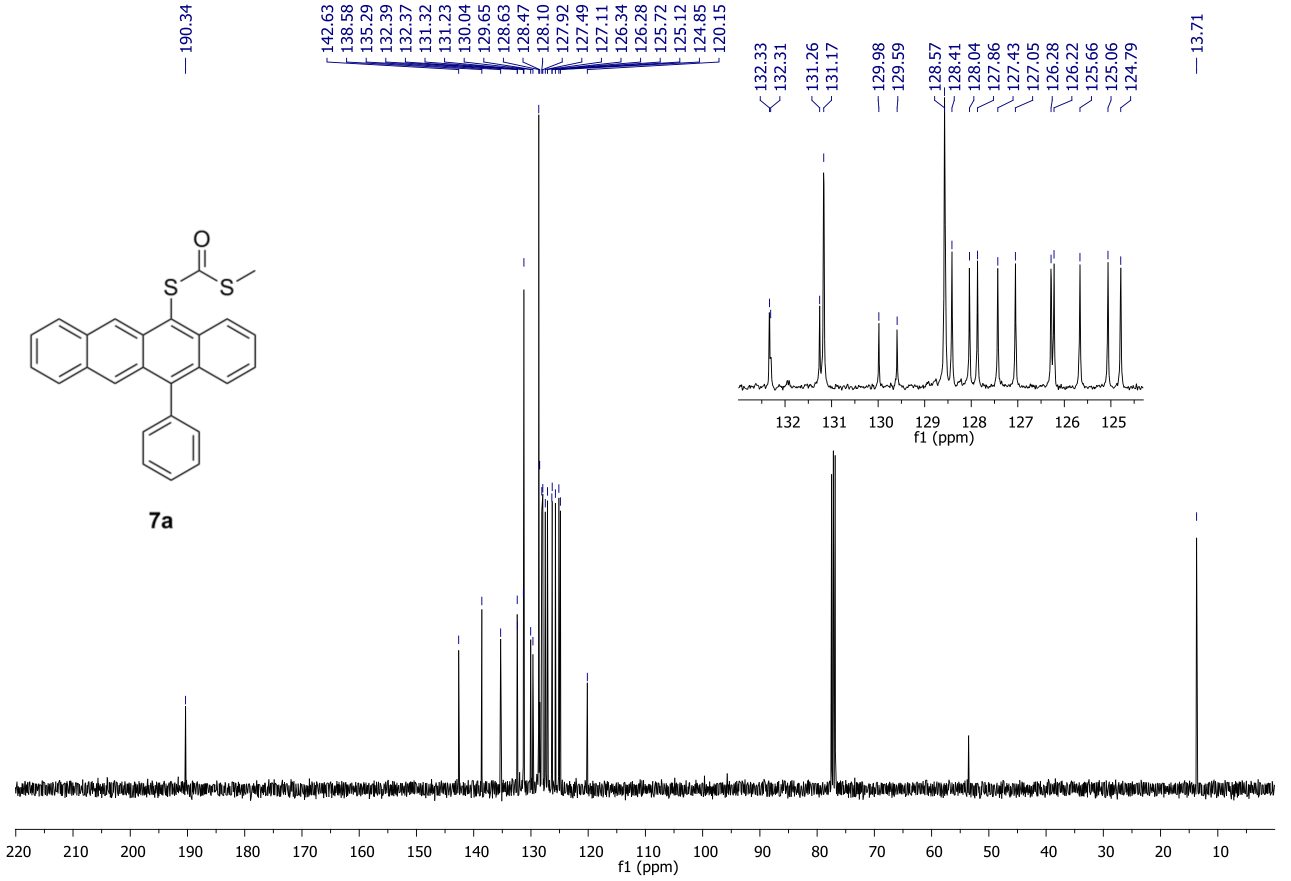
190.34
142.63
138.58
135.29
132.39
132.37
131.32
131.23
130.04
129.65
128.63
128.47
128.10
127.92
127.49
127.11
126.34
126.28
125.72
125.12
124.85
120.15
132.33
132.31
131.26
131.17
129.98
129.59
128.57
128.41
128.04
127.86
127.43
127.05
126.28
126.22
125.66
125.06
124.79
13.71
O
S
S
7a
f1 (ppm)

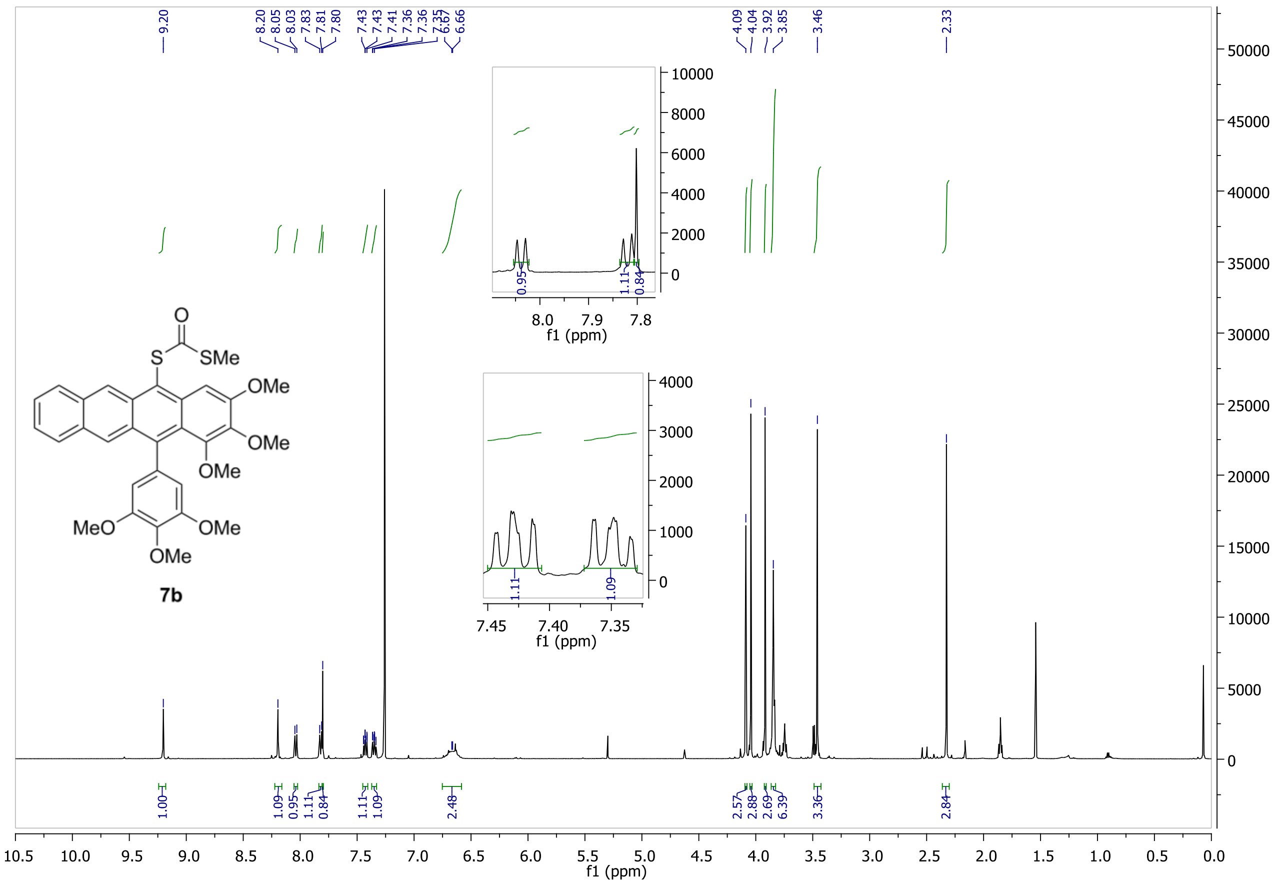

9.20
8.20
8.05
8.03
7.83
7.81
7.80
7.43
7.43
7.41
7.36
7.36
7.35
6.67
6.66
4.09
4.04
3.92
3.85
3.46
2.33
S
SMe
O
OMe
OMe
OMe
MeO
OMe
OMe
7b
f1 (ppm)

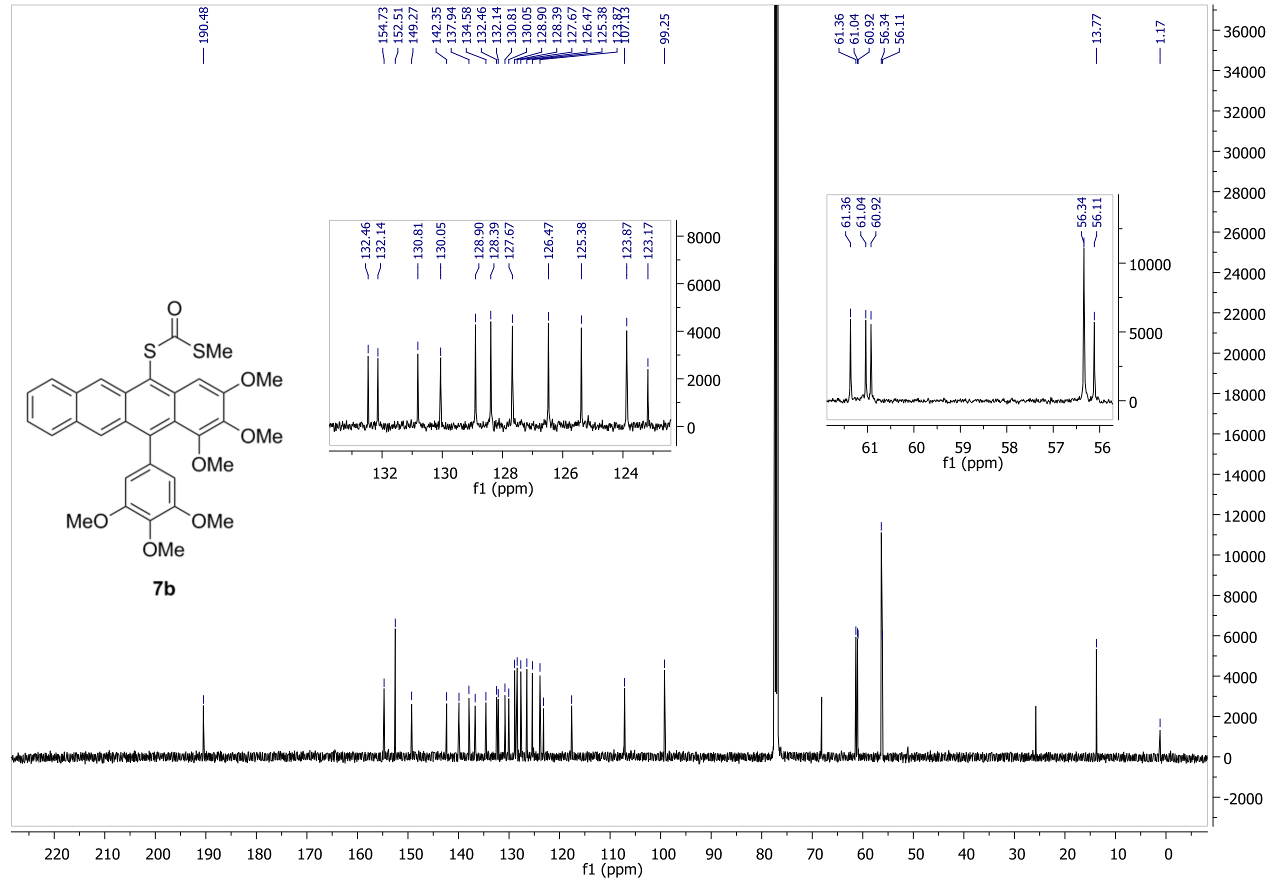

190.48
154.73
152.51
149.27
142.35
137.94
134.58
132.46
132.14
130.81
130.05
128.90
128.39
127.67
126.47
125.38
123.87
123.17
107.13
99.25
61.36
61.04
60.92
56.34
56.11
13.77
1.17
S
SMe
O
OMe
OMe
OMe
MeO
OMe
OMe
7b
f1 (ppm)

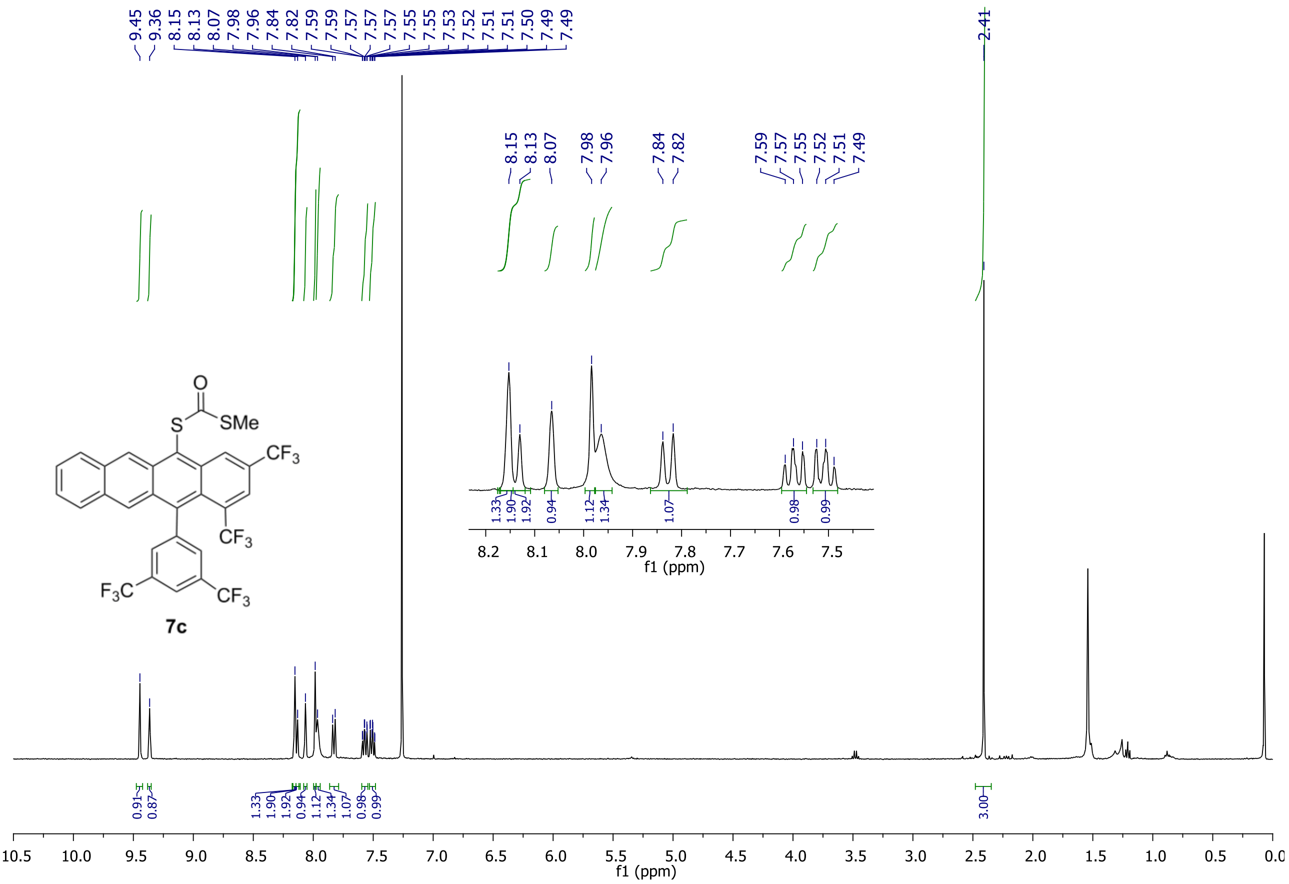

9.45
9.36
8.15
8.13
8.07
7.98
7.96
7.84
7.82
7.59
7.59
7.57
7.57
7.57
7.55
7.55
7.53
7.52
7.51
7.51
7.50
7.49
7.49
2.41
7c
f1 (ppm)

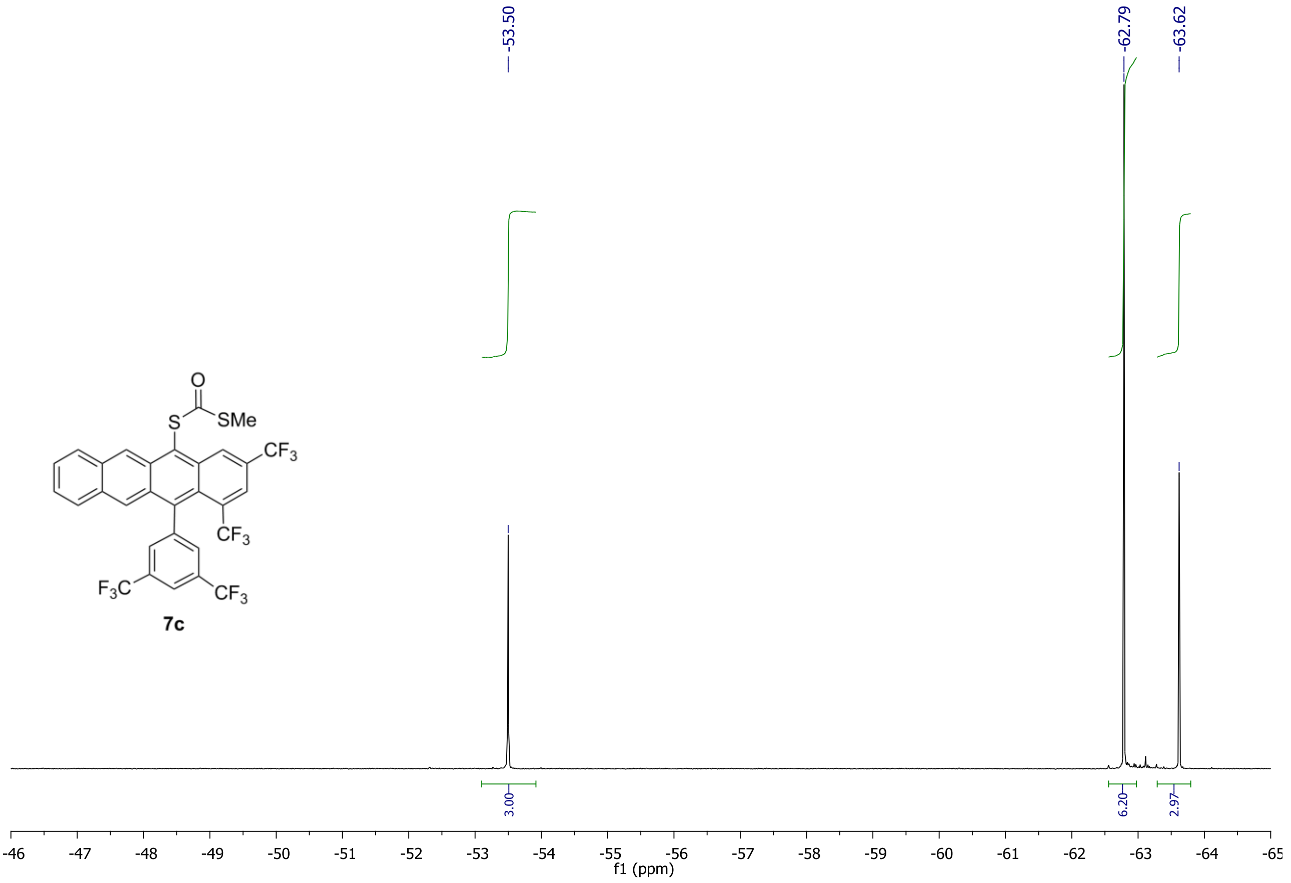

-53.50
-62.79
-63.62
7c
3.00
6.20
2.97
f1 (ppm)

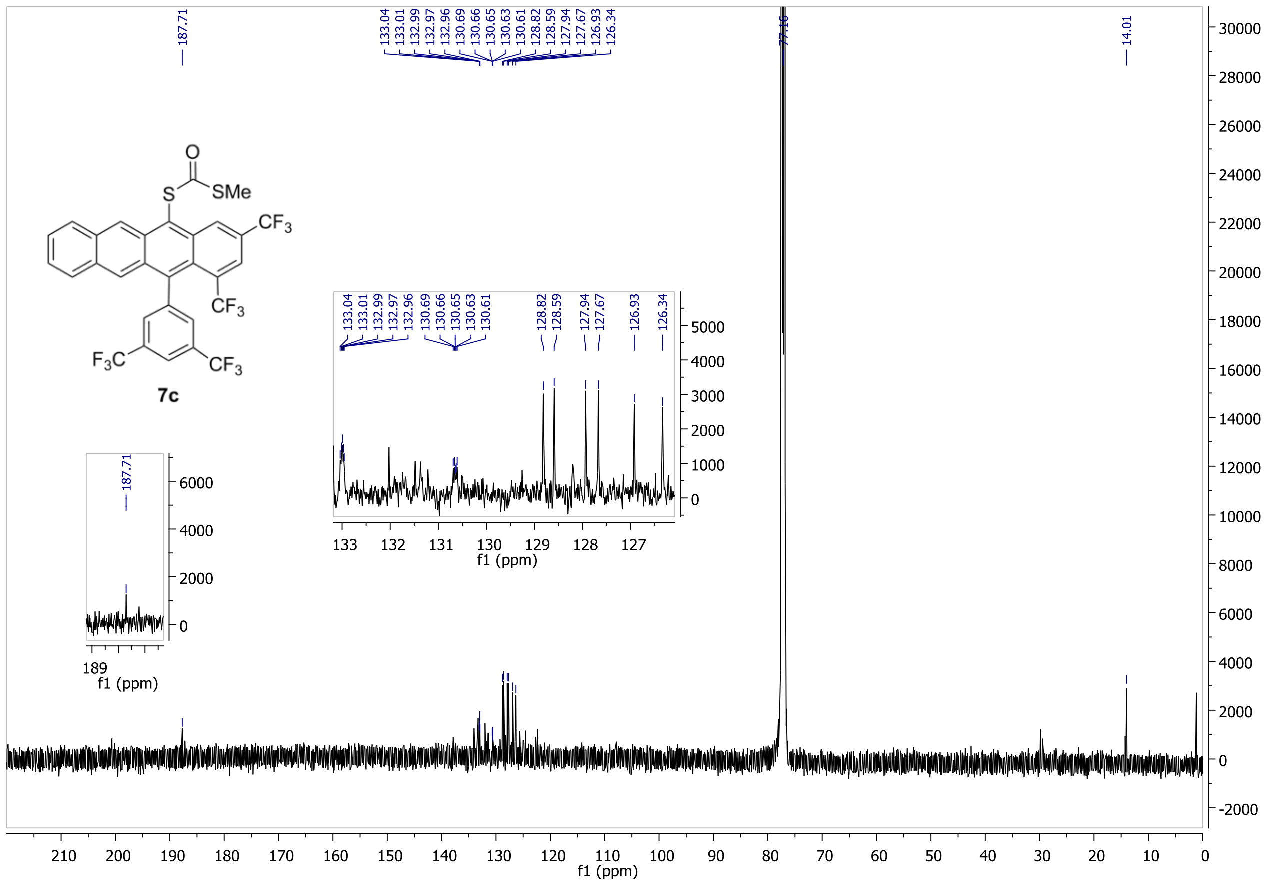

S
O
SMe
CF3
CF3
F3C
CF3
7c
f1 (ppm)

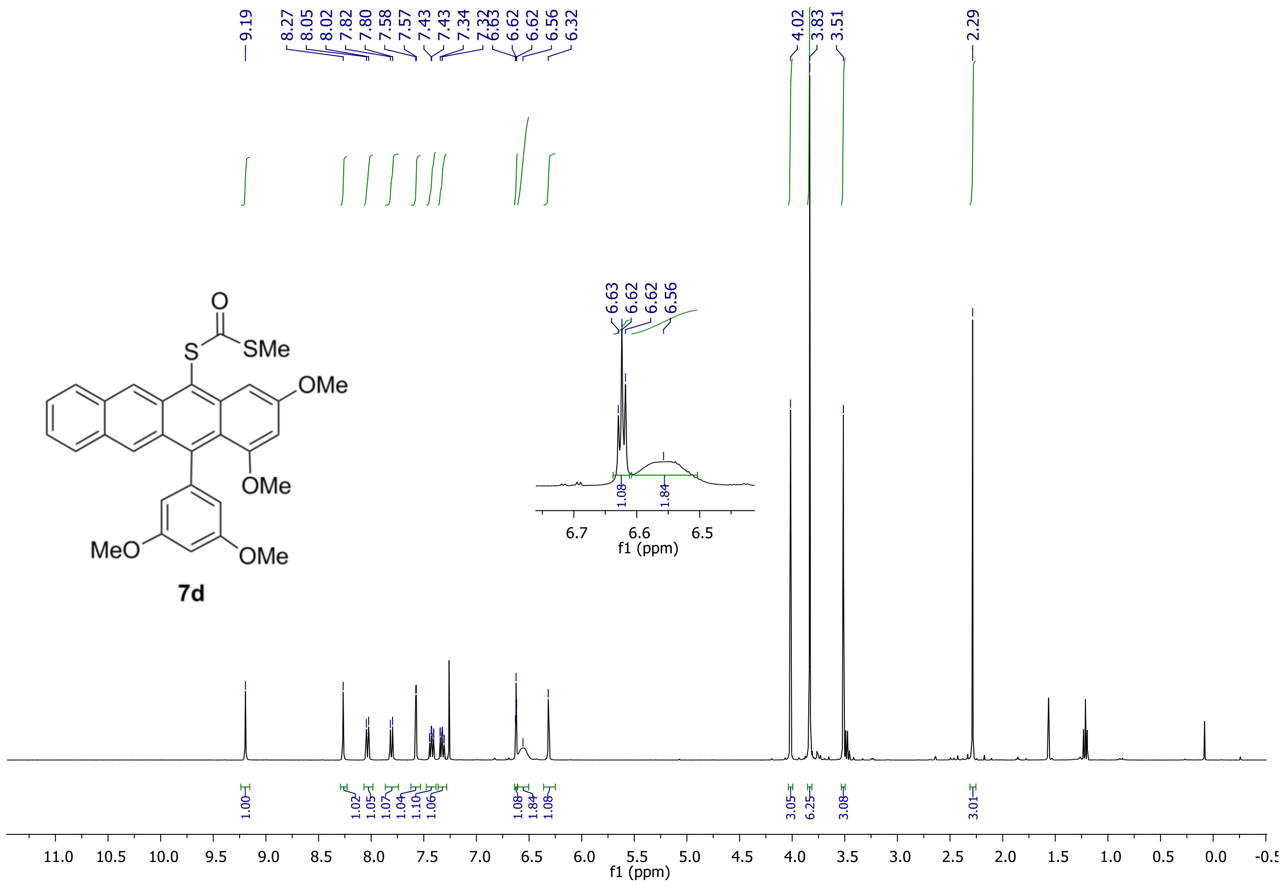

S
O
SMe
OMe
OMe
MeO
OMe
7d
f1 (ppm)

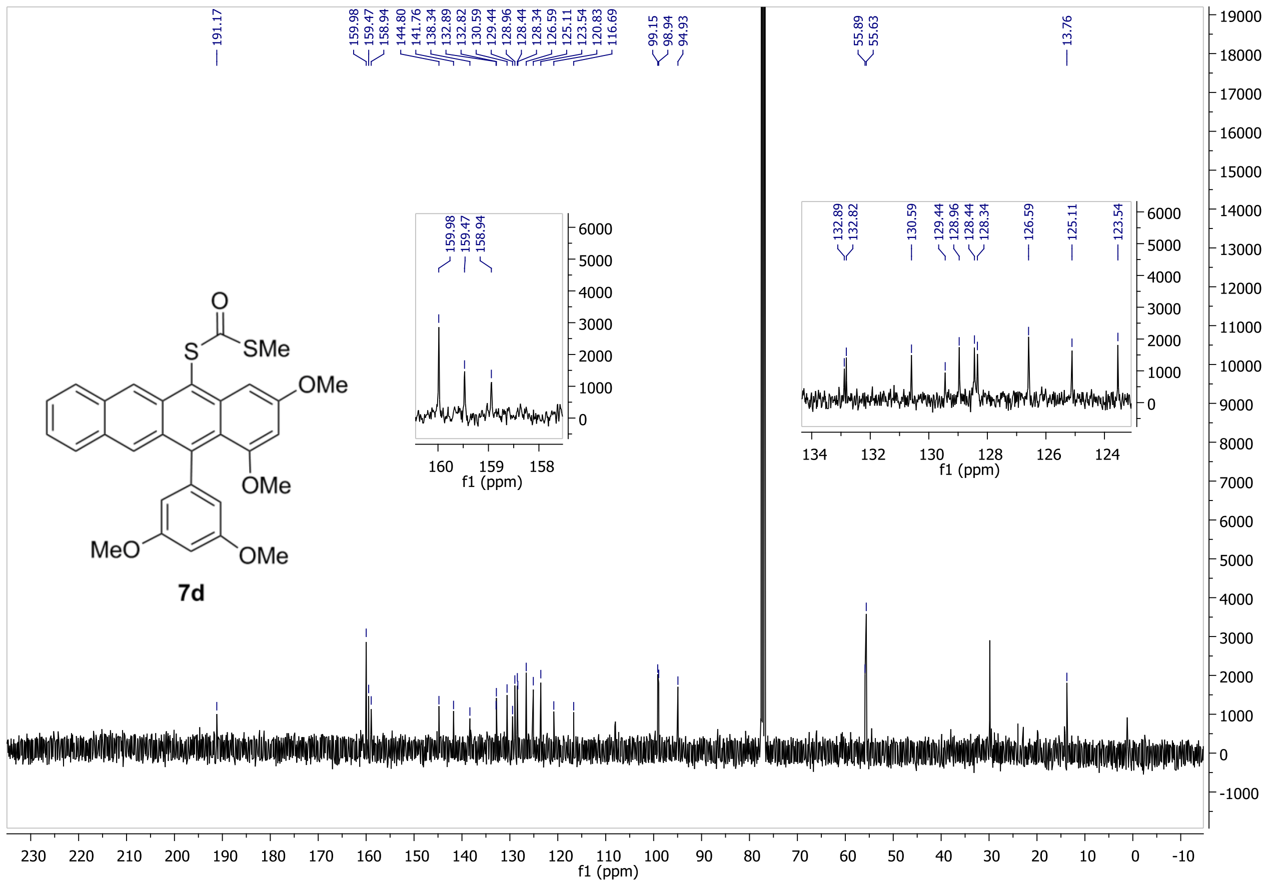

7d
SMe
OMe
MeO
f1 (ppm)

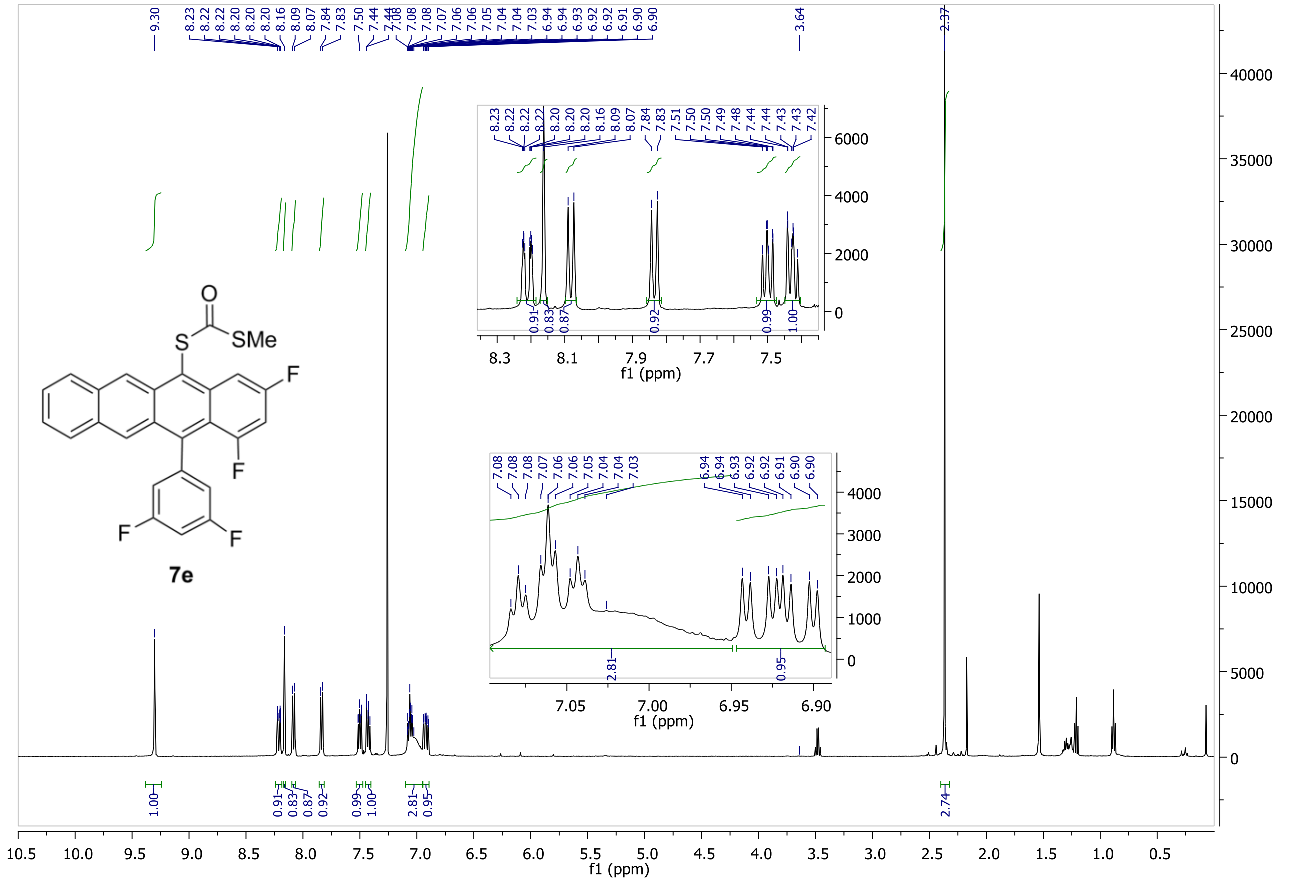

7e
SMe
F
f1 (ppm)

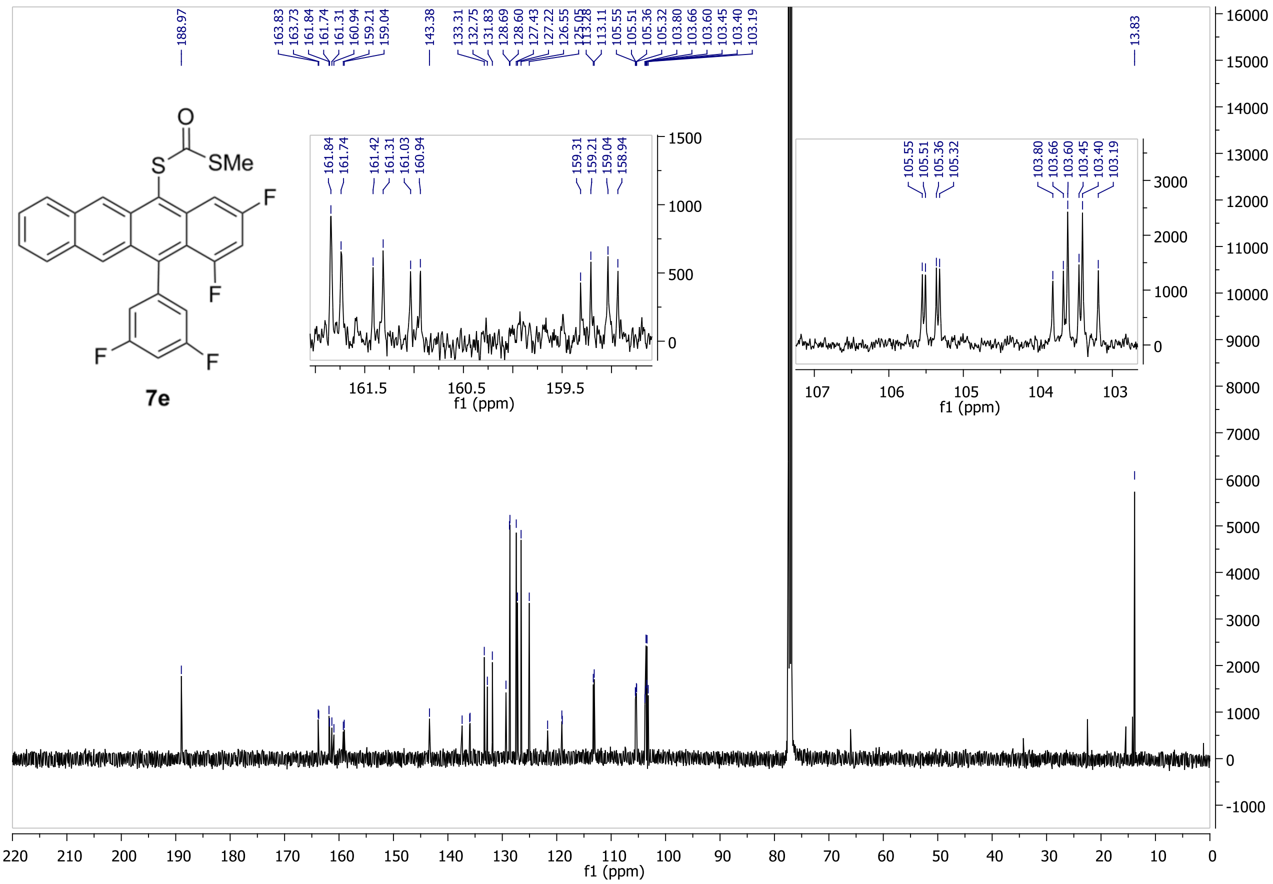
188.97
163.83
163.73
161.84
161.74
161.31
160.94
159.21
159.04
143.38
133.31
132.75
131.83
128.69
128.60
127.43
127.22
126.55
113.11
105.55
105.51
105.36
105.32
103.80
103.66
103.60
103.45
103.40
103.19
13.83
O
S
SMe
F
F
F
F
7e
f1 (ppm)

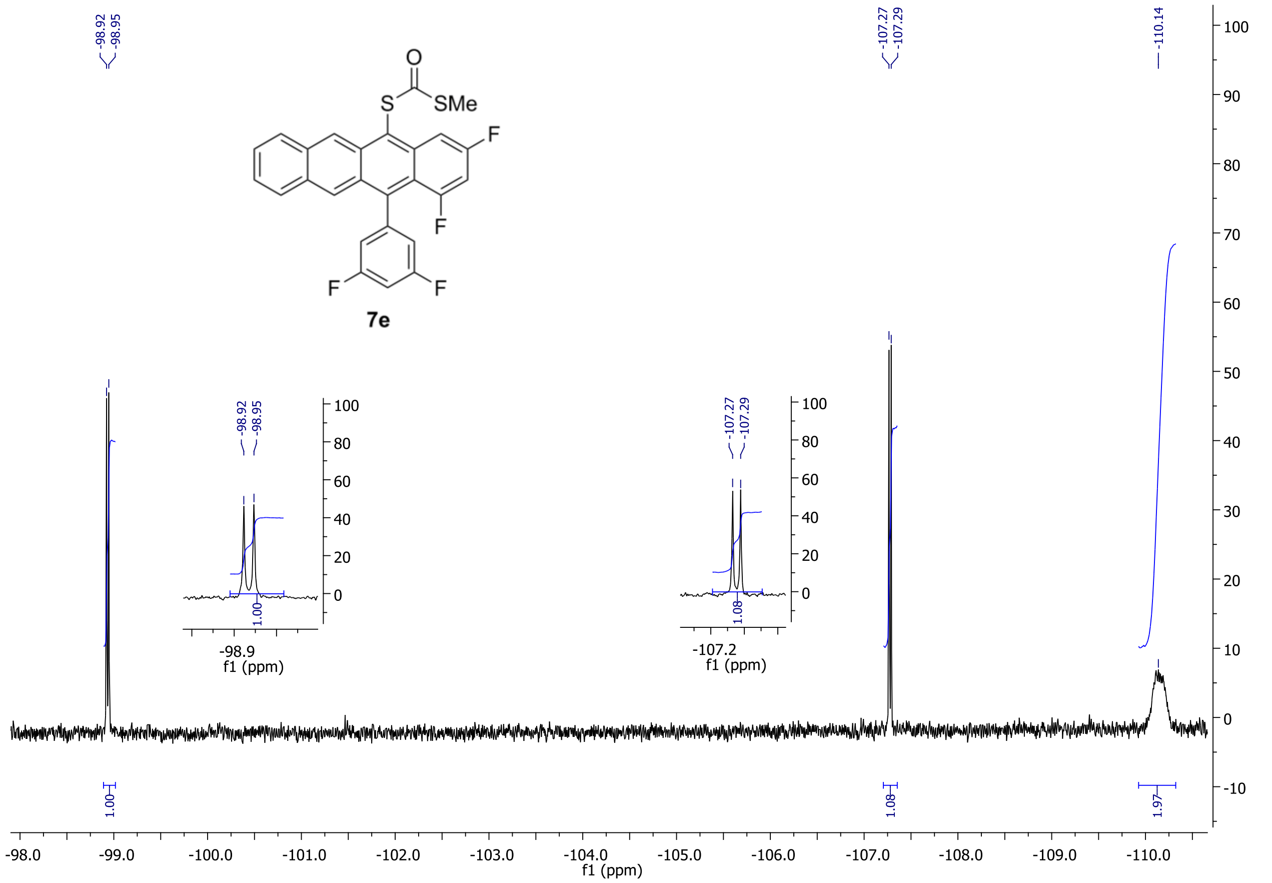
-98.92
-98.95
-107.27
-107.29
-110.14
O
S
SMe
F
F
F
F
7e
1.00
1.08
1.97
f1 (ppm)

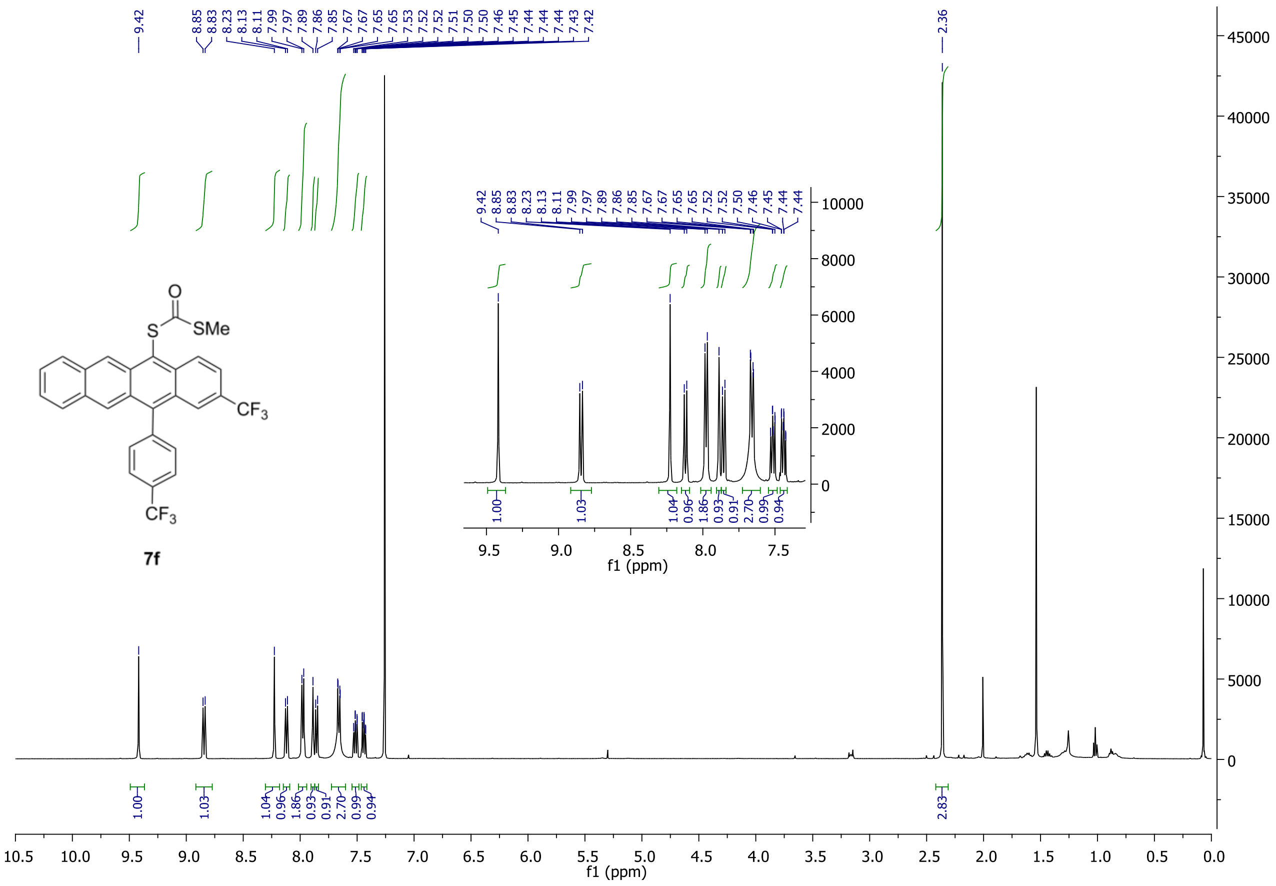
SMe
CF3
7f
f1 (ppm)
A (s) -62.41
B (s) -63.25

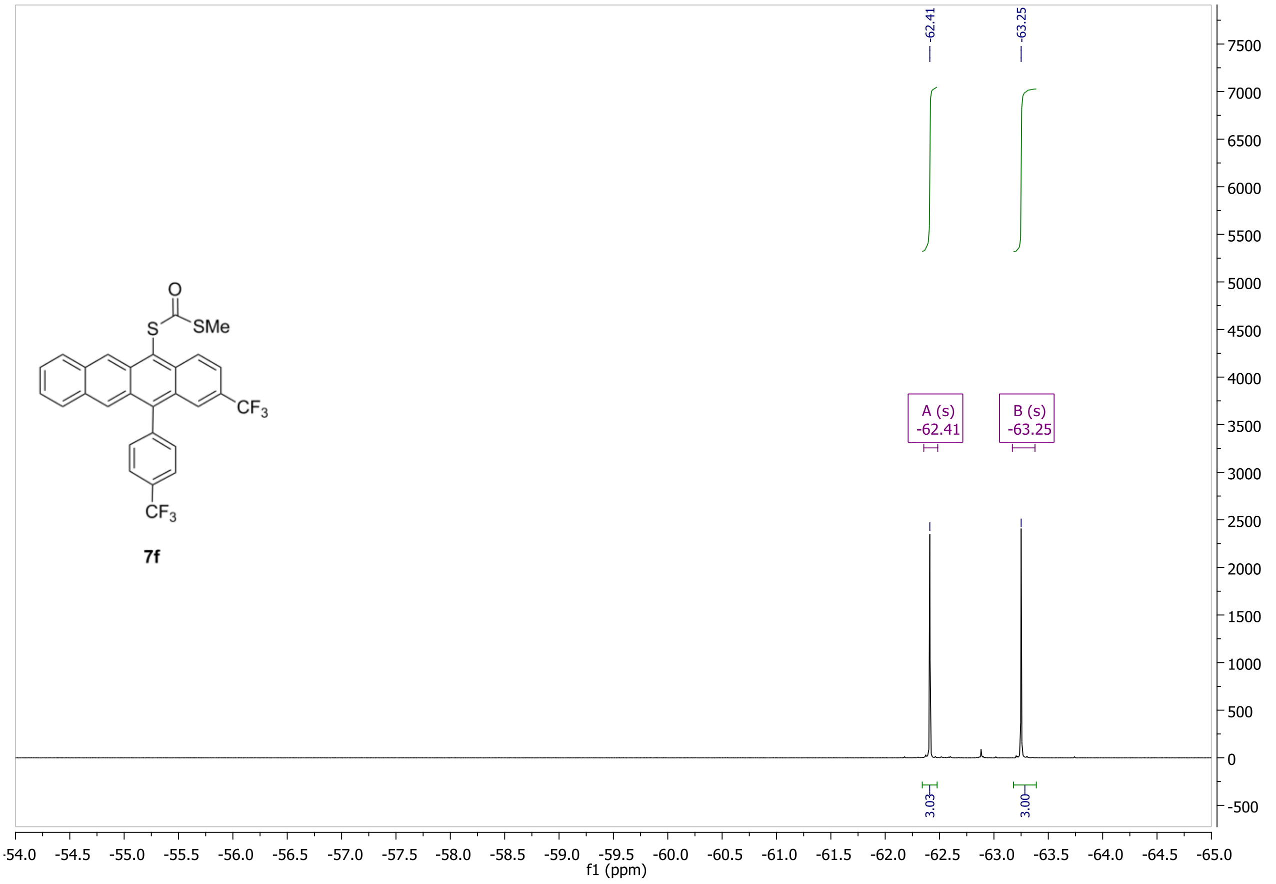
SMe
CF3
7f
A (s)
-62.41
B (s)
-63.25
f1 (ppm)

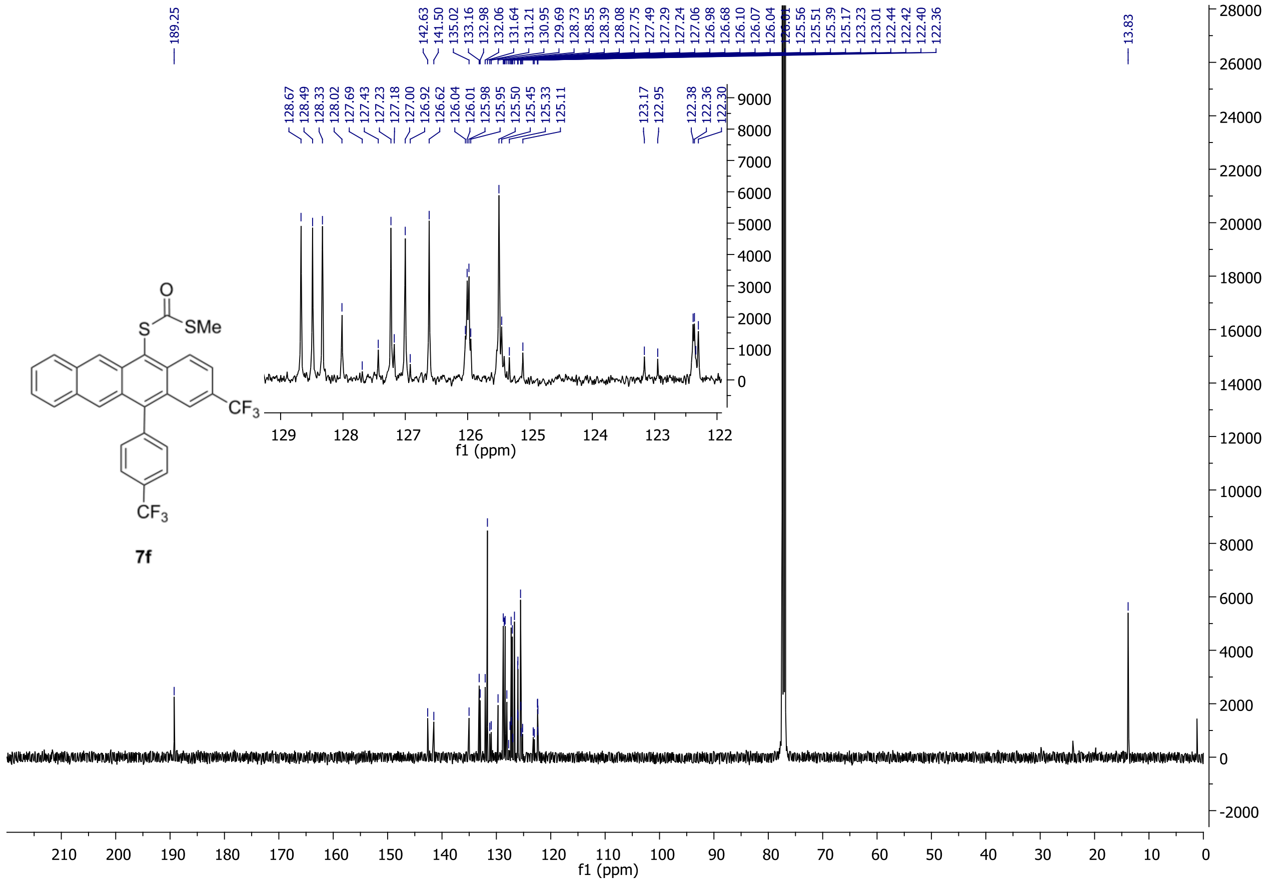

189.25
142.63 141.50 135.02 133.16 132.98 132.06 131.64 131.21 130.95 129.69 128.73 128.55 128.39 128.08 127.75 127.49 127.29 127.24 127.06 126.98 126.68 126.10 126.07 126.04 125.56 125.51 125.39 125.17 123.23 123.01 122.44 122.42 122.40 122.36
13.83
128.67 128.49 128.33 128.02 127.69 127.43 127.23 127.18 127.00 126.92 126.62 126.04 126.01 125.98 125.95 125.50 125.45 125.33 125.11
123.17 122.95
122.38 122.36 122.30
O
S
SMe
CF3
CF3
7f
f1 (ppm)

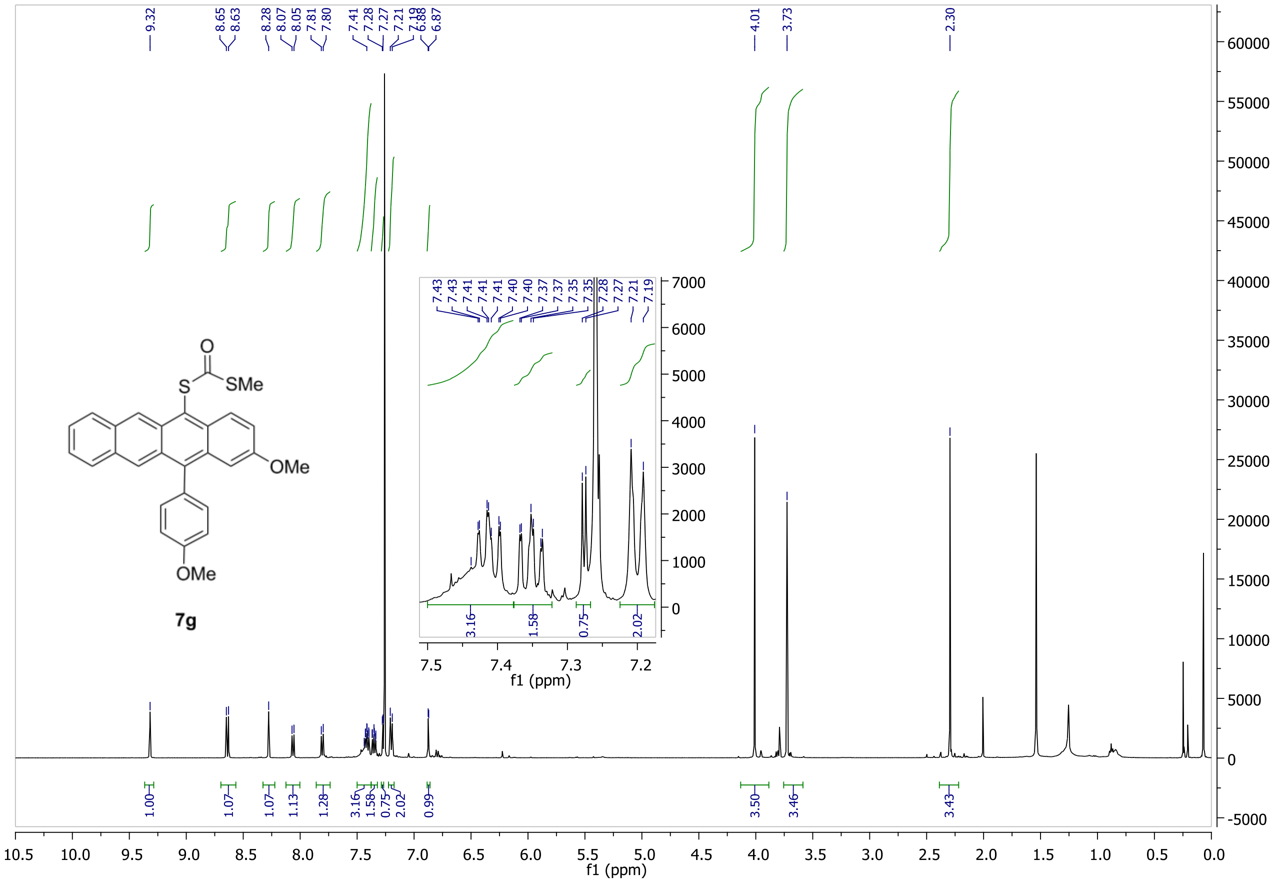

9.32
8.65 8.63
8.28 8.07 8.05 7.81 7.80
7.41 7.28 7.27 7.21 7.19 6.88 6.87
4.01 3.73 2.30
7.43 7.43 7.41 7.41 7.40 7.37 7.37 7.35 7.28 7.27 7.21 7.19
3.16 1.58 0.75 2.02
1.00 1.07 1.07 1.13 1.28 3.16 1.58 0.75 2.02 0.99 3.50 3.46 3.43
O
S
SMe
OMe
OMe
7g
f1 (ppm)

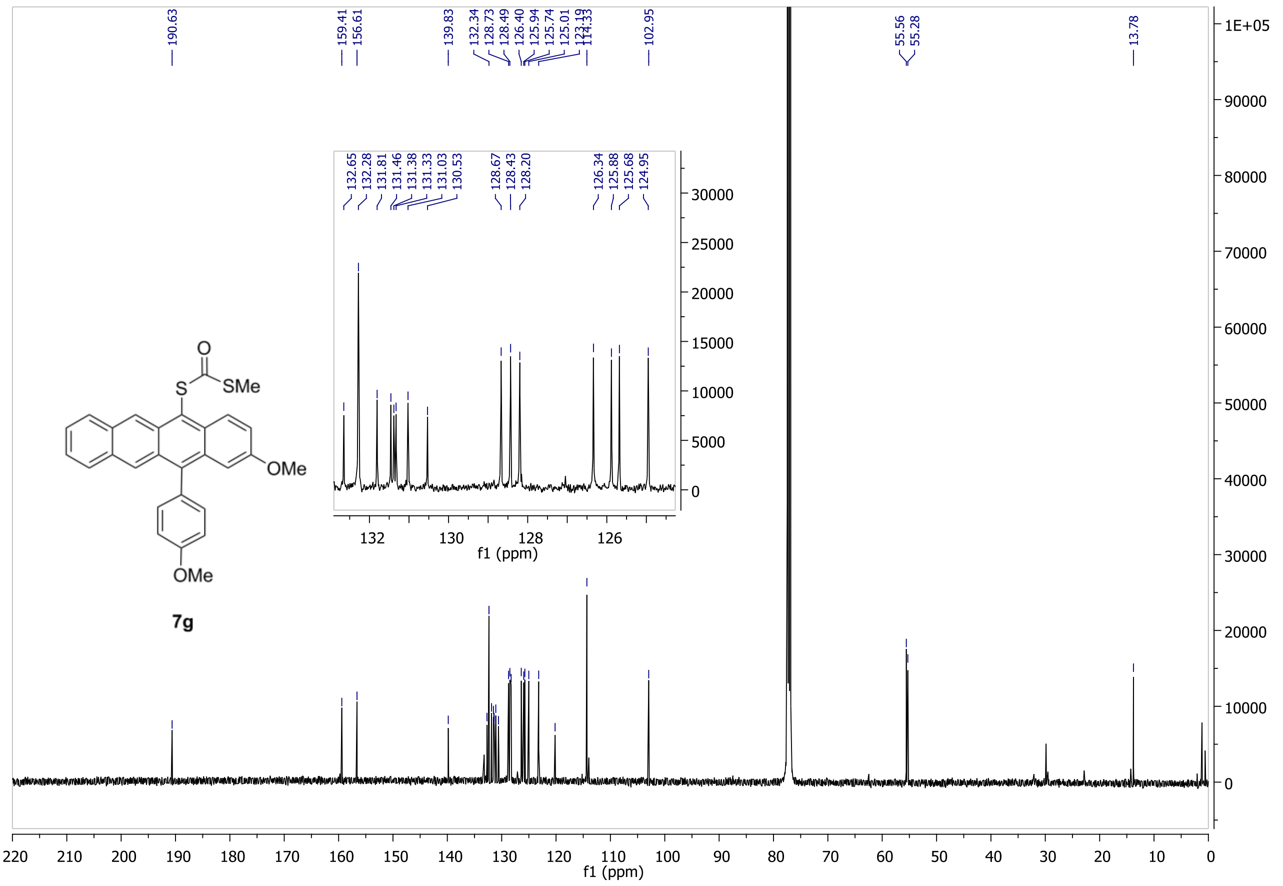
190.63
159.41
156.61
139.83
132.34
128.73
128.49
126.40
125.94
125.74
125.01
123.19
114.33
102.95
55.56
55.28
13.78
132.65
132.28
131.81
131.46
131.38
131.33
131.03
130.53
128.67
128.43
128.20
126.34
125.88
125.68
124.95
f1 (ppm)
O
S
SMe
OMe
OMe
7g

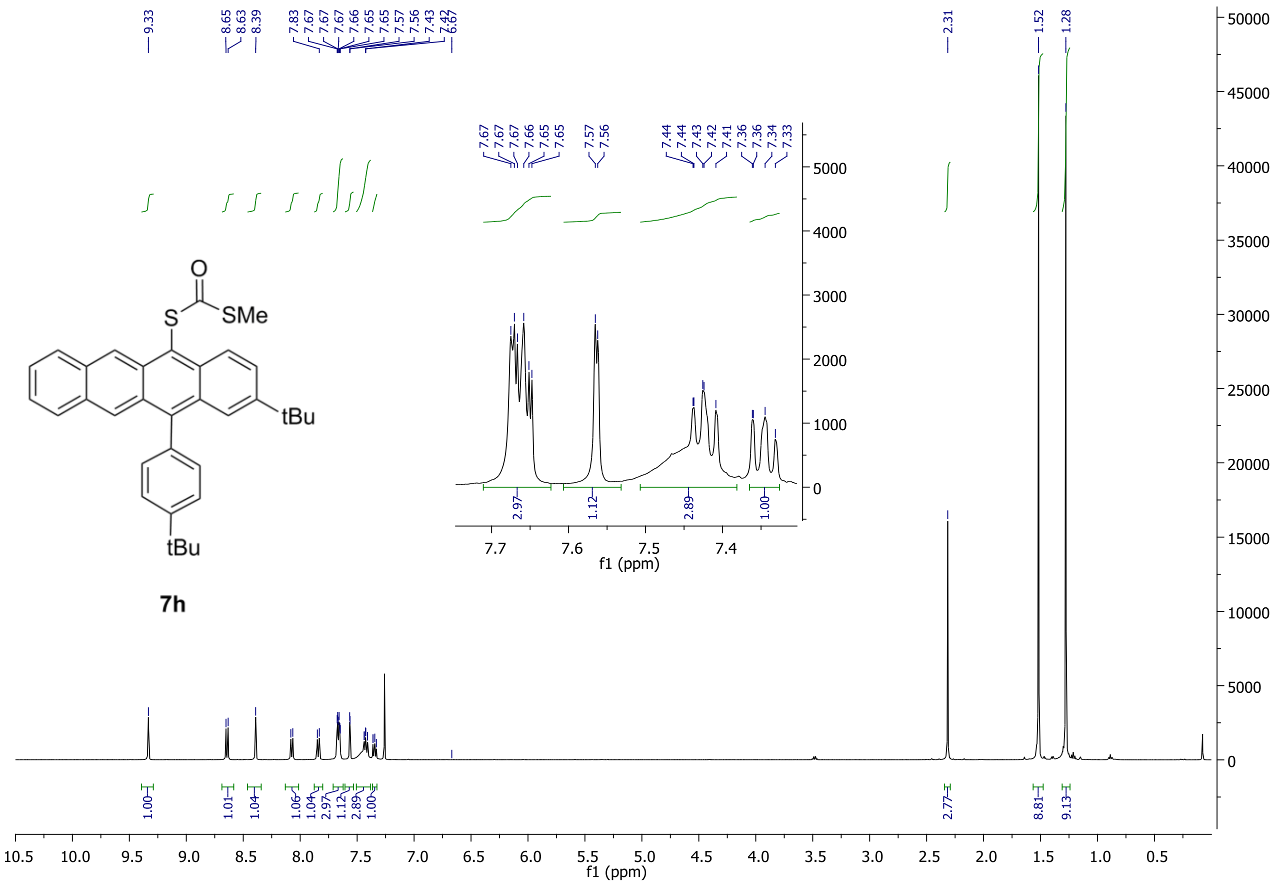
9.33
8.65
8.63
8.39
7.83
7.67
7.67
7.66
7.65
7.57
7.56
7.43
7.42
6.67
2.31
1.52
1.28
7.67
7.67
7.67
7.66
7.65
7.65
7.57
7.56
7.44
7.44
7.43
7.42
7.41
7.36
7.34
7.33
2.97
1.12
2.89
1.00
f1 (ppm)
O
S
SMe
tBu
tBu
7h
1.00
1.01
1.04
1.06
1.04
2.97
1.12
2.89
1.00
2.77
8.81
9.13

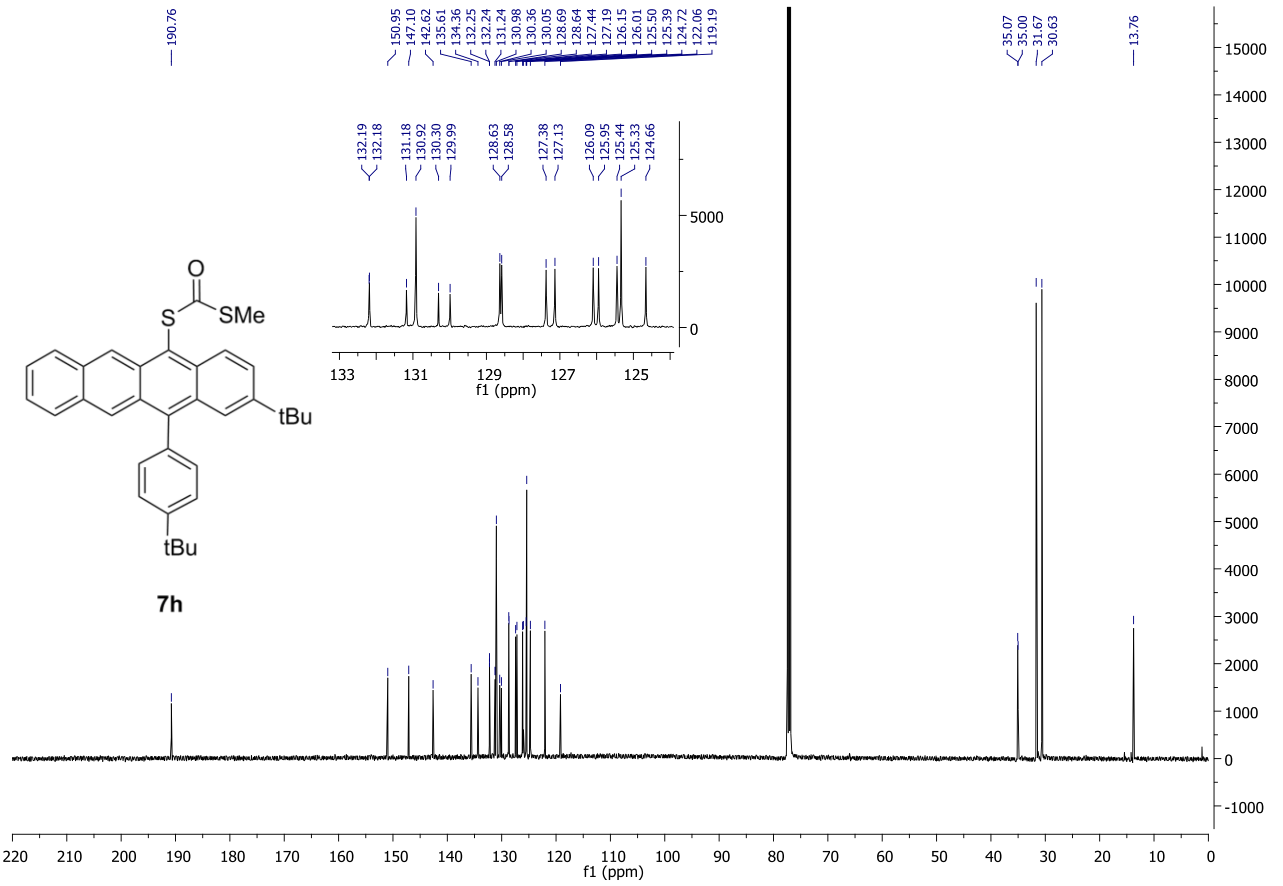

7h

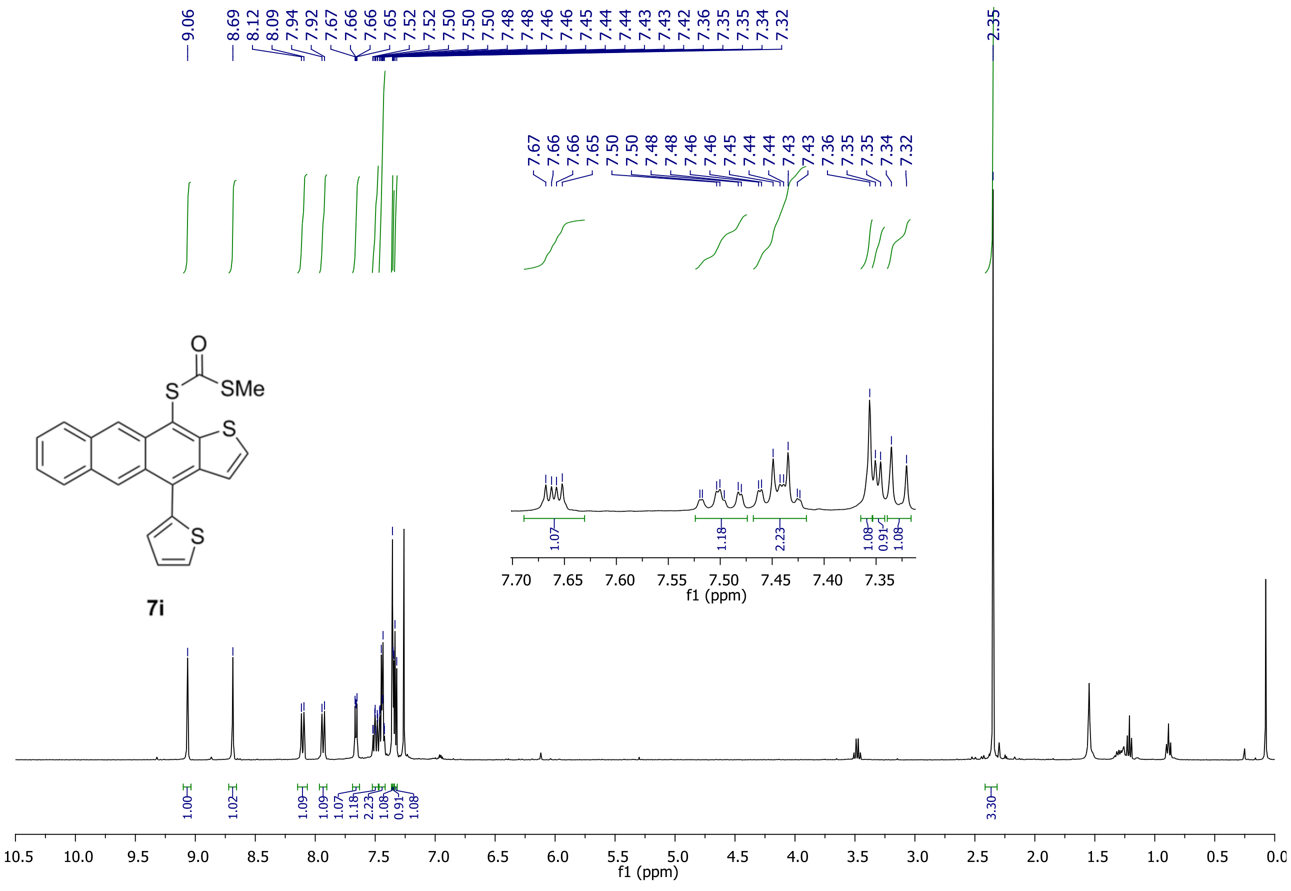

7i

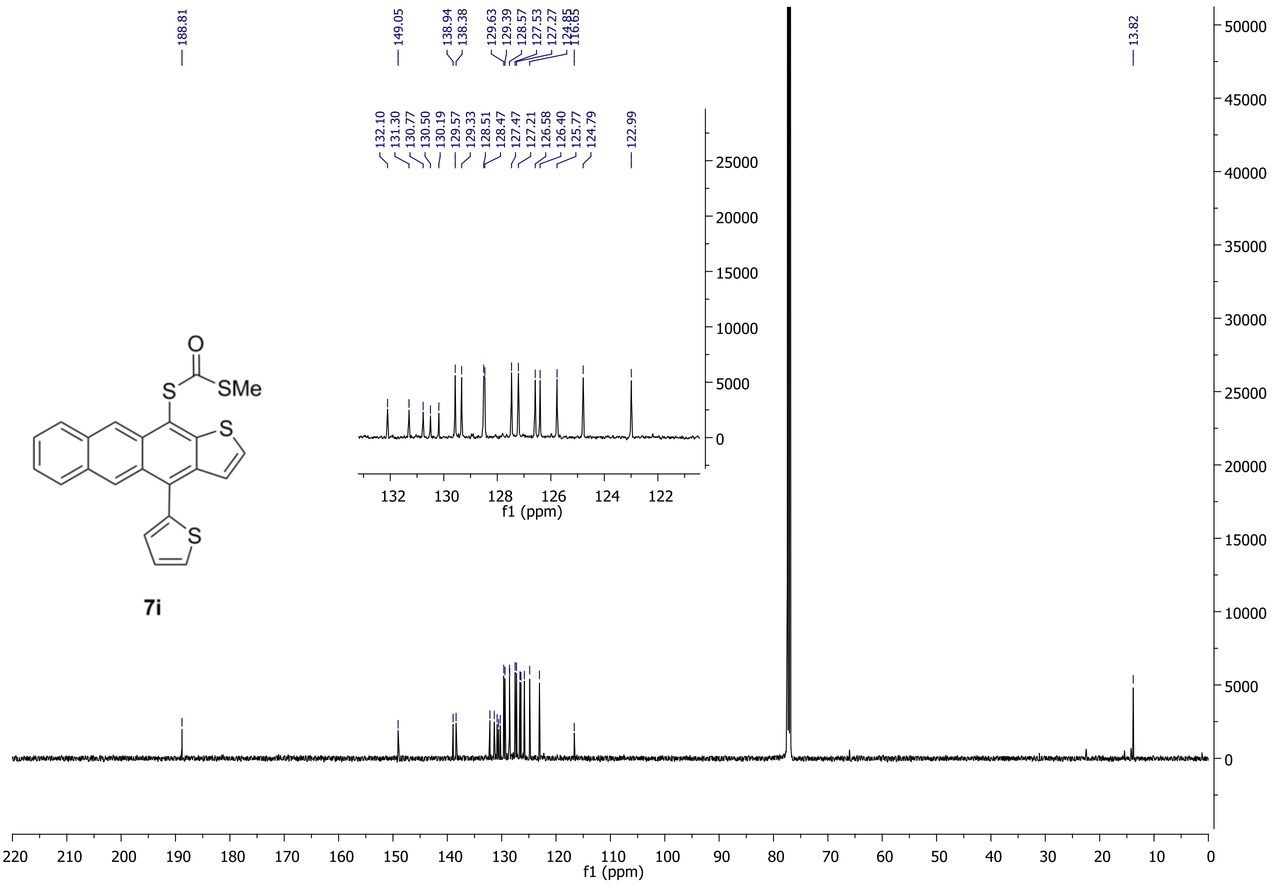

7i

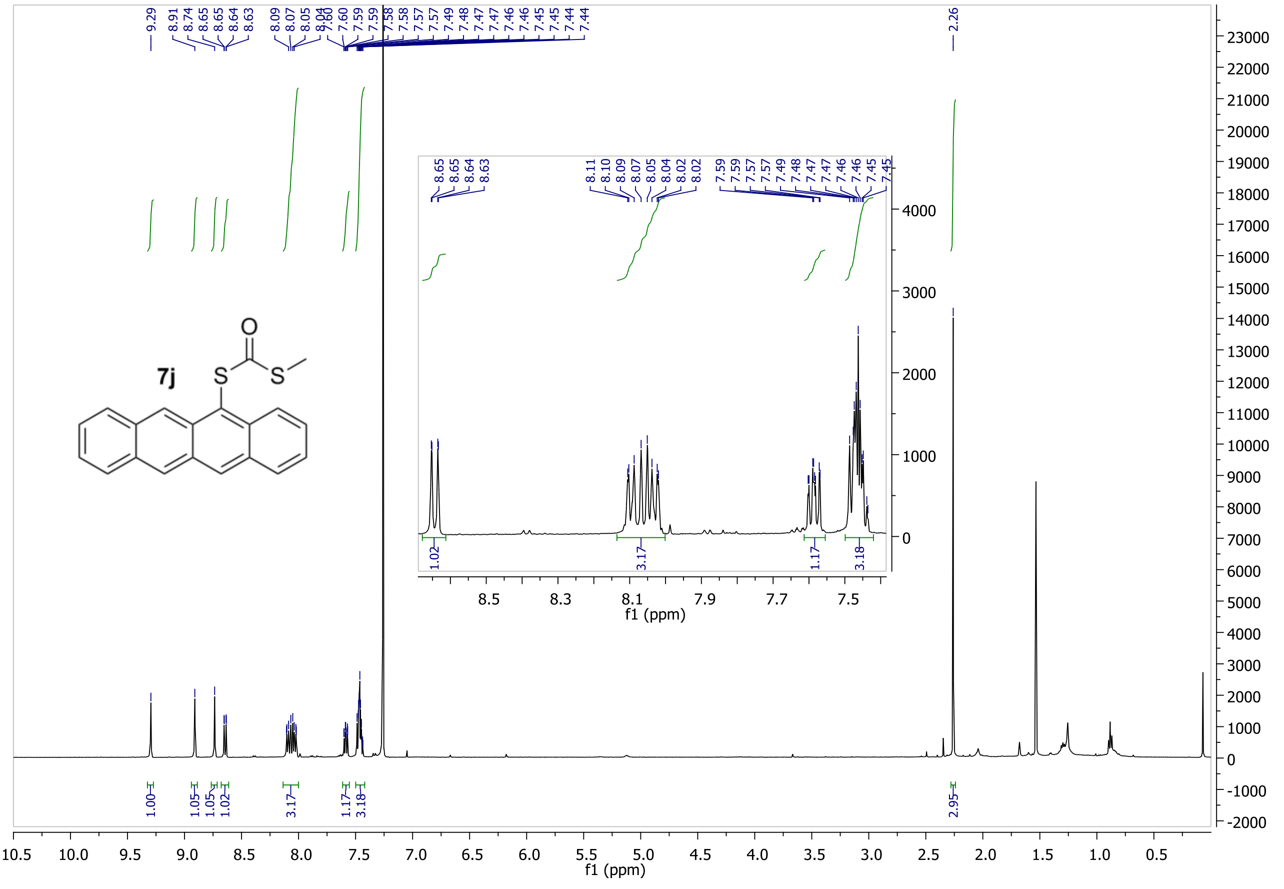

7j

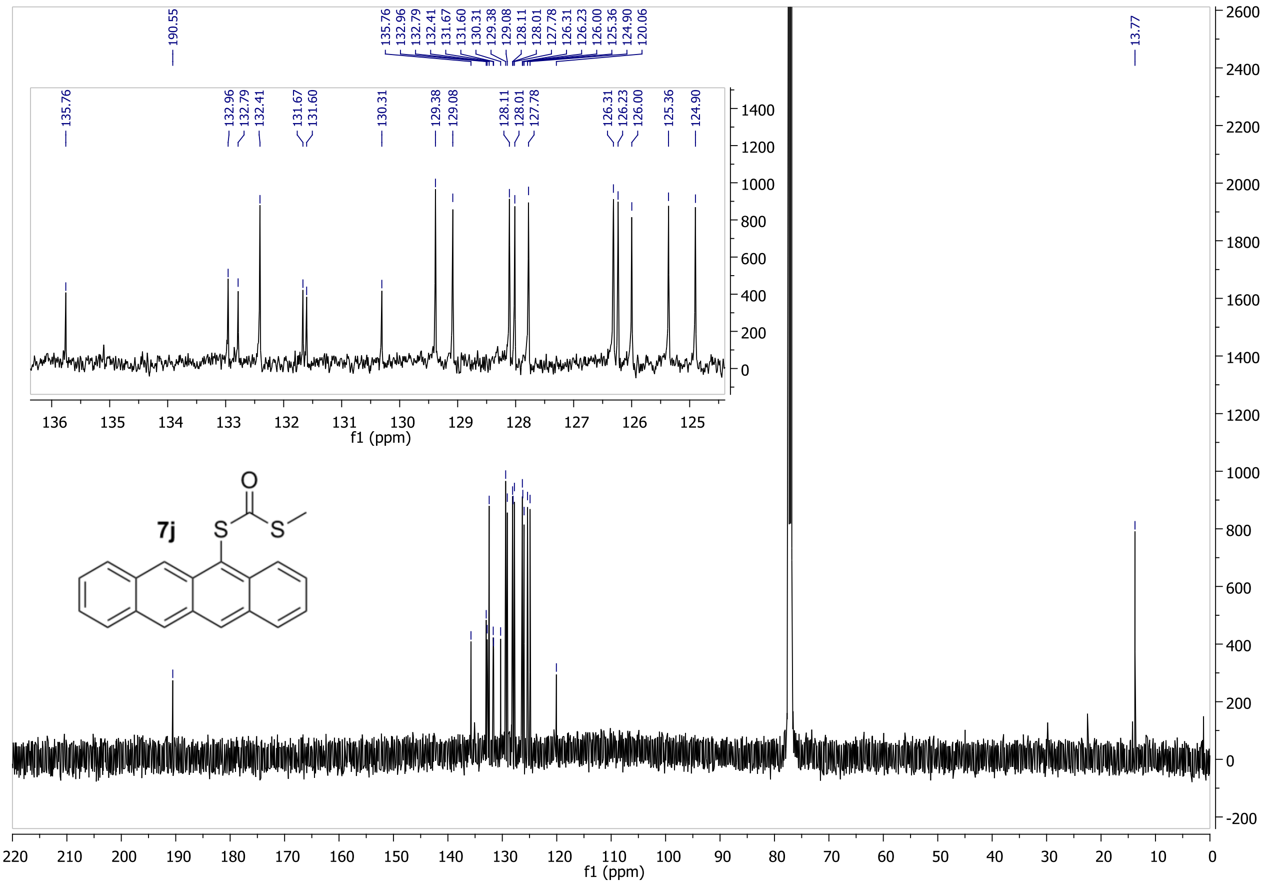
7j
O
S
S
190.55
135.76 132.96 132.79 132.41 131.67 131.60 130.31 129.38 129.08 128.11 128.01 127.78 126.31 126.23 126.00 125.36 124.90 120.06
13.77
f1 (ppm)
f1 (ppm)

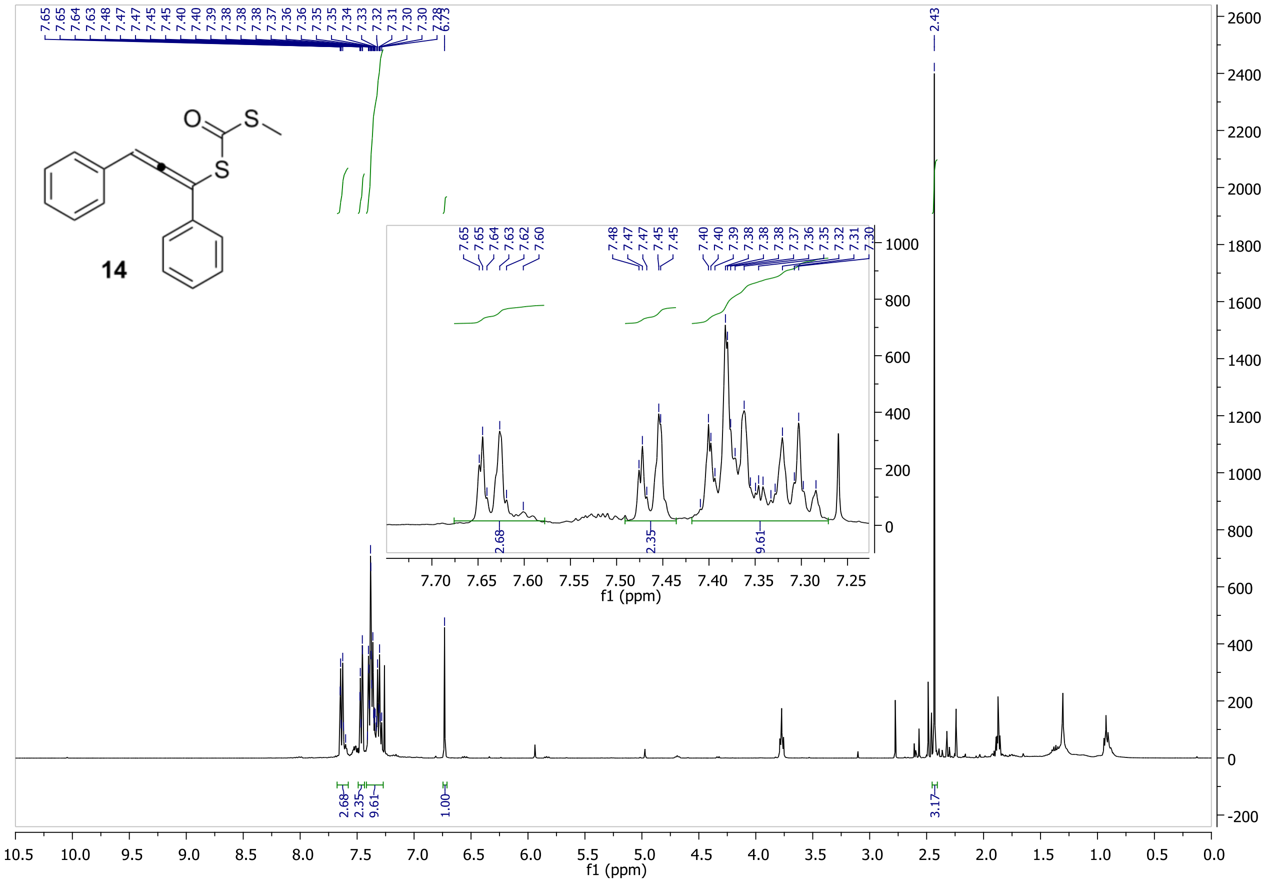
14
O
S
S
7.65 7.65 7.64 7.63 7.48 7.47 7.47 7.45 7.45 7.40 7.40 7.39 7.38 7.38 7.38 7.37 7.36 7.36 7.35 7.35 7.34 7.33 7.32 7.31 7.30 7.30 7.28 6.73
2.43
7.65 7.65 7.64 7.63 7.62 7.60 7.48 7.47 7.47 7.45 7.45 7.40 7.40 7.39 7.38 7.38 7.38 7.37 7.36 7.35 7.32 7.31 7.30
2.68
2.35
9.61
1.00
3.17
f1 (ppm)
f1 (ppm)

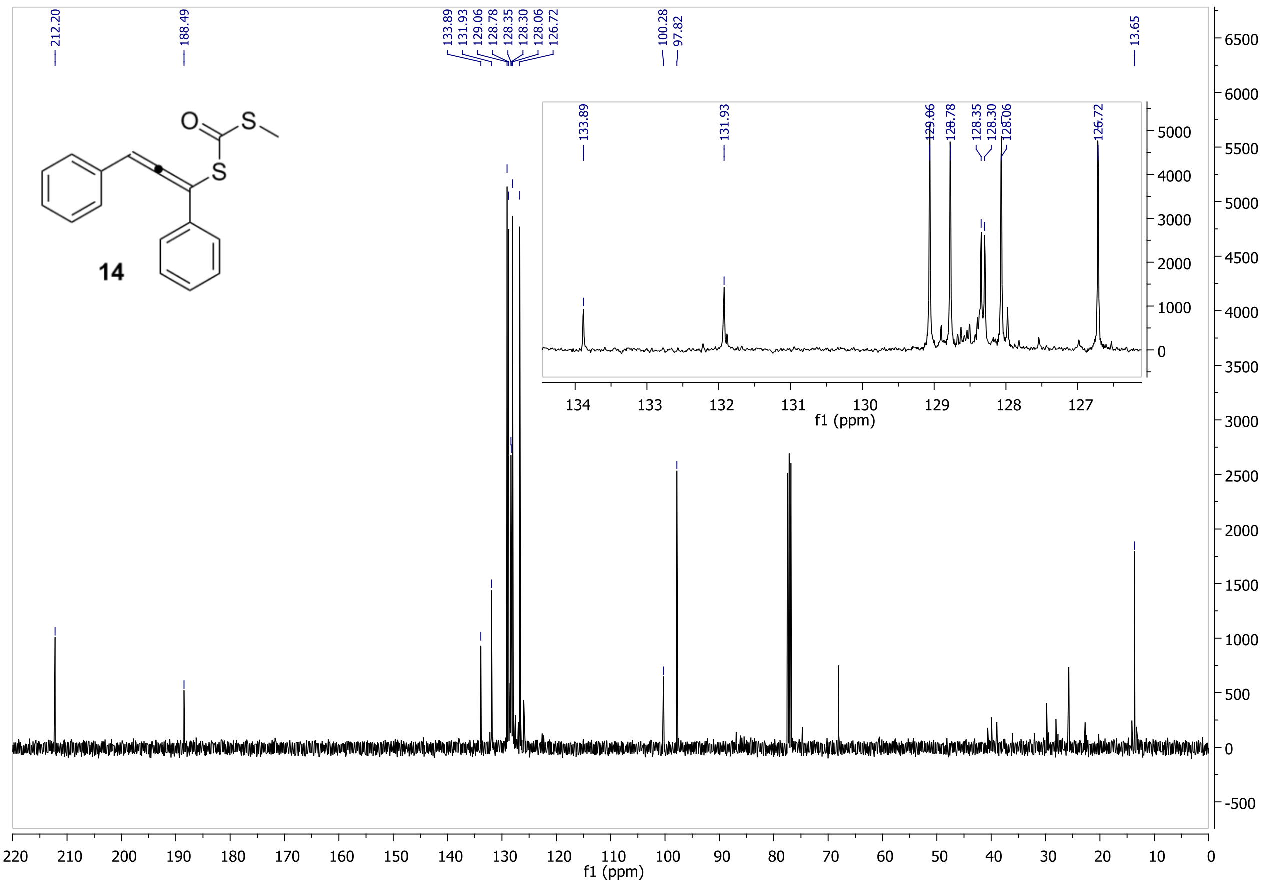

212.20
188.49
133.89
131.93
129.06
128.78
128.35
128.30
128.06
126.72
100.28
97.82
13.65
14
f1 (ppm)

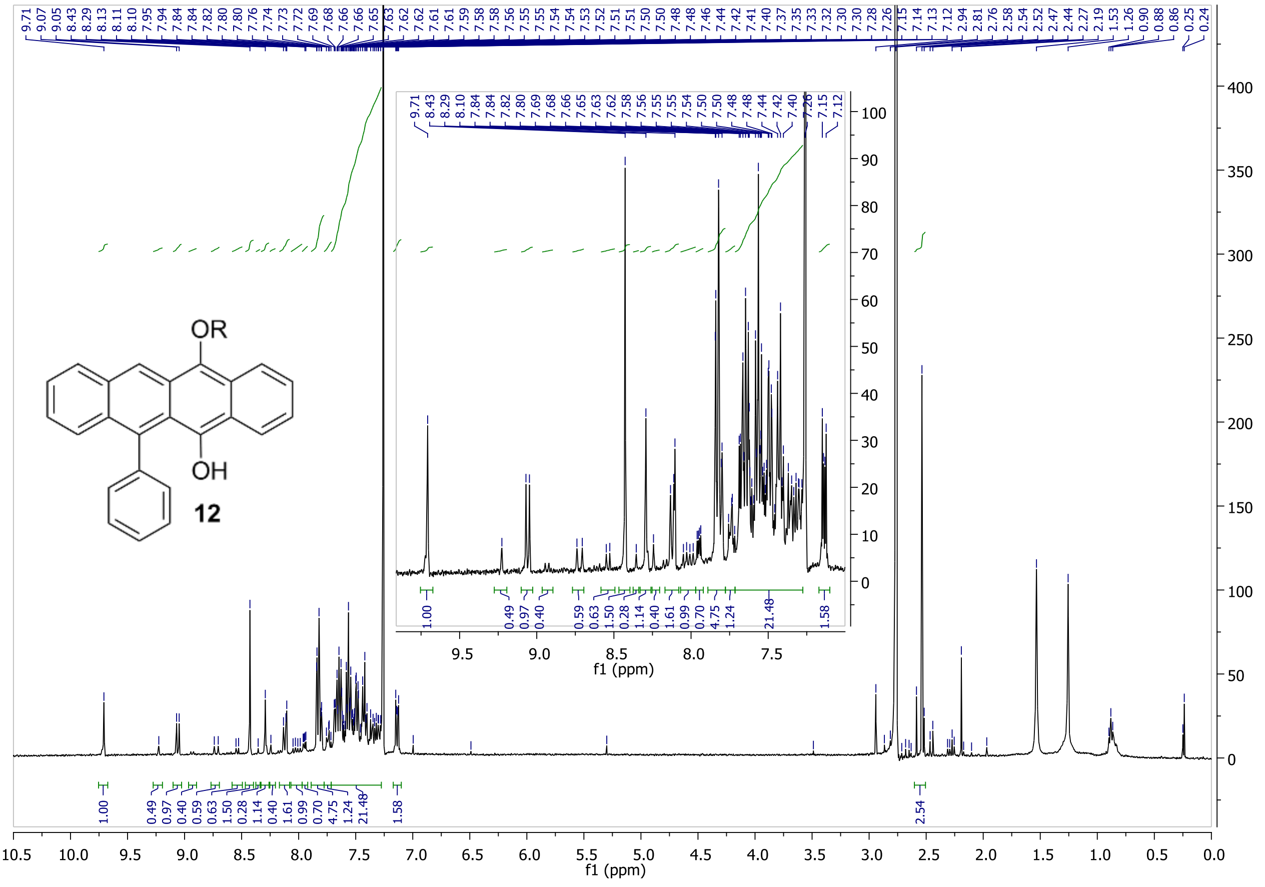

OR
OH
12
f1 (ppm)

7. References and notes

[1] Asikainen, M.; Lewis, W.; Blake, A. J.; Woodward, S. *Tetrahedron Lett.* **2010**, *51*, 6454-6456.
[2] Gottlieb H. E.; Kotlyar V.; Nudelman A. *J. Org. Chem.* **1997**, *62*, 7512-7515.
[3] Xia Y.; Jin, Y.; Kaur, N.; Choi, Y.; Lee, K. *Euro. J. Med. Chem.* **2011**, *46*, 2386-2396.
[4] Rodríguez, D.; Castedo, L.; Domínguez, D.; Saá, C. *Org. Lett.* **2003**, *5*, 3119-3121.
[5] Sun, H.; DiMagno, S. D. *J. Am Chem. Soc.* **2005**, *127*, 2050-2051.
[6] Chen, M.; Chen, Y.; Liu, Y. *Chem. Commun*. **2012**, *48*, 12189-1219.
[7] Lin, Y.-C.; Lin, C.-H. *Org. Lett.* **2007**, *9*, 2075-2078.
[8] Bredas, J. L., Silbey, R., Boudreaux, D. S., Chance, R. R., *J. Am. Chem. Soc*. **1983**, *105*, 6555-6559.
[9] Tauc, J. *Mater. Res. Bull.* **1968**, *3*, 37-46.
[10] Becke, A. D. *J. Chem. Phys.*, **1993**, *98*, 5648-5652.
[11] http://www.nsccs.ac.uk/
[12] Details of the DIAMOND light source can be found at: www.diamond.ac.uk/Home.html
[13] Structural database codes accessible via http://webcsd.ccdc.cam.ac.uk/index.php
[14] Molecular van der Waals volumes of the substituents were estimated using the method of Abraham: Zhao, Y.H; Abraham, M. H.; Zissimos, A. M. *J. Org. Chem.* **2003**, *68*, 7368-7373.
